# Supplementary material for: NELF prevents transcriptional readthrough into DNA replication zones in cancer cells
Source: EMBO Rep. 2026 Feb 20;27(7):1720–58. doi: 10.1038/s44319-026-00700-z (PMC13076867; doi:10.1038/s44319-026-00700-z)
Supplement: Supplementary file 1 — Table EV1 [file 44319_2026_700_MOESM1_ESM.pdf]

Table EV1. GTEx and TCGA sample size for Figs. 1A-B and EV1A-D

GTEx tissue / TCGA cancer type	Normal (GTEx + TCGA normal)	Tumor (TCGA tumor)	Total
breast/brca	199	982	1181
colon-sigmoid / read	153	87	240
colon-transverse / coad	235	285	520
liver / lihc	163	295	458
salivary gland / hnsc	97	460	557
esophageal / esca	672	183	855
prostate / prad	154	426	580
stomach / stad	225	380	605
thyroid / thca	371	441	812
uterus/ucec	105	141	246
lung / luad	372	503	875
**Total**	2746	4183	6929
