# Supplementary material for: NELF prevents transcriptional readthrough into DNA replication zones in cancer cells
Source: EMBO Rep. 2026 Feb 20;27(7):1720–58. doi: 10.1038/s44319-026-00700-z (PMC13076867; doi:10.1038/s44319-026-00700-z)
Supplement: Supplementary file 2 — Table EV2 [file 44319_2026_700_MOESM2_ESM.pdf]

Table EV2. PCR primer sequences for Figs. EV3B and EV8G

Name	Sequence	Figure
RPS23_intron_F	AGGACTTCAGCTCAGCCATTT	Fig EV3B
RPS23_intron_R	ACTGTGAGCTGGCTTTCTGAA	Fig EV3B
RPS23_dwnstr_F	TGGCCAACACCTGAAATGACT	Fig EV3B
RPS23_dwnstr_R	TTCACTGTCACAAAGCCACCT	Fig EV3B
HELLS_intron_F	AGACAGTTTTGCCATGTTGCC	Fig EV3B
HELLS_intron_R	AGAATATGACAGTCCAGGCGC	Fig EV3B
HELLS_dwnstr_F	ACGAGGAAGTAGCTCAAAGGC	Fig EV3B
HELLS_dwnstr_R	ACCAGGCCCTACTCTTCTCTT	Fig EV3B
CLIC4_dwnstr_F	AGACTCATTCTCACGTGGCTG	Fig EV3B
CLIC4_dwnstr_R	CCTCCGTGTCCAGCCTTTAAA	Fig EV3B
CLIC4_intron_F	AGAGTTGCCGTTCATGGAGAG	Fig EV3B
CLIC4_intron_R	CTCCACTTGACATCTCAGCCA	Fig EV3B
ELAVL2_F	AGCTGGGATCAACCTTTTCCTC	Fig EV8G
ELAVL2_R	AATTTGCCACTGTGGTGCTC	Fig EV8G
FOCAD_F	AACCAGCAGCATTCTCAGCT	Fig EV8G
FOCAD_R	AGGCATGTGTCAAGCTGTCA	Fig EV8G
up_IFNB1_F	GCCAGTGTTTCTCCCAAAGC	Fig EV8G
up_IFNB1_R	GCACAGGGCCACAATAGCTA	Fig EV8G
IFNB14_F	GAATGACCAAATGGGCTGGG	Fig EV8G
IFNB14_R	CGTGATCCATCTGCCTTGGT	Fig EV8G
