# Supplementary figures and images for: NELF prevents transcriptional readthrough into DNA replication zones in cancer cells
Source: EMBO Rep. 2026 Feb 20;27(7):1720–58. doi: 10.1038/s44319-026-00700-z (PMC13076867; doi:10.1038/s44319-026-00700-z)

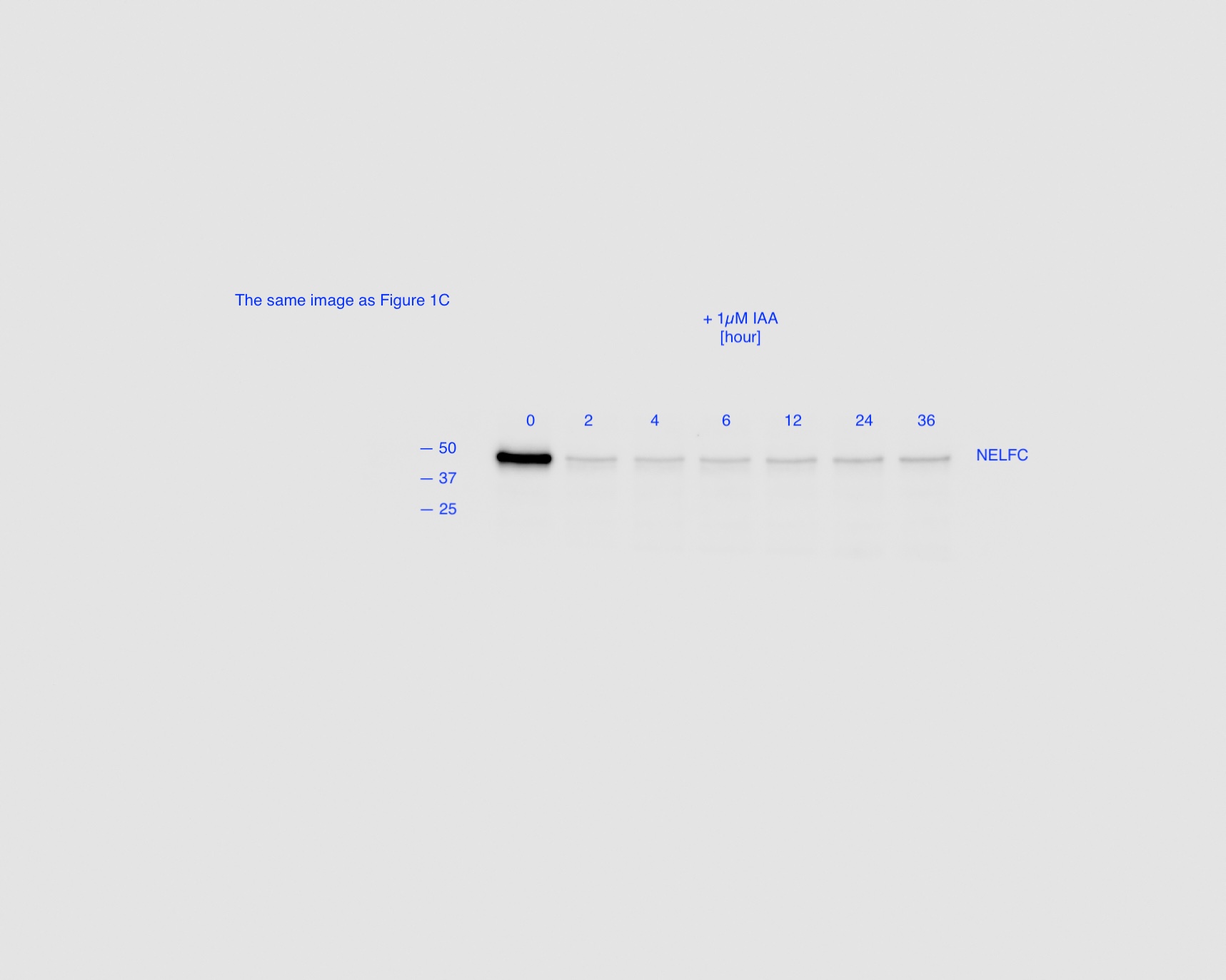

Supplement: Supplementary file 4 — Source data Fig. 1 [file 44319_2026_700_MOESM4_ESM.zip › Figure 1 -revised/1G - Western blot/NELFC(Chemiluminescence).jpg]

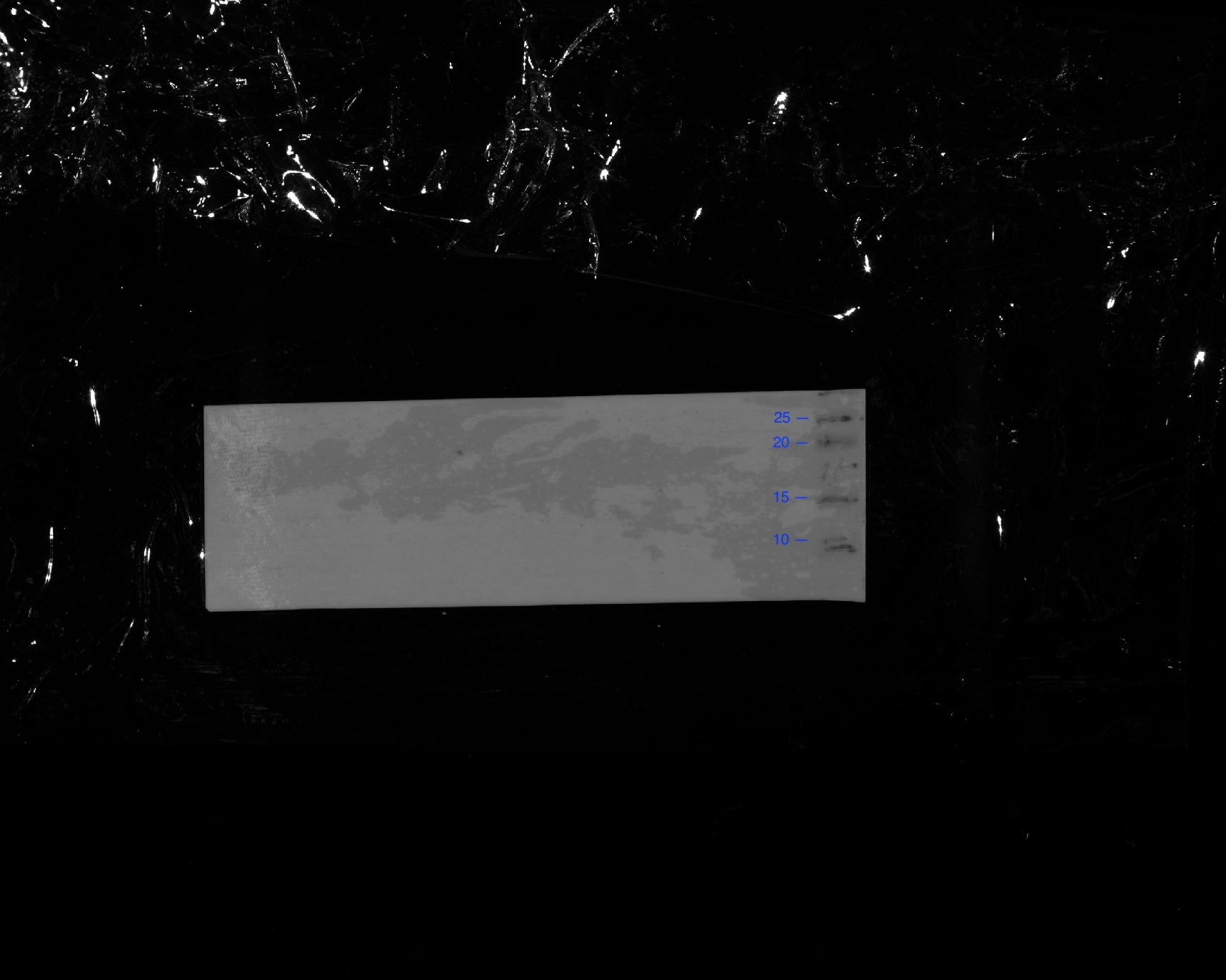

Supplement: Supplementary file 4 — Source data Fig. 1 [file 44319_2026_700_MOESM4_ESM.zip › Figure 1 -revised/1G - Western blot/P21(Ponceau S).jpg]

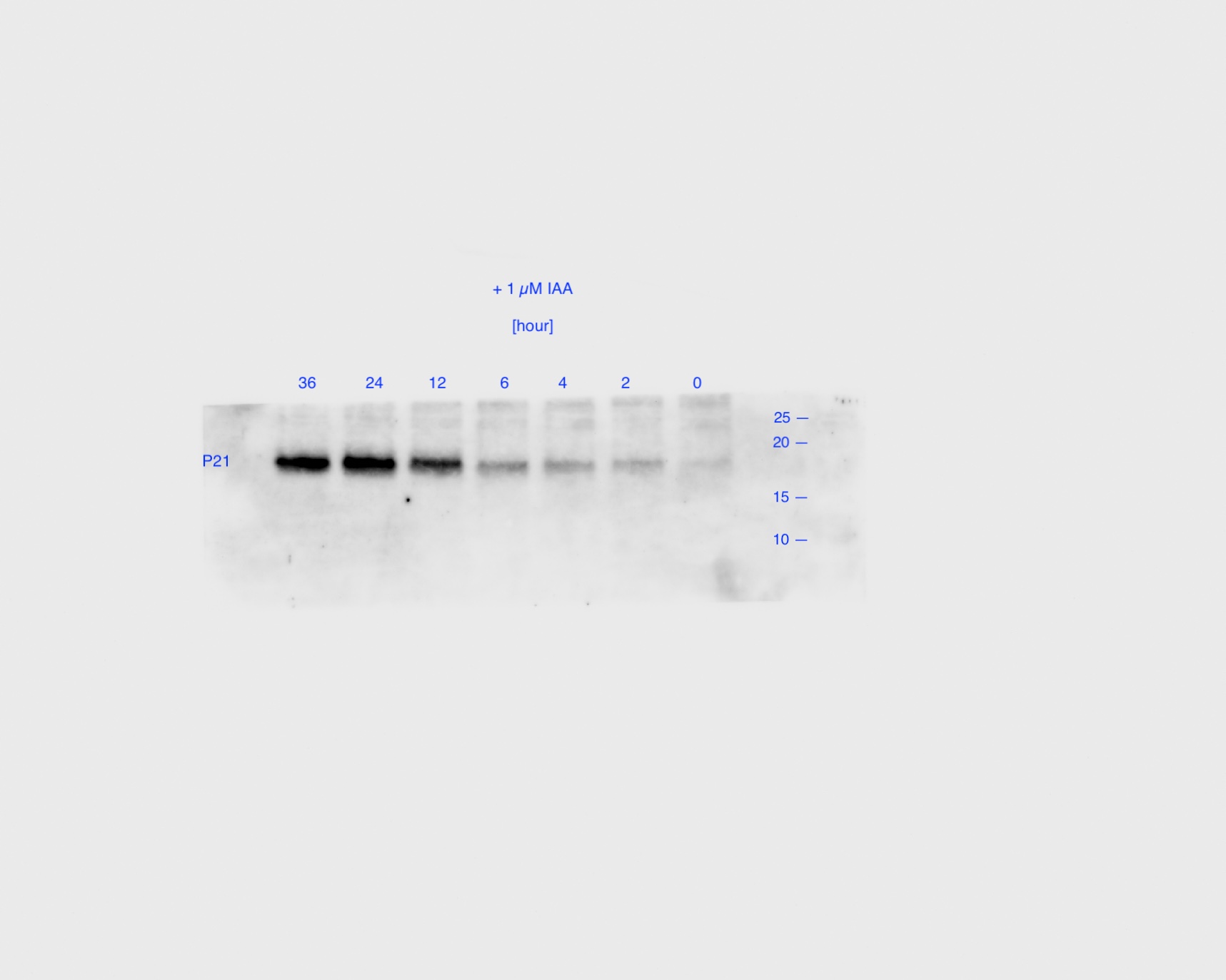

Supplement: Supplementary file 4 — Source data Fig. 1 [file 44319_2026_700_MOESM4_ESM.zip › Figure 1 -revised/1G - Western blot/P21(Chemiluminescence).jpg]

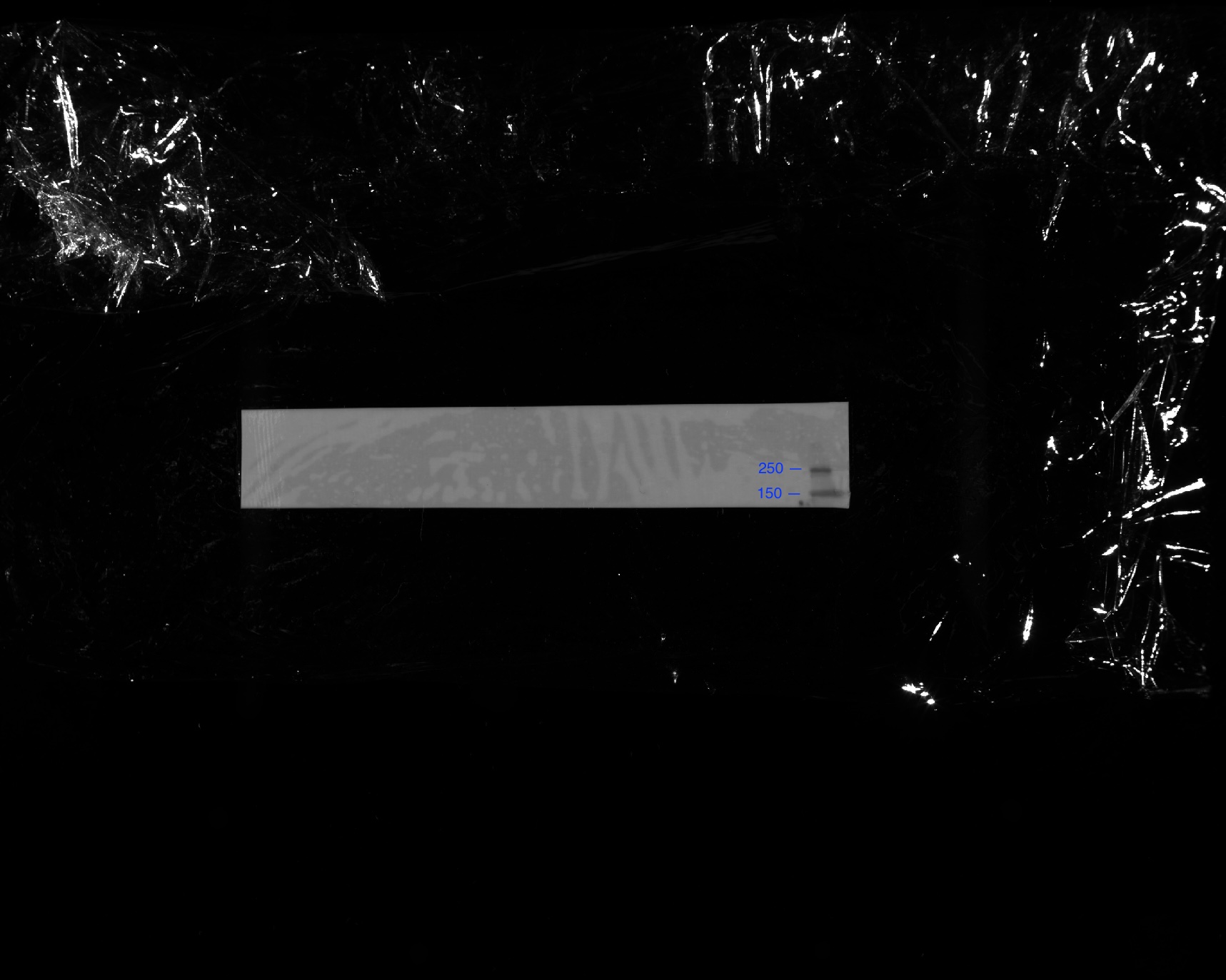

Supplement: Supplementary file 4 — Source data Fig. 1 [file 44319_2026_700_MOESM4_ESM.zip › Figure 1 -revised/1G - Western blot/Pol I(Ponceau S).jpg]

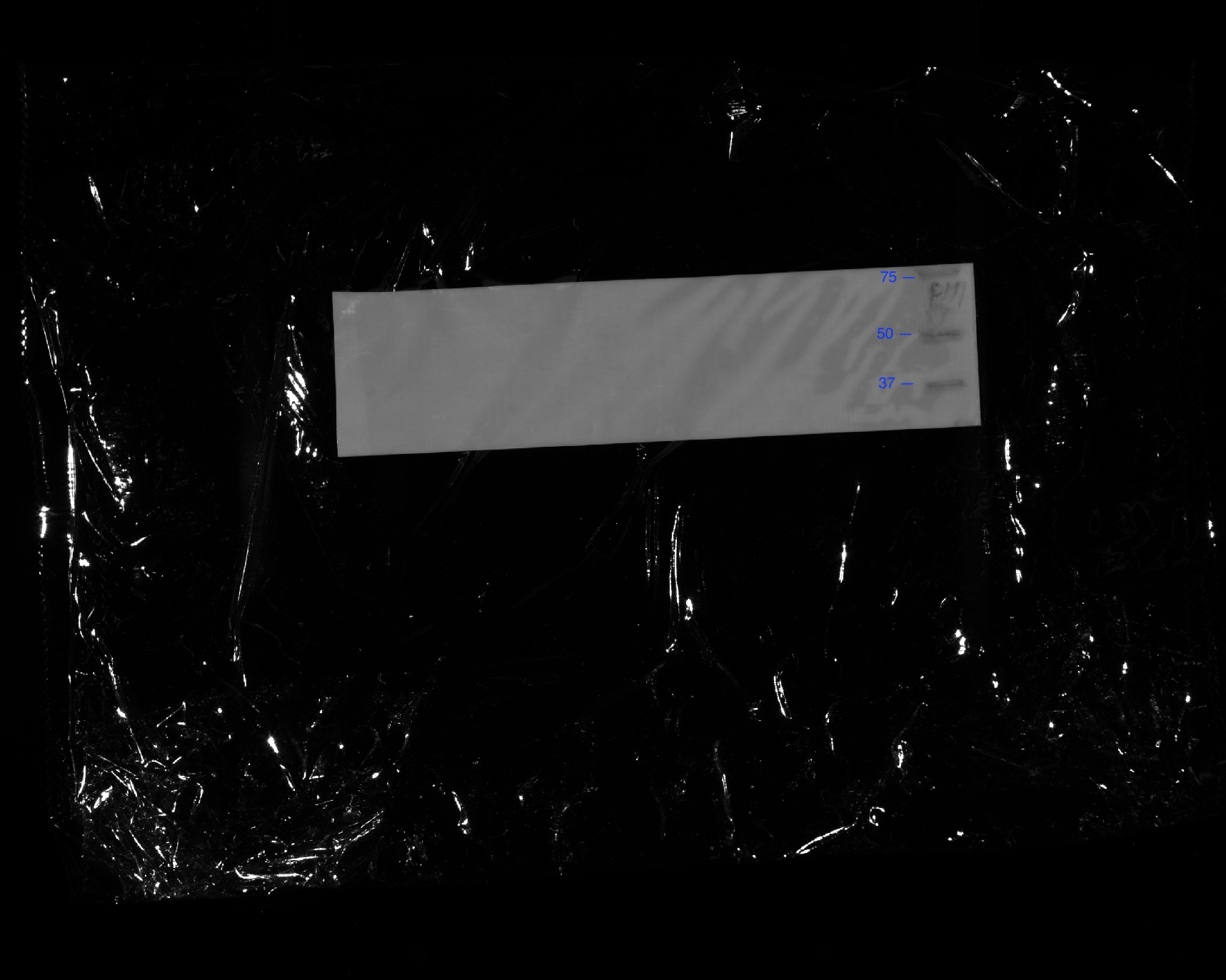

Supplement: Supplementary file 4 — Source data Fig. 1 [file 44319_2026_700_MOESM4_ESM.zip › Figure 1 -revised/1G - Western blot/P57(Ponceau S).jpg]

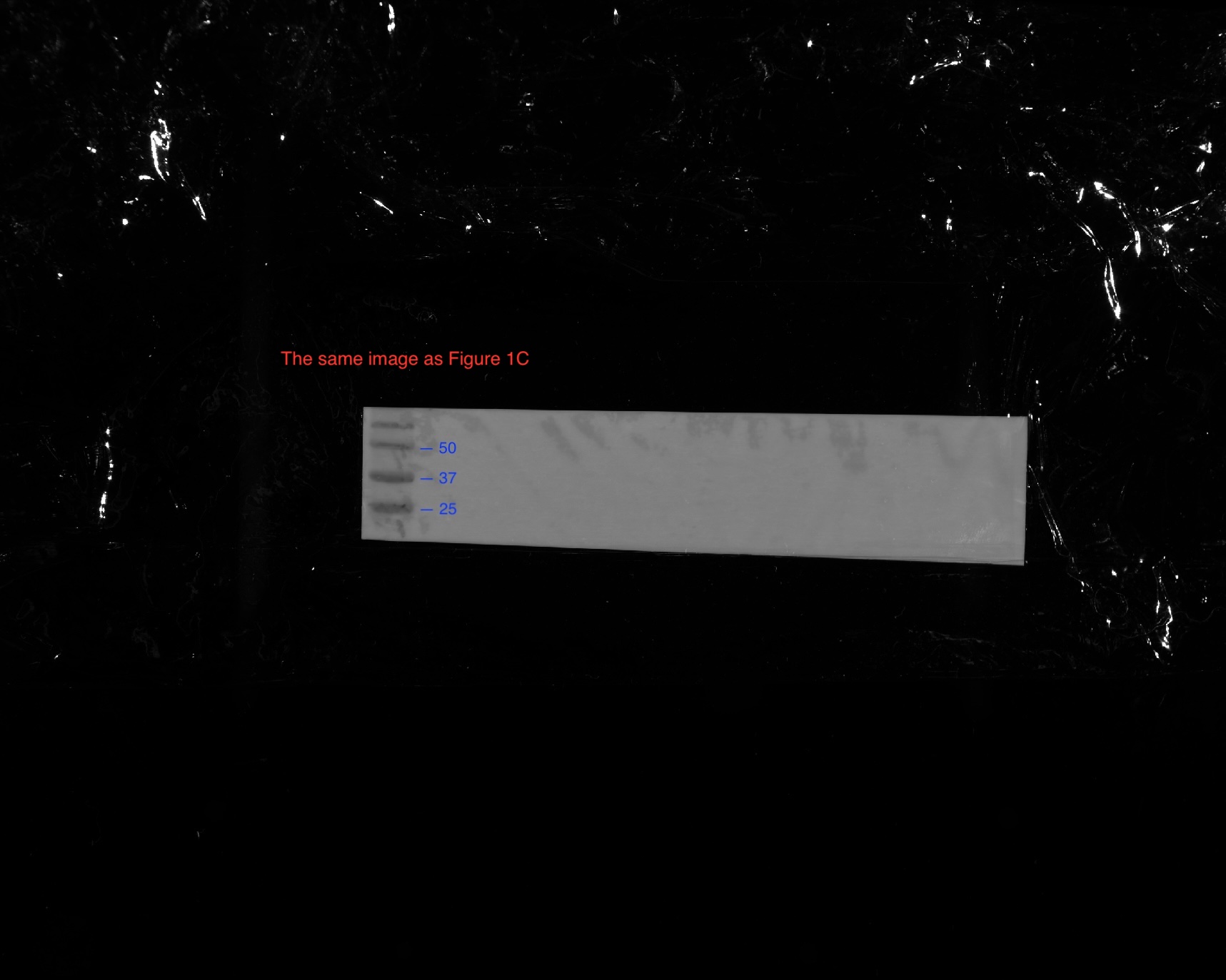

Supplement: Supplementary file 4 — Source data Fig. 1 [file 44319_2026_700_MOESM4_ESM.zip › Figure 1 -revised/1G - Western blot/NELFC(Ponceau S).jpg]

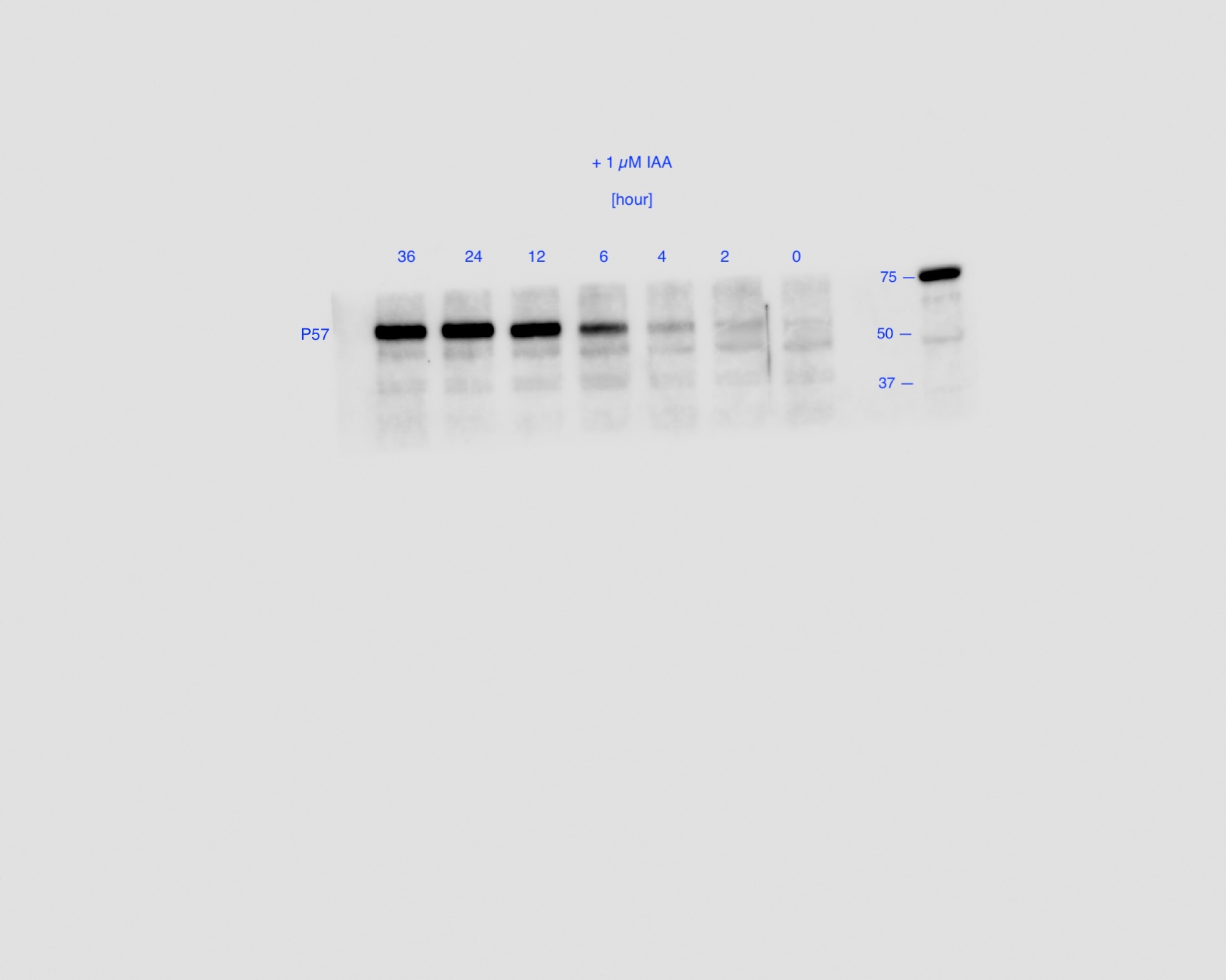

Supplement: Supplementary file 4 — Source data Fig. 1 [file 44319_2026_700_MOESM4_ESM.zip › Figure 1 -revised/1G - Western blot/P57(Chemiluminescence).jpg]

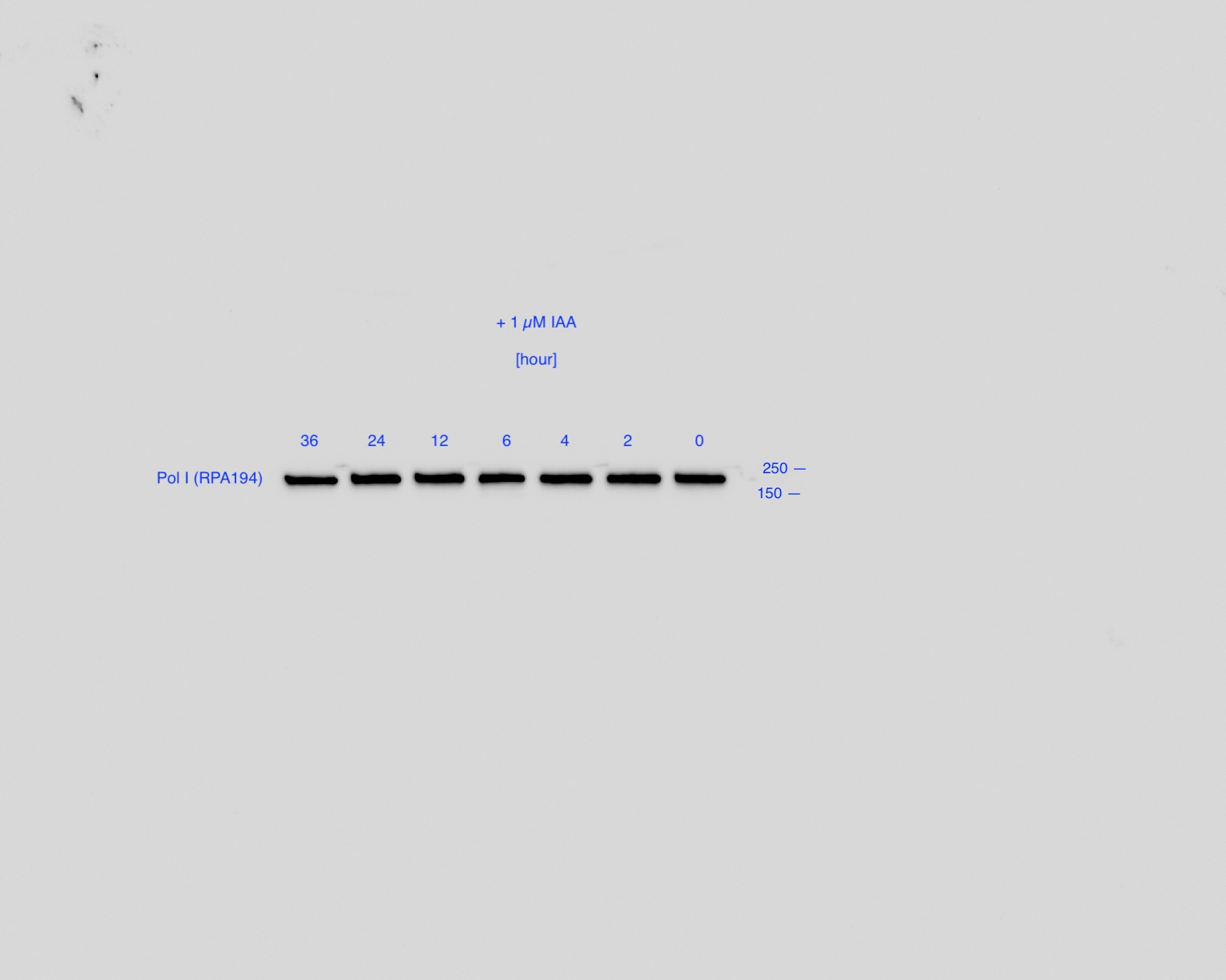

Supplement: Supplementary file 4 — Source data Fig. 1 [file 44319_2026_700_MOESM4_ESM.zip › Figure 1 -revised/1G - Western blot/Pol I(Chemiluminescence).jpg]

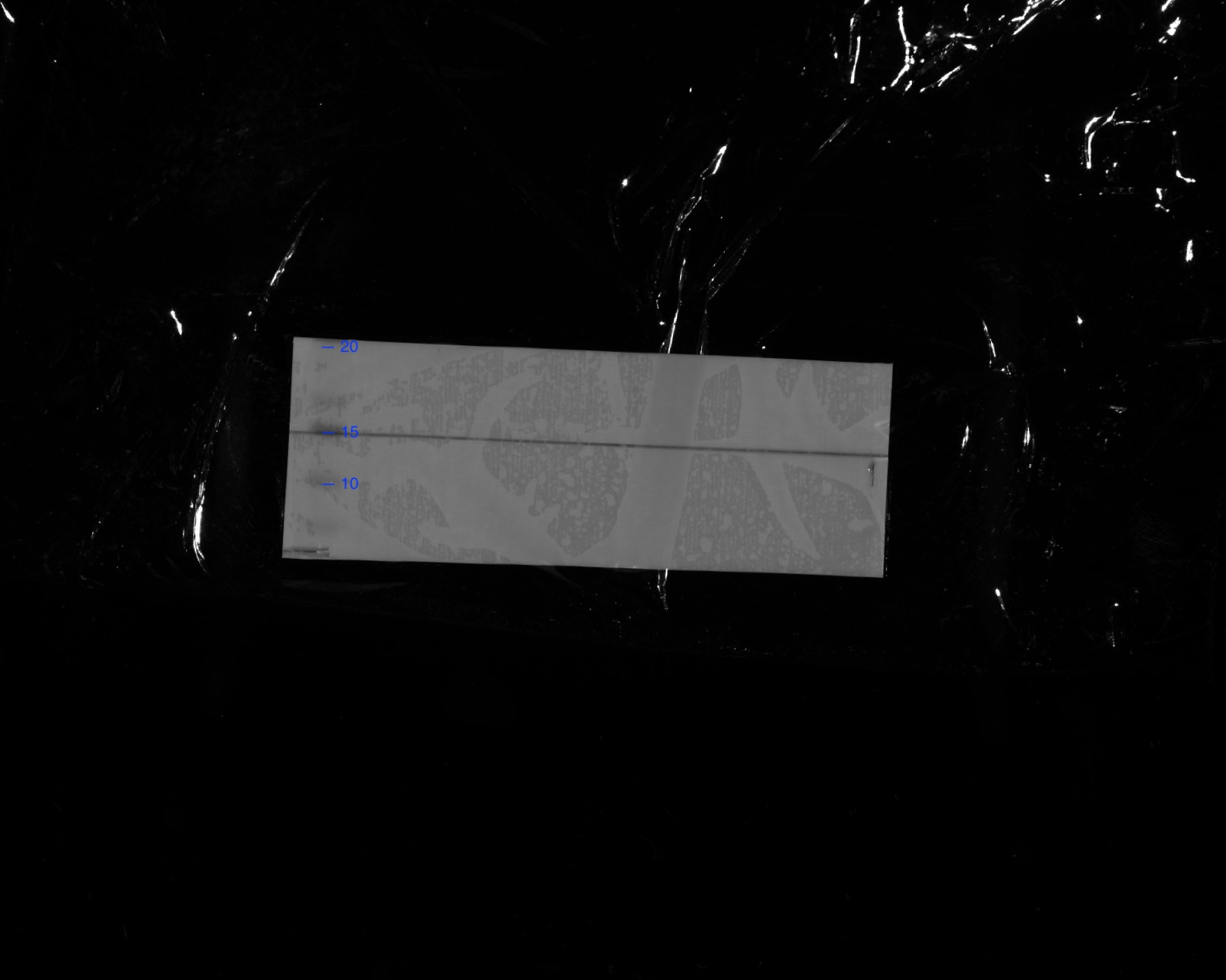

Supplement: Supplementary file 4 — Source data Fig. 1 [file 44319_2026_700_MOESM4_ESM.zip › Figure 1 -revised/1D - Western blot/SPT4 H2AXp(Ponceau S).jpg]

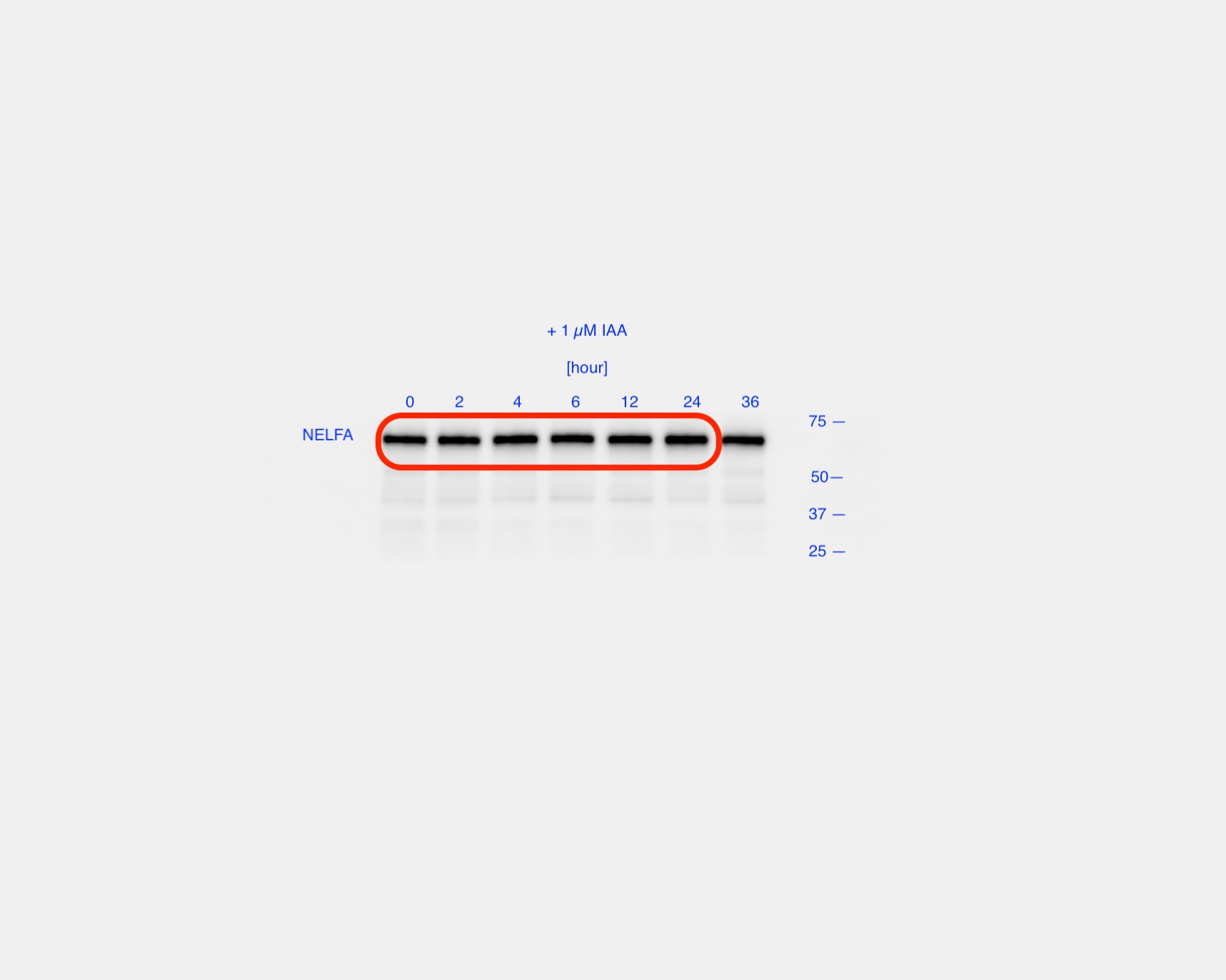

Supplement: Supplementary file 4 — Source data Fig. 1 [file 44319_2026_700_MOESM4_ESM.zip › Figure 1 -revised/1D - Western blot/NELFA(Chemiluminescence).jpg]

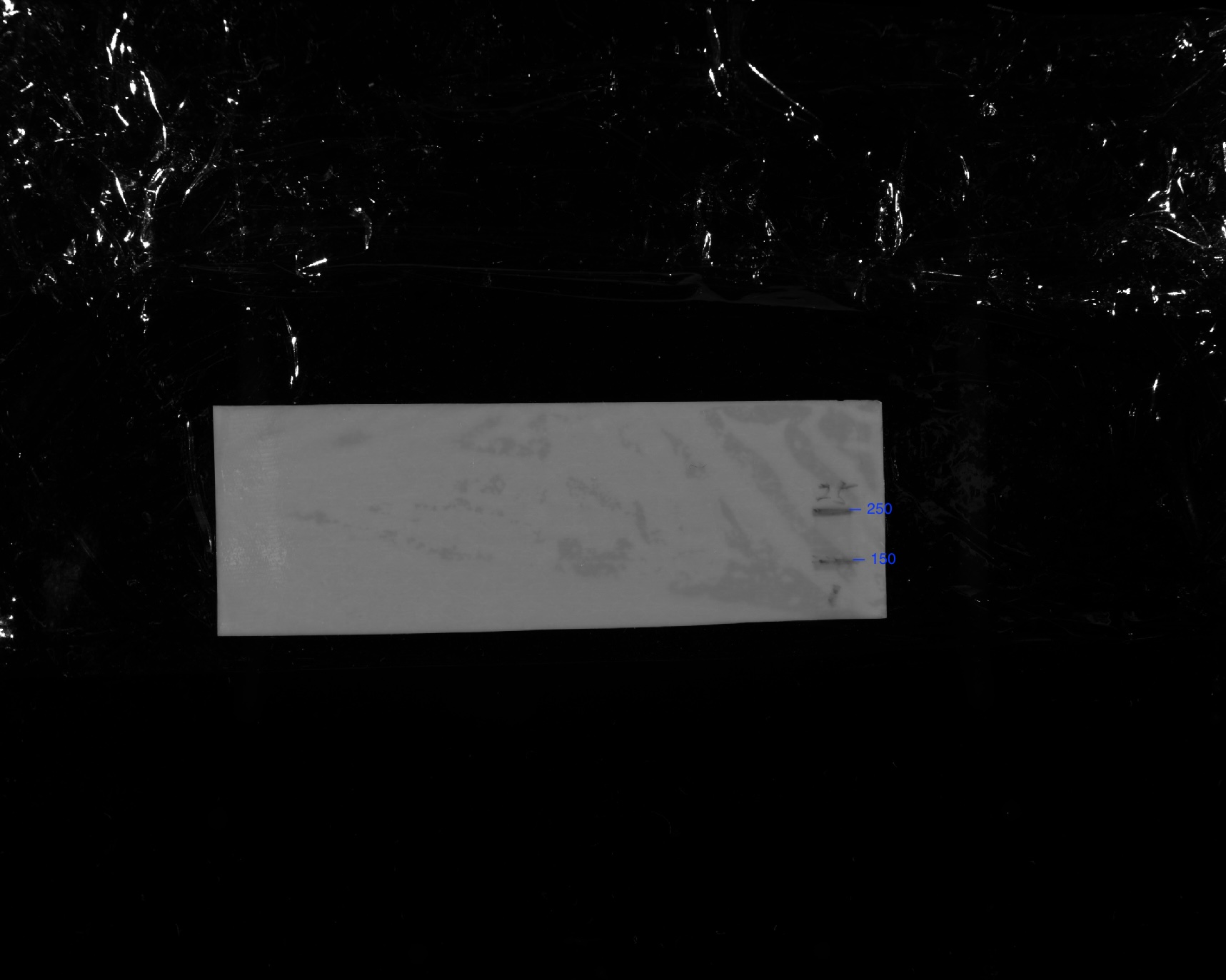

Supplement: Supplementary file 4 — Source data Fig. 1 [file 44319_2026_700_MOESM4_ESM.zip › Figure 1 -revised/1D - Western blot/Pol II(Ponceau S).jpg]

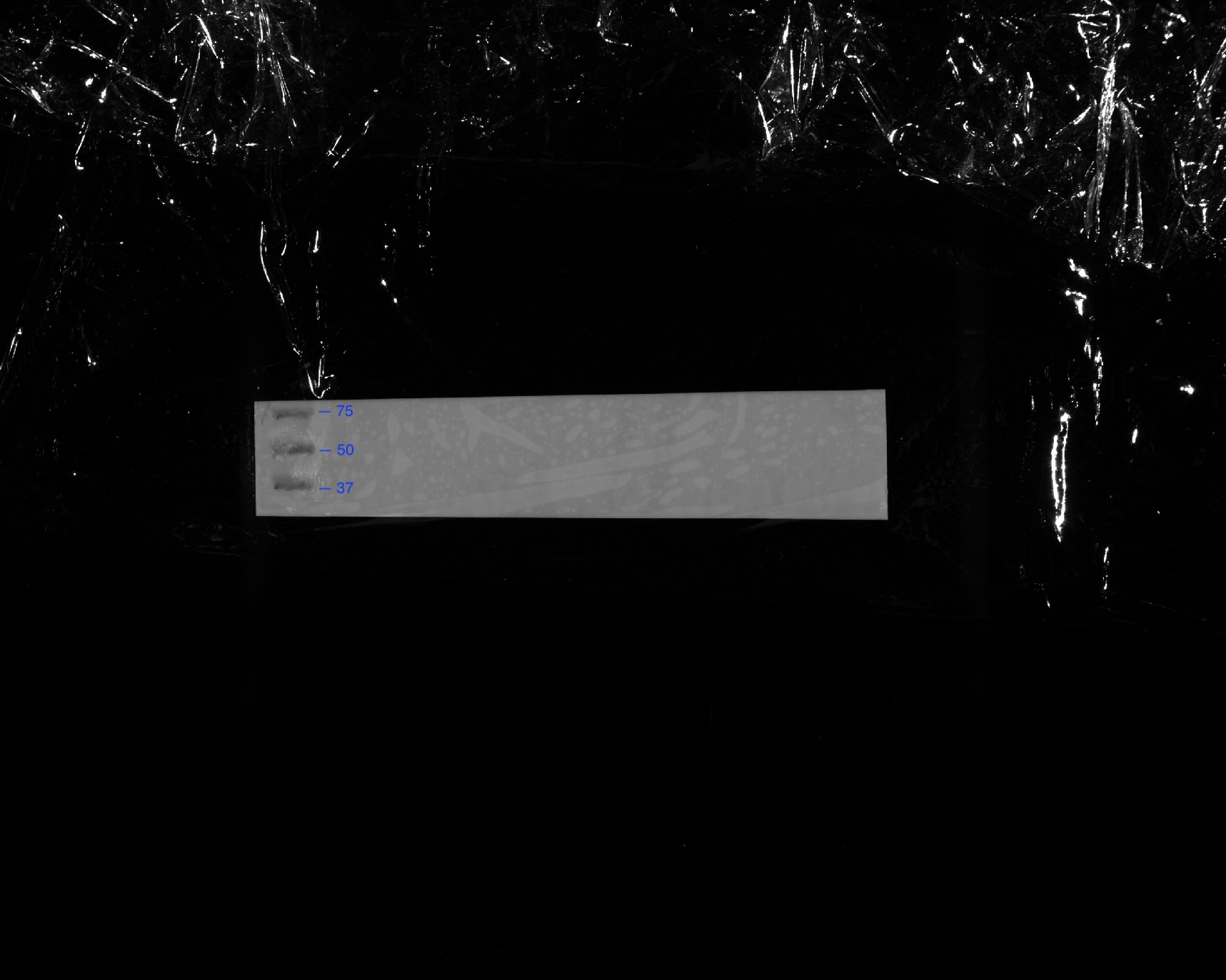

Supplement: Supplementary file 4 — Source data Fig. 1 [file 44319_2026_700_MOESM4_ESM.zip › Figure 1 -revised/1D - Western blot/TUB(Ponceau S).jpg]

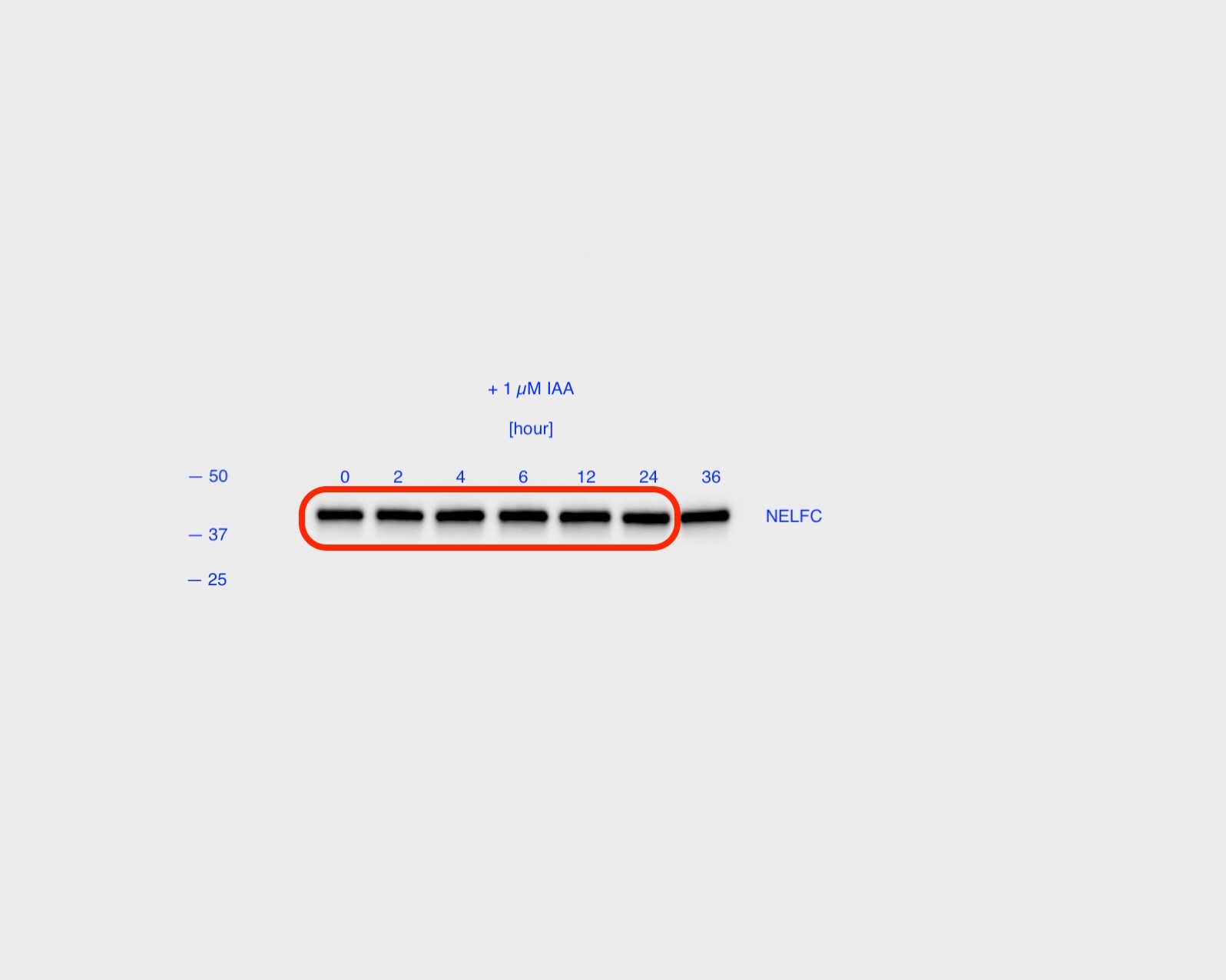

Supplement: Supplementary file 4 — Source data Fig. 1 [file 44319_2026_700_MOESM4_ESM.zip › Figure 1 -revised/1D - Western blot/NELFC(Chemiluminescence).jpg]

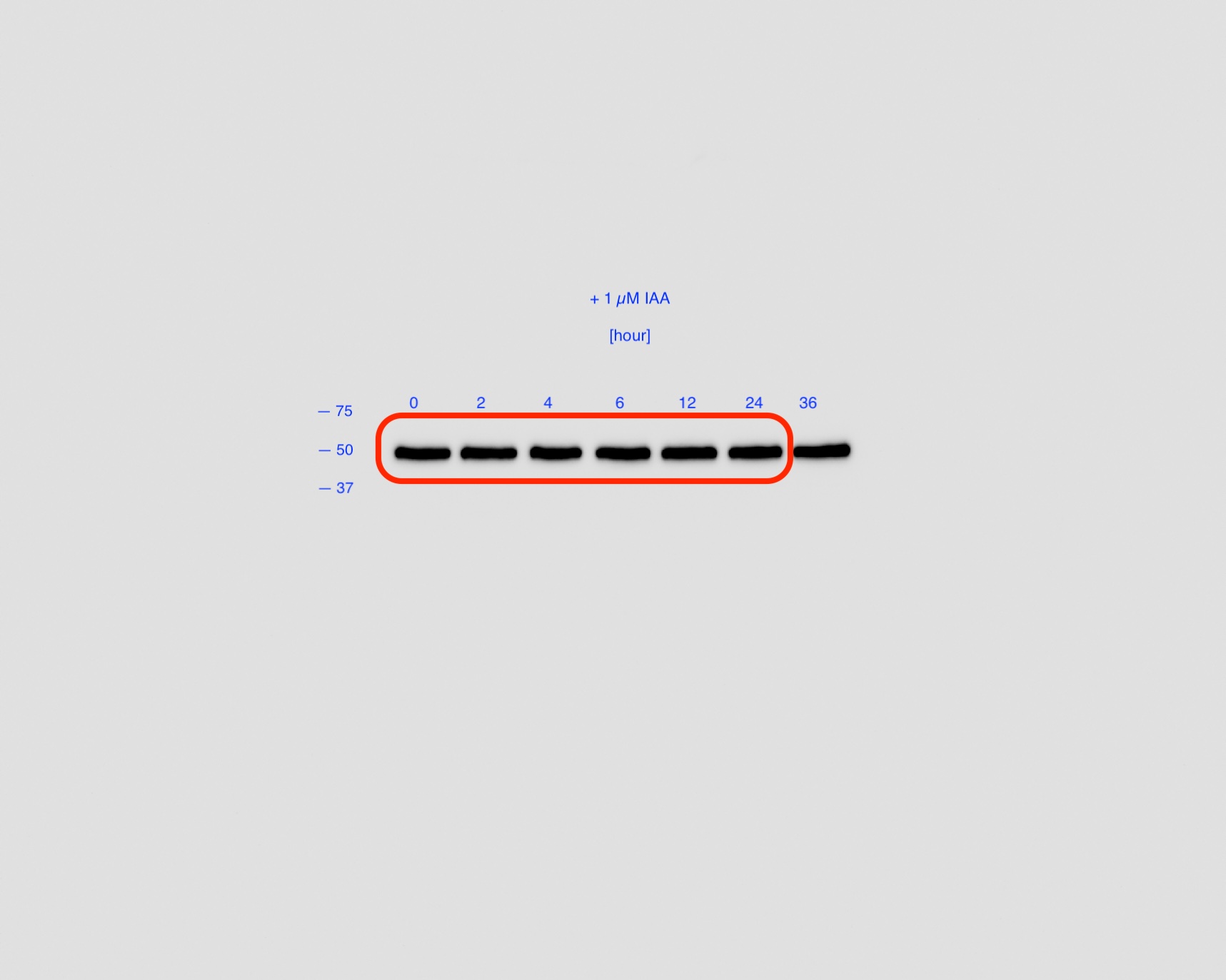

Supplement: Supplementary file 4 — Source data Fig. 1 [file 44319_2026_700_MOESM4_ESM.zip › Figure 1 -revised/1D - Western blot/TUB(Chemiluminescence).jpg]

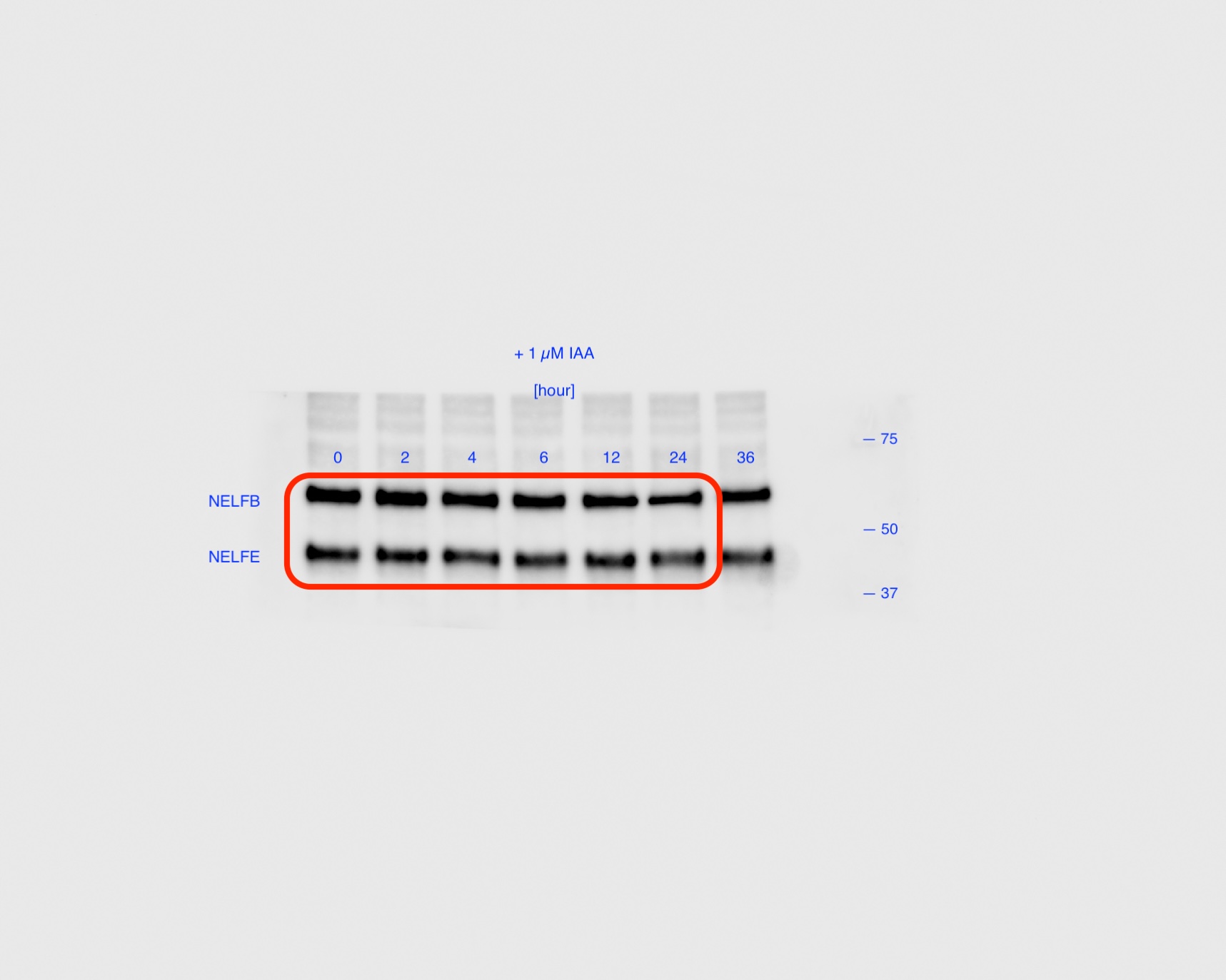

Supplement: Supplementary file 4 — Source data Fig. 1 [file 44319_2026_700_MOESM4_ESM.zip › Figure 1 -revised/1D - Western blot/NELFB NELFE(Chemiluminescence).jpg]

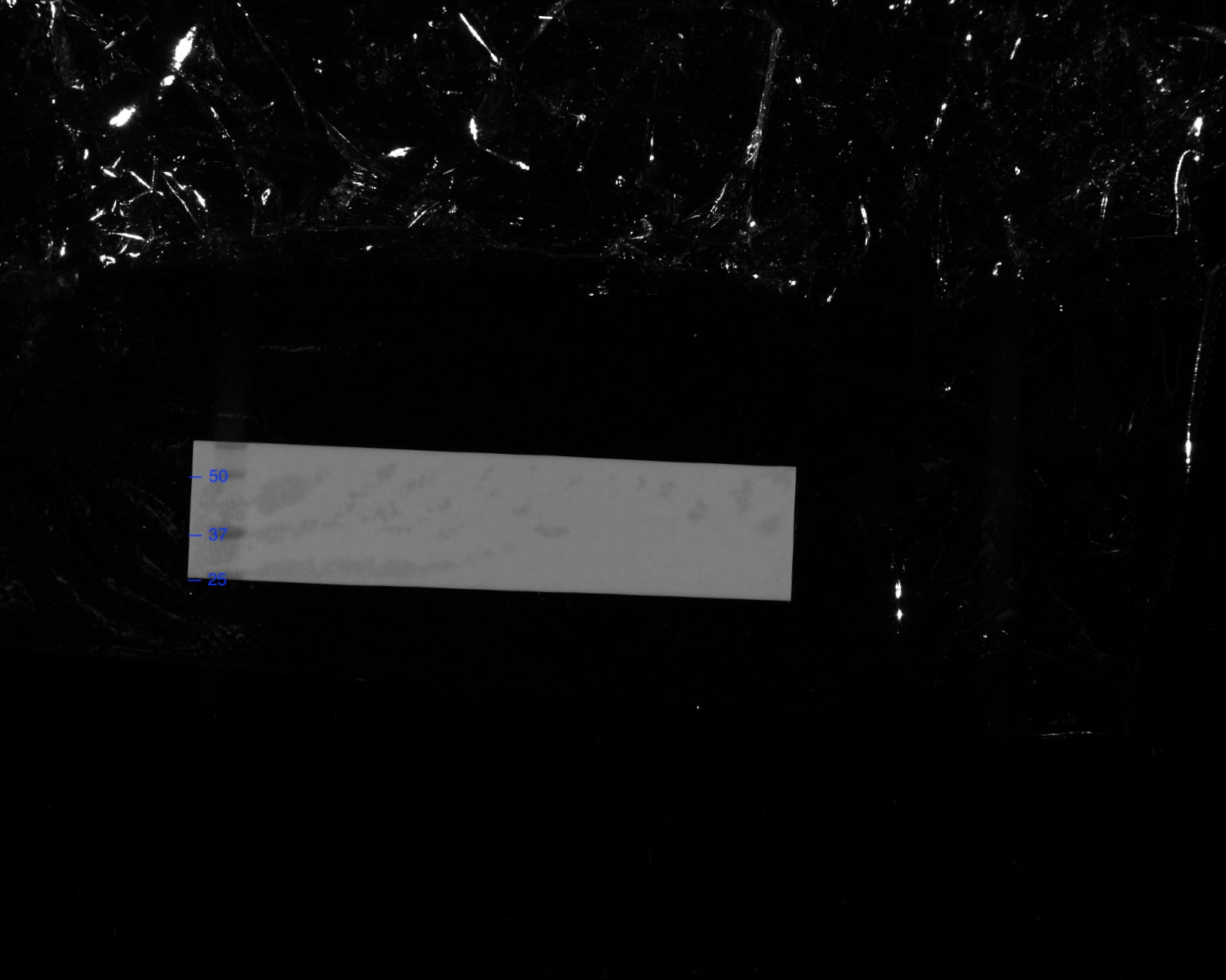

Supplement: Supplementary file 4 — Source data Fig. 1 [file 44319_2026_700_MOESM4_ESM.zip › Figure 1 -revised/1D - Western blot/NELFC(Ponceau S).jpg]

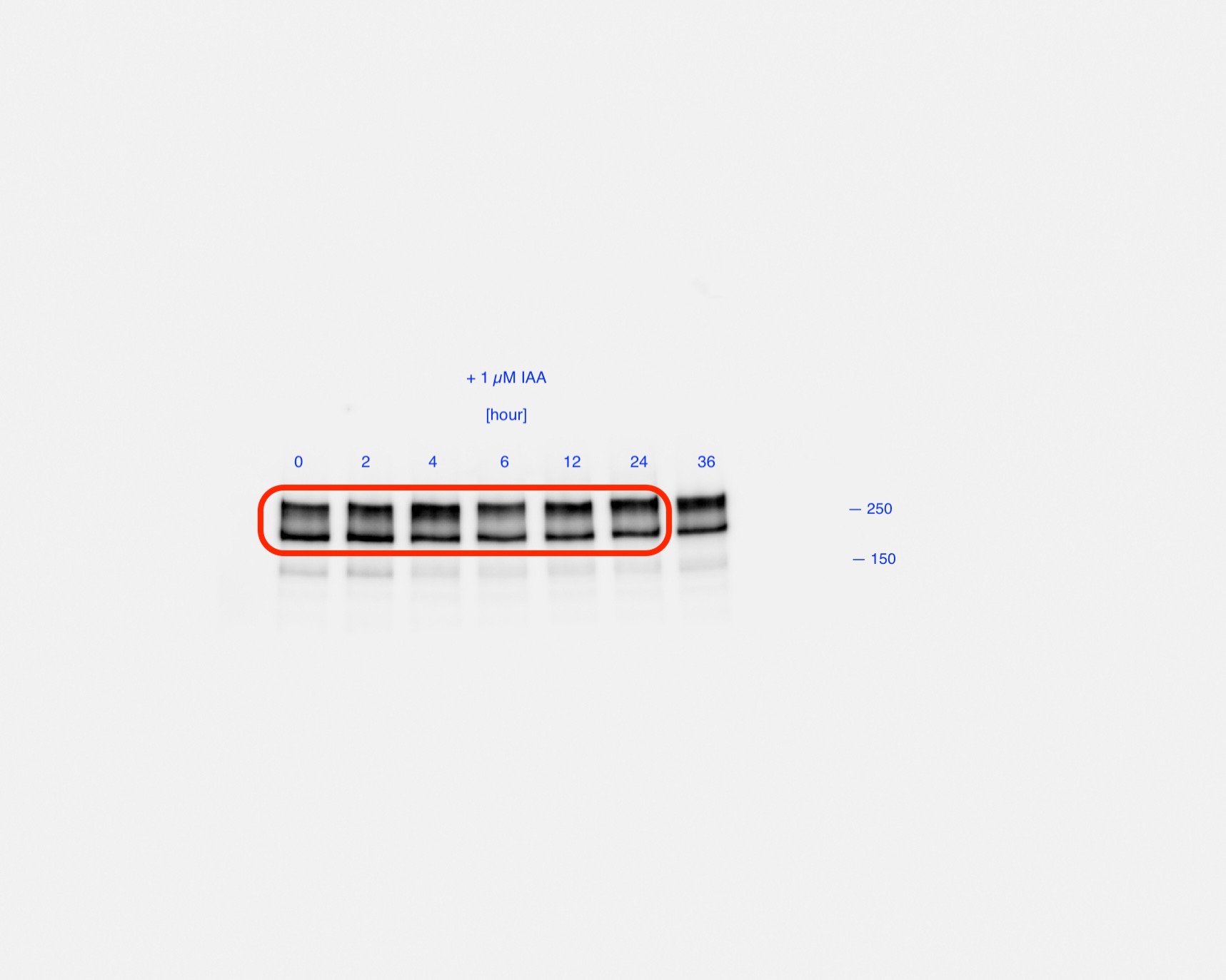

Supplement: Supplementary file 4 — Source data Fig. 1 [file 44319_2026_700_MOESM4_ESM.zip › Figure 1 -revised/1D - Western blot/Pol II(Chemiluminescence).jpg]

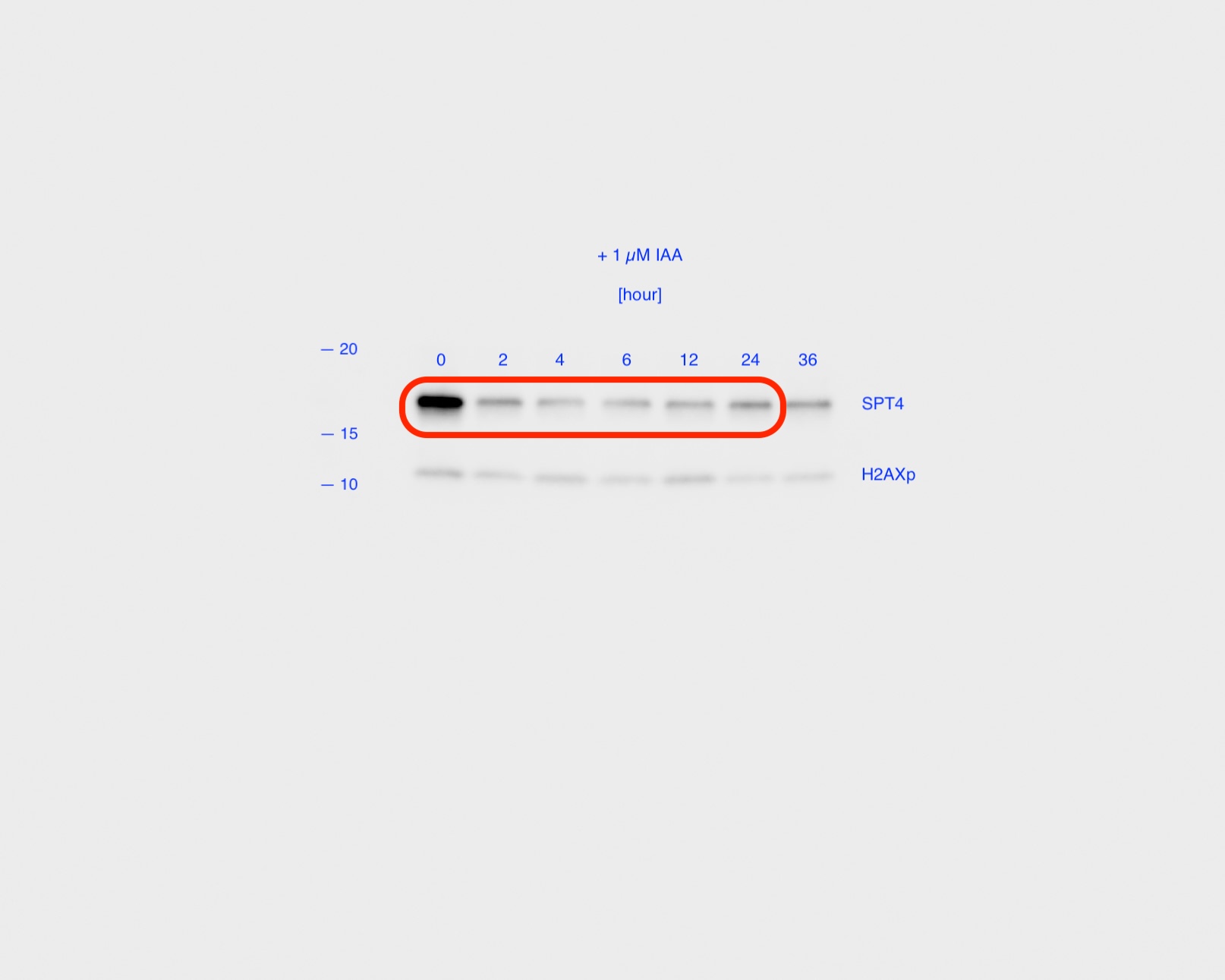

Supplement: Supplementary file 4 — Source data Fig. 1 [file 44319_2026_700_MOESM4_ESM.zip › Figure 1 -revised/1D - Western blot/SPT4 H2AXp(Chemiluminescence).jpg]

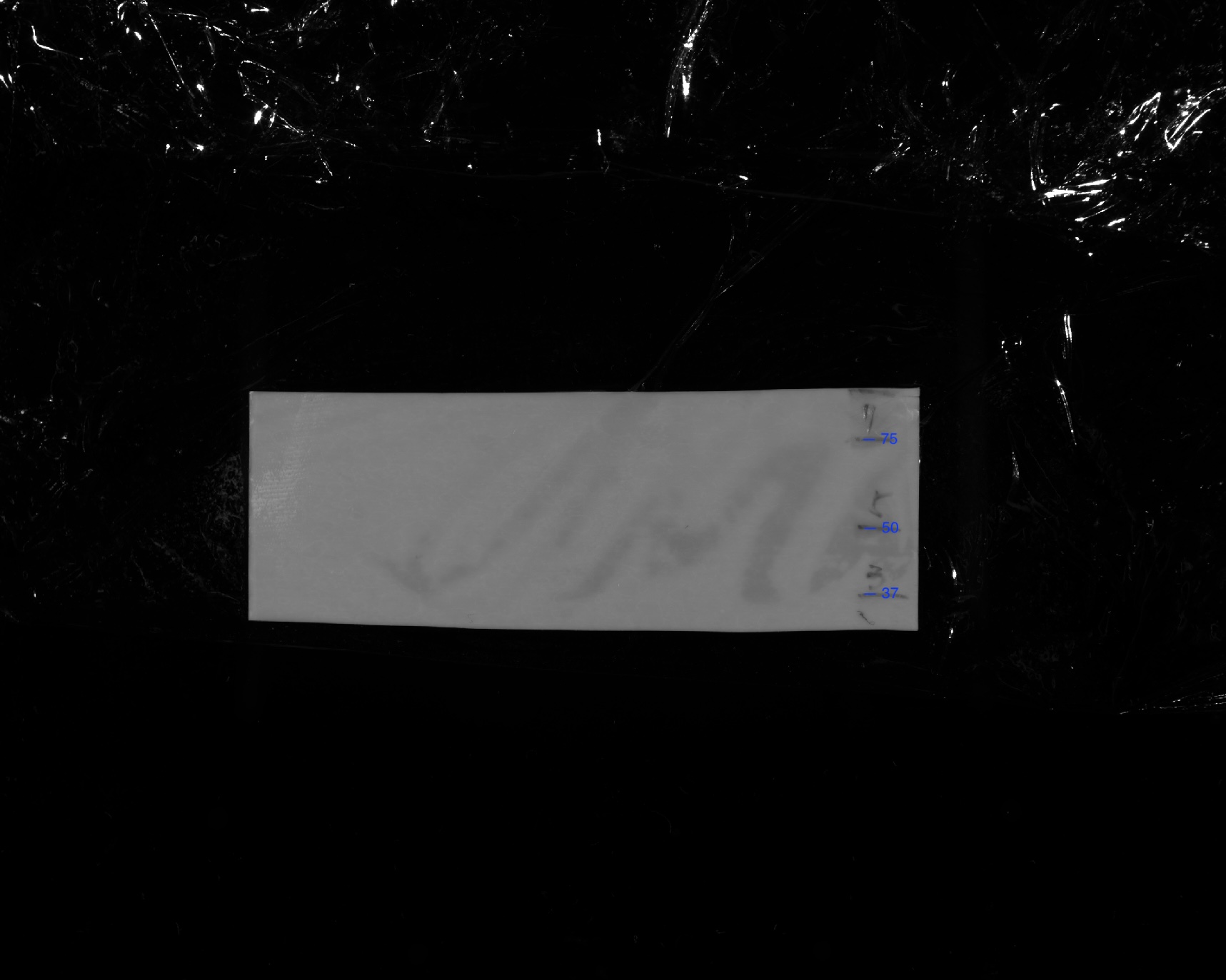

Supplement: Supplementary file 4 — Source data Fig. 1 [file 44319_2026_700_MOESM4_ESM.zip › Figure 1 -revised/1D - Western blot/NELFB NELFE(Ponceau S).jpg]

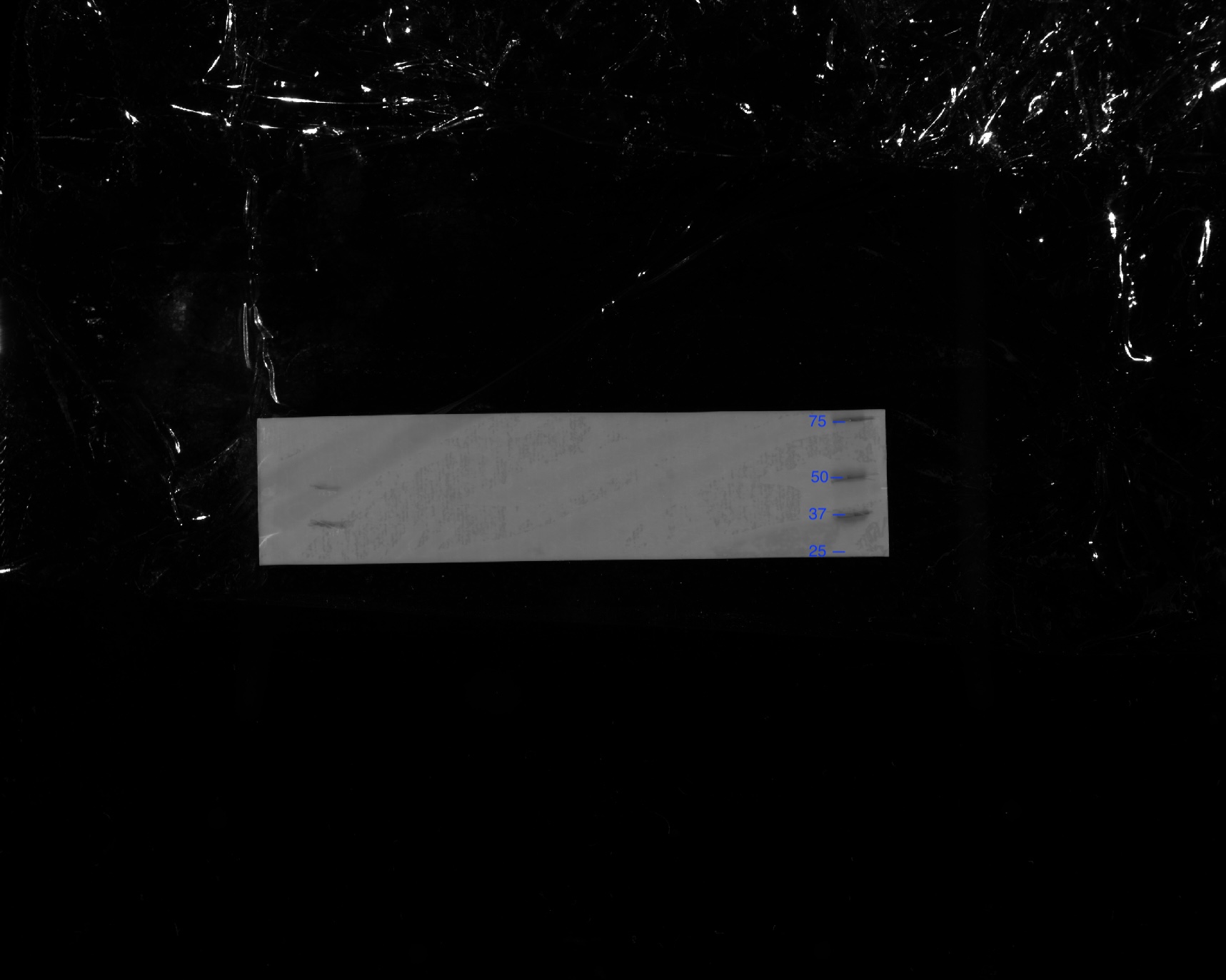

Supplement: Supplementary file 4 — Source data Fig. 1 [file 44319_2026_700_MOESM4_ESM.zip › Figure 1 -revised/1D - Western blot/NELFA(Ponceau S).jpg]

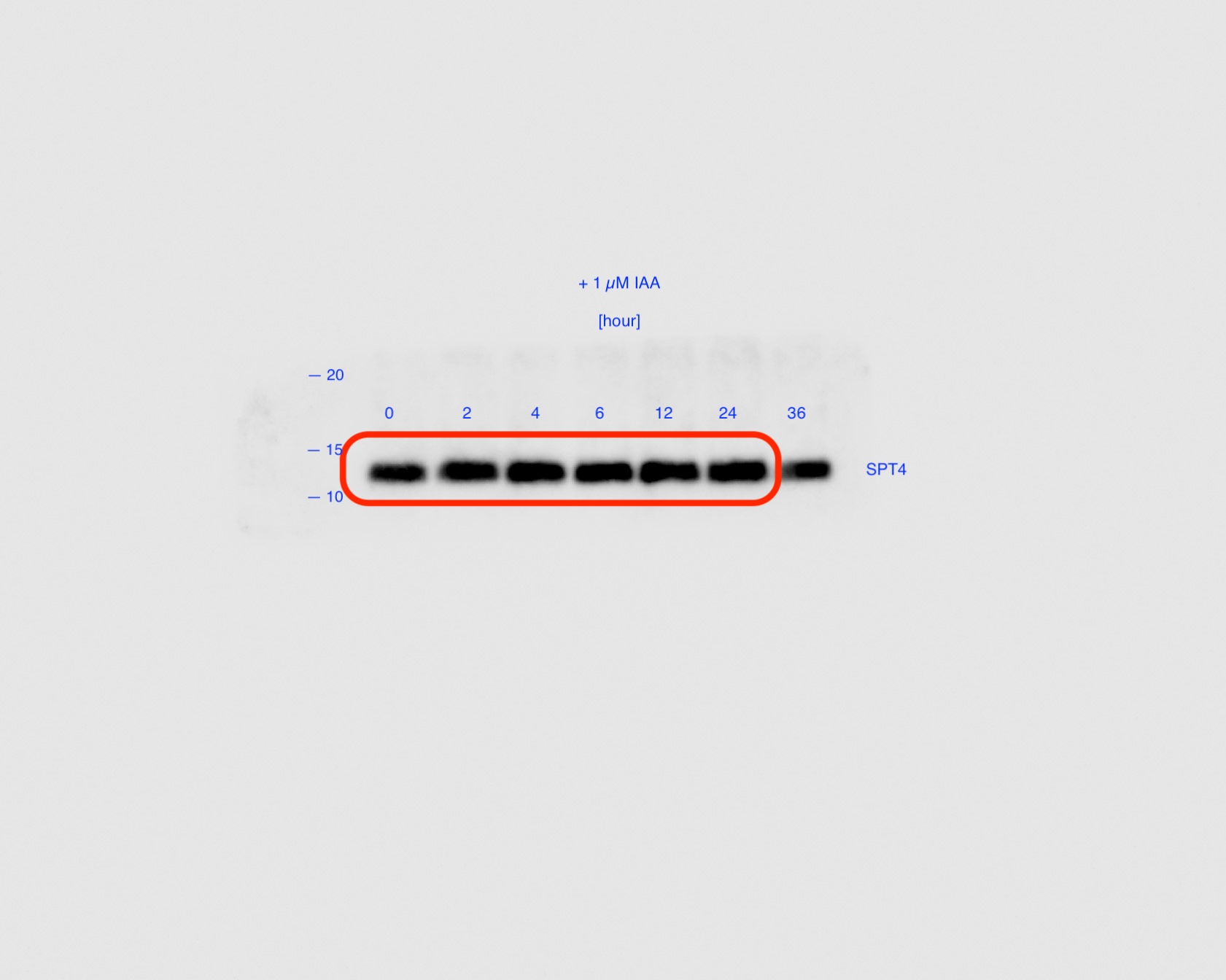

Supplement: Supplementary file 4 — Source data Fig. 1 [file 44319_2026_700_MOESM4_ESM.zip › Figure 1 -revised/1C - Western blot/SPT4(Chemiluminescence).jpg]

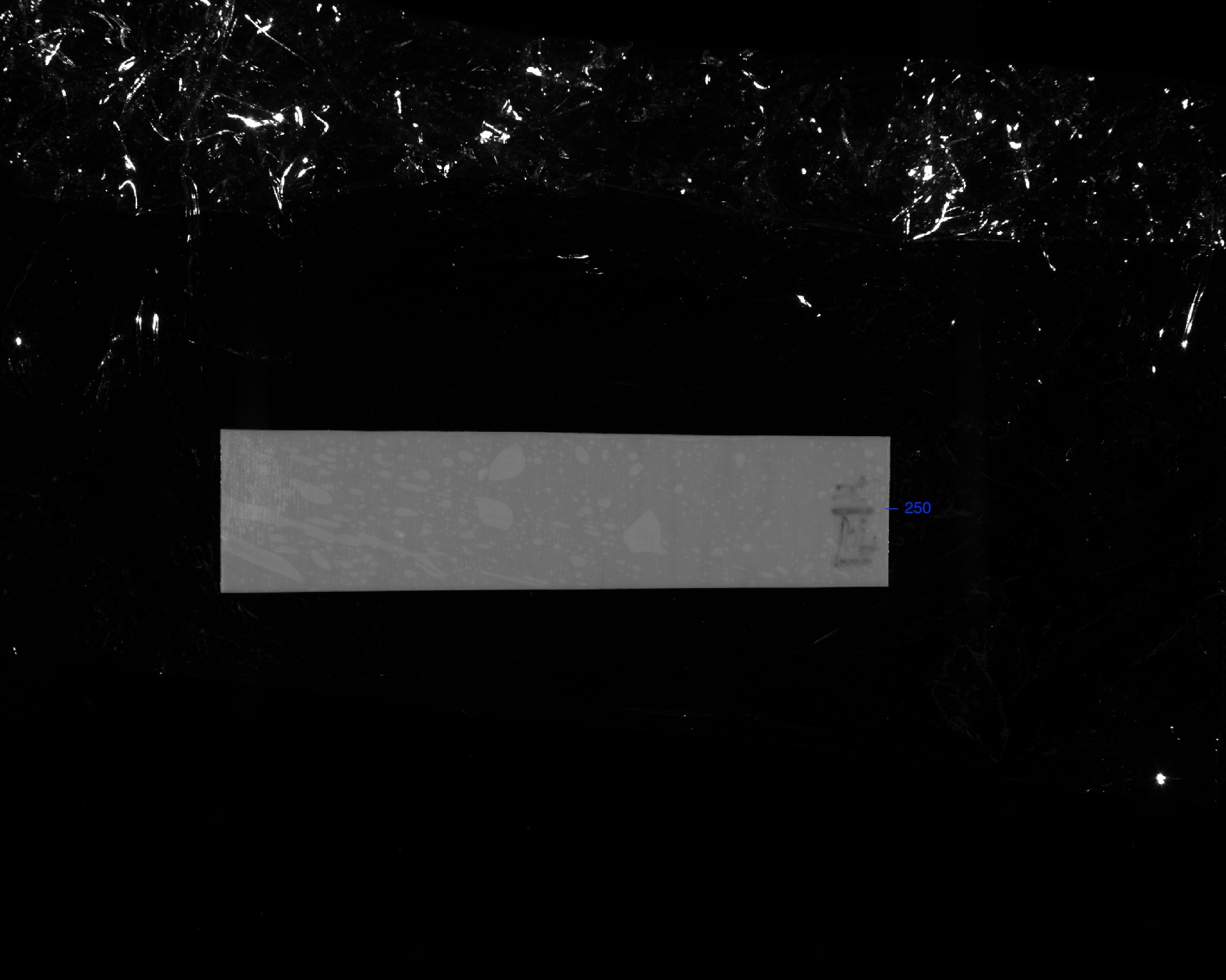

Supplement: Supplementary file 4 — Source data Fig. 1 [file 44319_2026_700_MOESM4_ESM.zip › Figure 1 -revised/1C - Western blot/Pol II(Ponceau S).jpg]

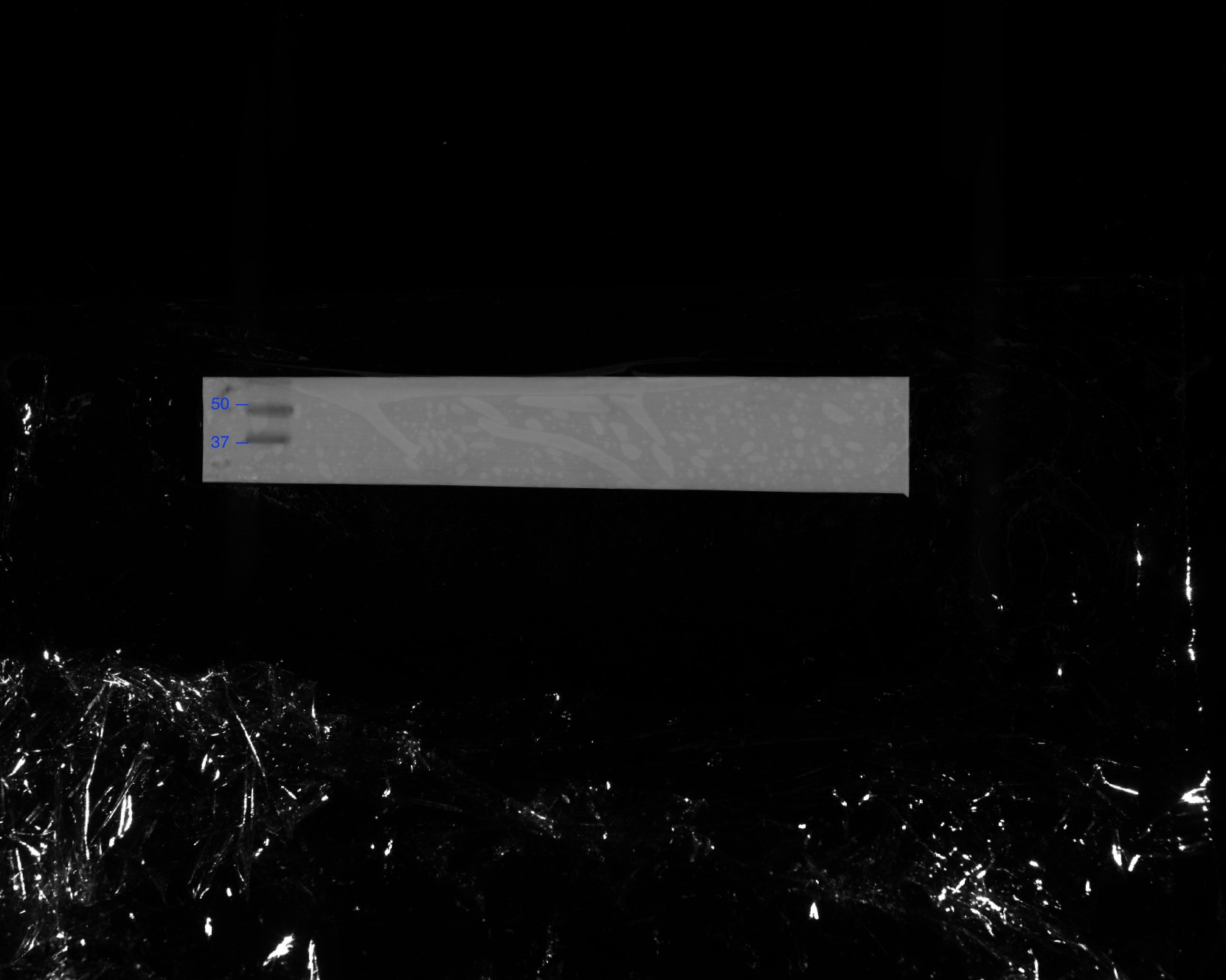

Supplement: Supplementary file 4 — Source data Fig. 1 [file 44319_2026_700_MOESM4_ESM.zip › Figure 1 -revised/1C - Western blot/TUB(Ponceau S).jpg]

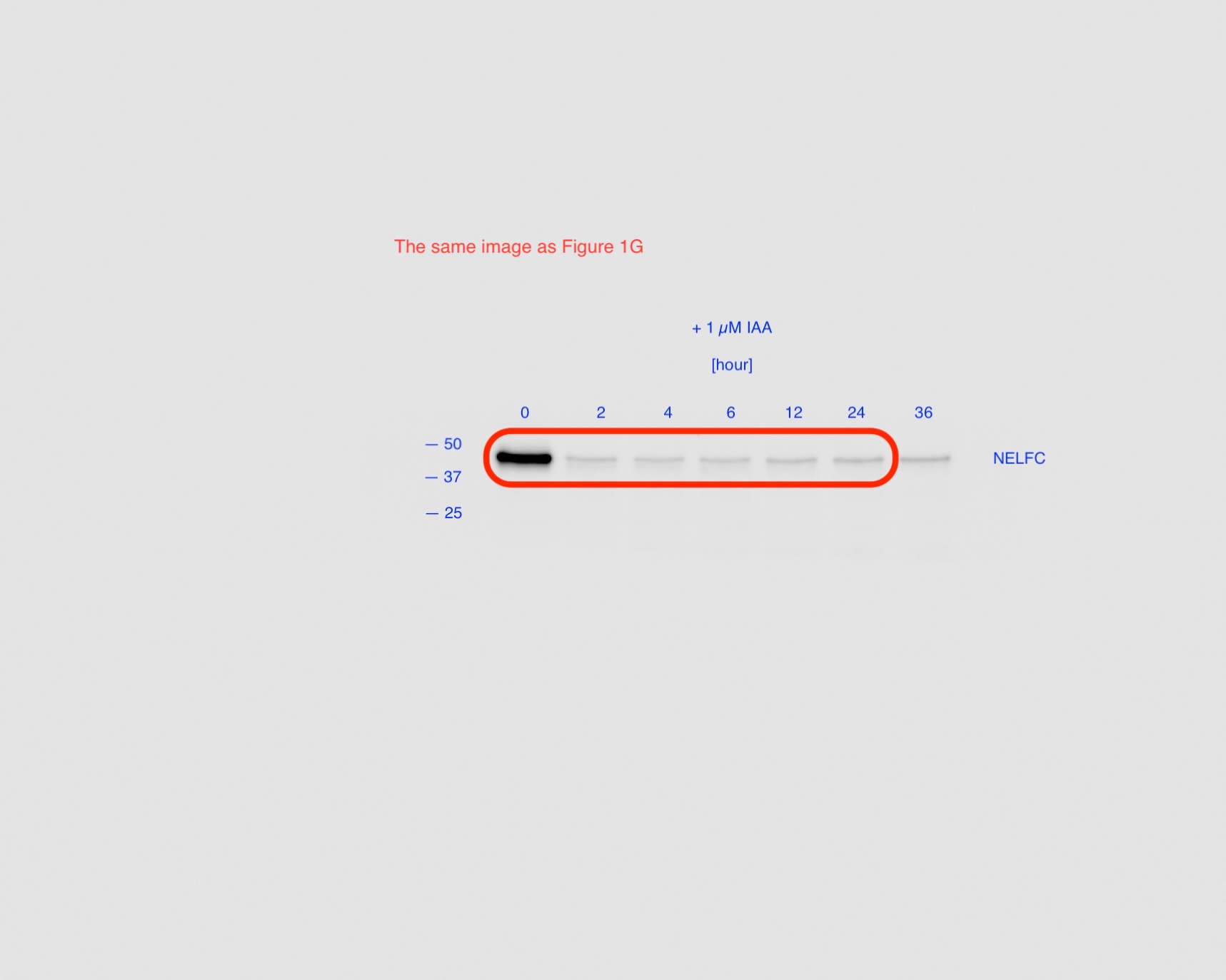

Supplement: Supplementary file 4 — Source data Fig. 1 [file 44319_2026_700_MOESM4_ESM.zip › Figure 1 -revised/1C - Western blot/NELFC(Chemiluminescence).jpg]

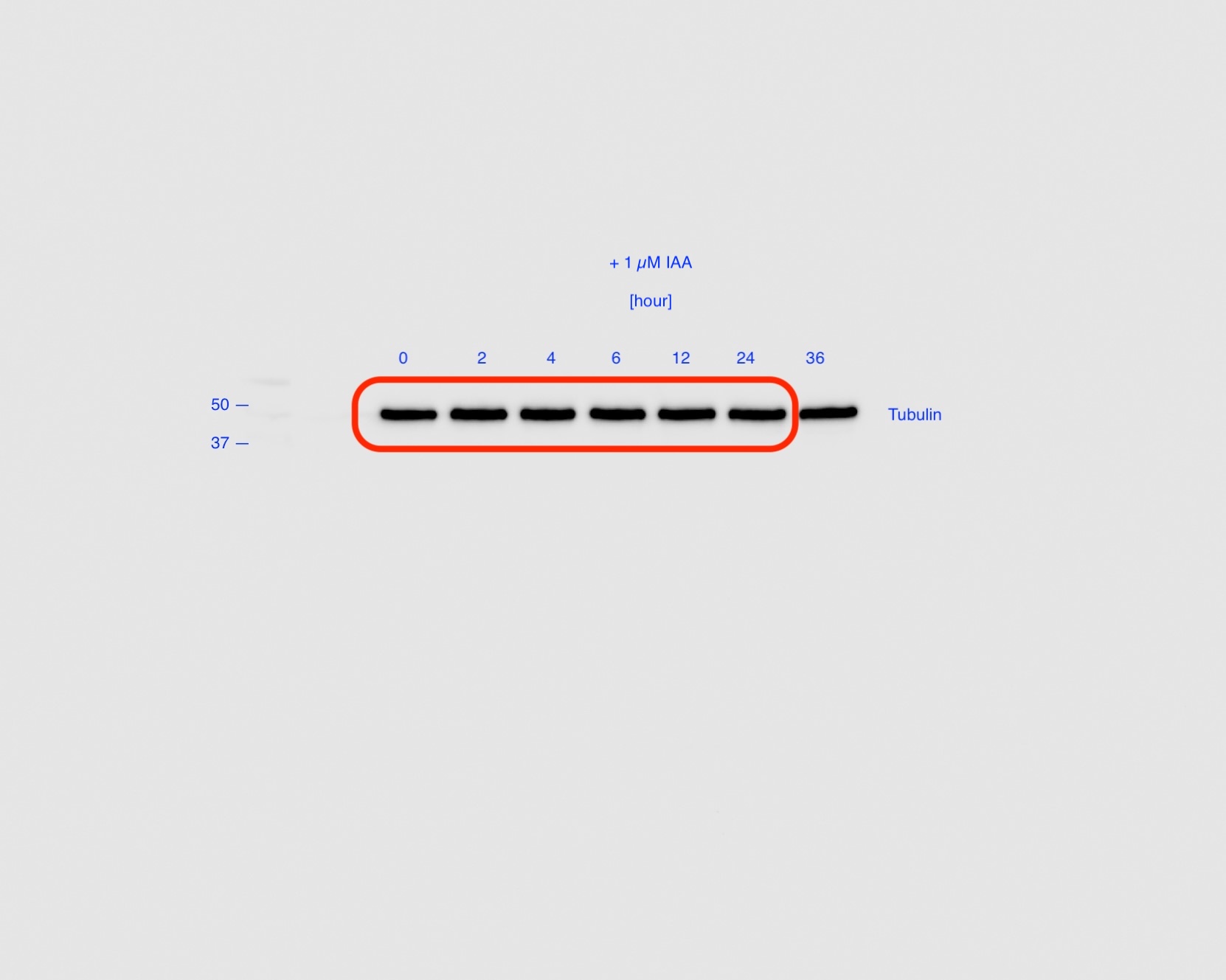

Supplement: Supplementary file 4 — Source data Fig. 1 [file 44319_2026_700_MOESM4_ESM.zip › Figure 1 -revised/1C - Western blot/TUB(Chemiluminescence).jpg]

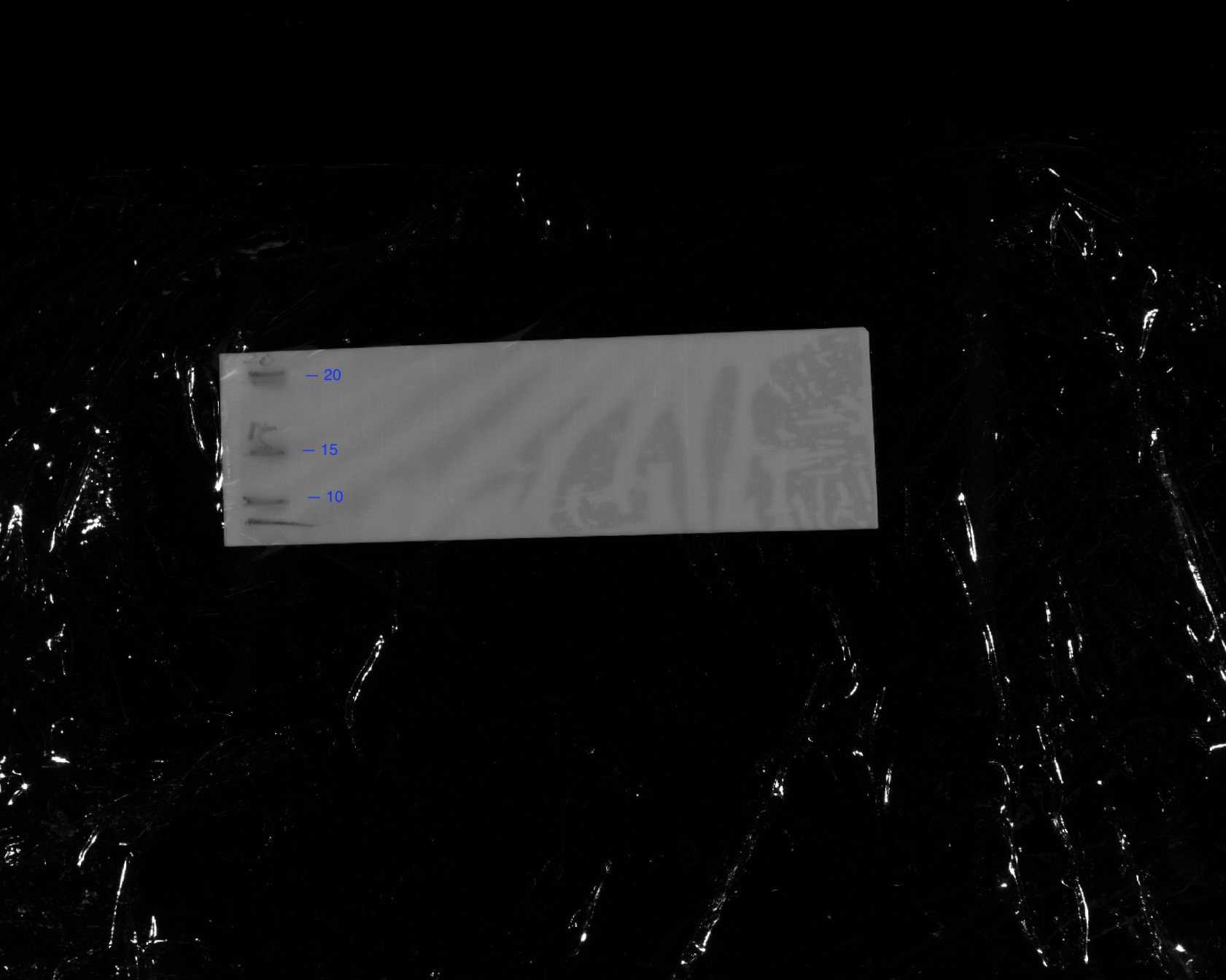

Supplement: Supplementary file 4 — Source data Fig. 1 [file 44319_2026_700_MOESM4_ESM.zip › Figure 1 -revised/1C - Western blot/SPT4(Ponceau S).jpg]

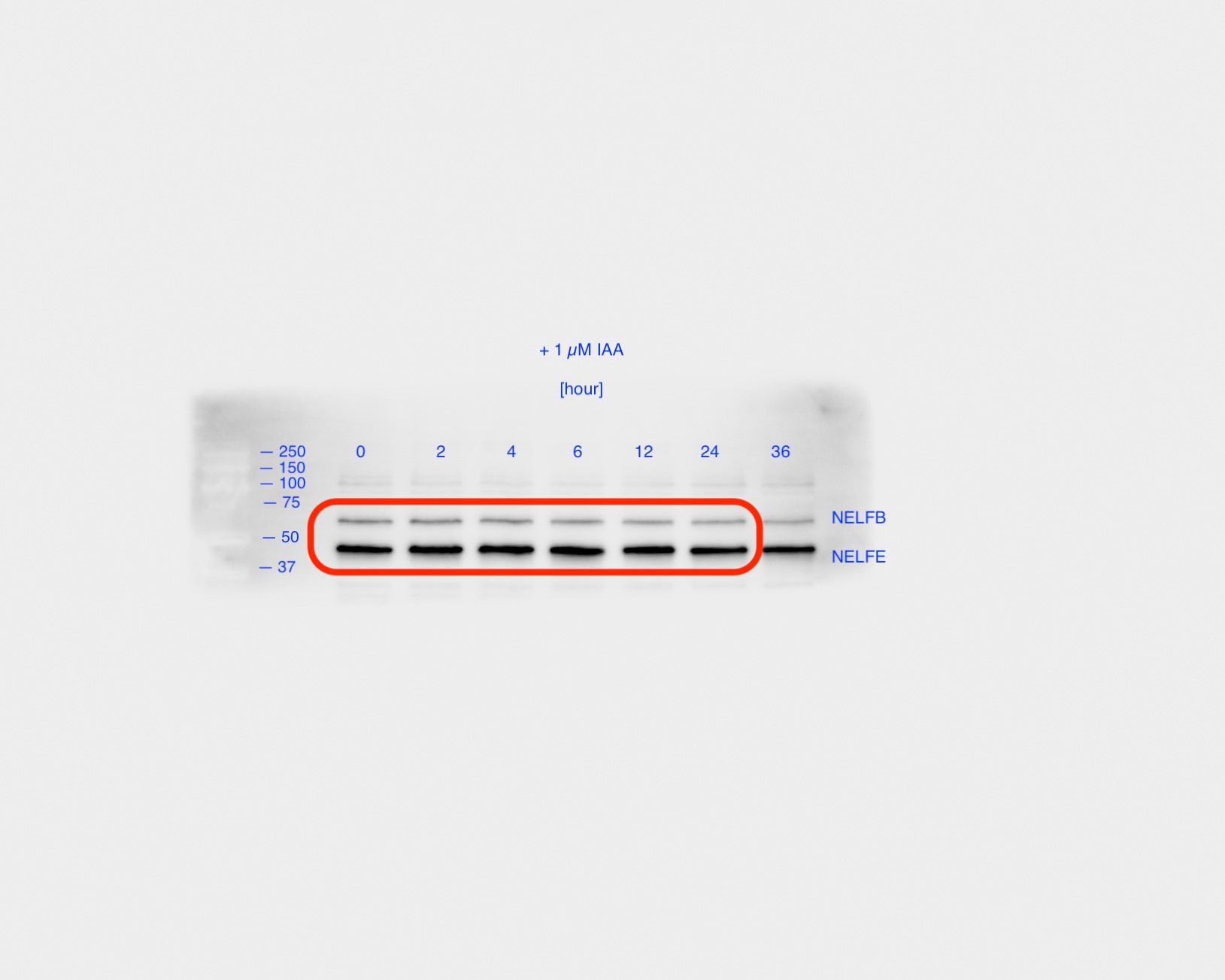

Supplement: Supplementary file 4 — Source data Fig. 1 [file 44319_2026_700_MOESM4_ESM.zip › Figure 1 -revised/1C - Western blot/NELFB NELFE(Chemiluminescence).jpg]

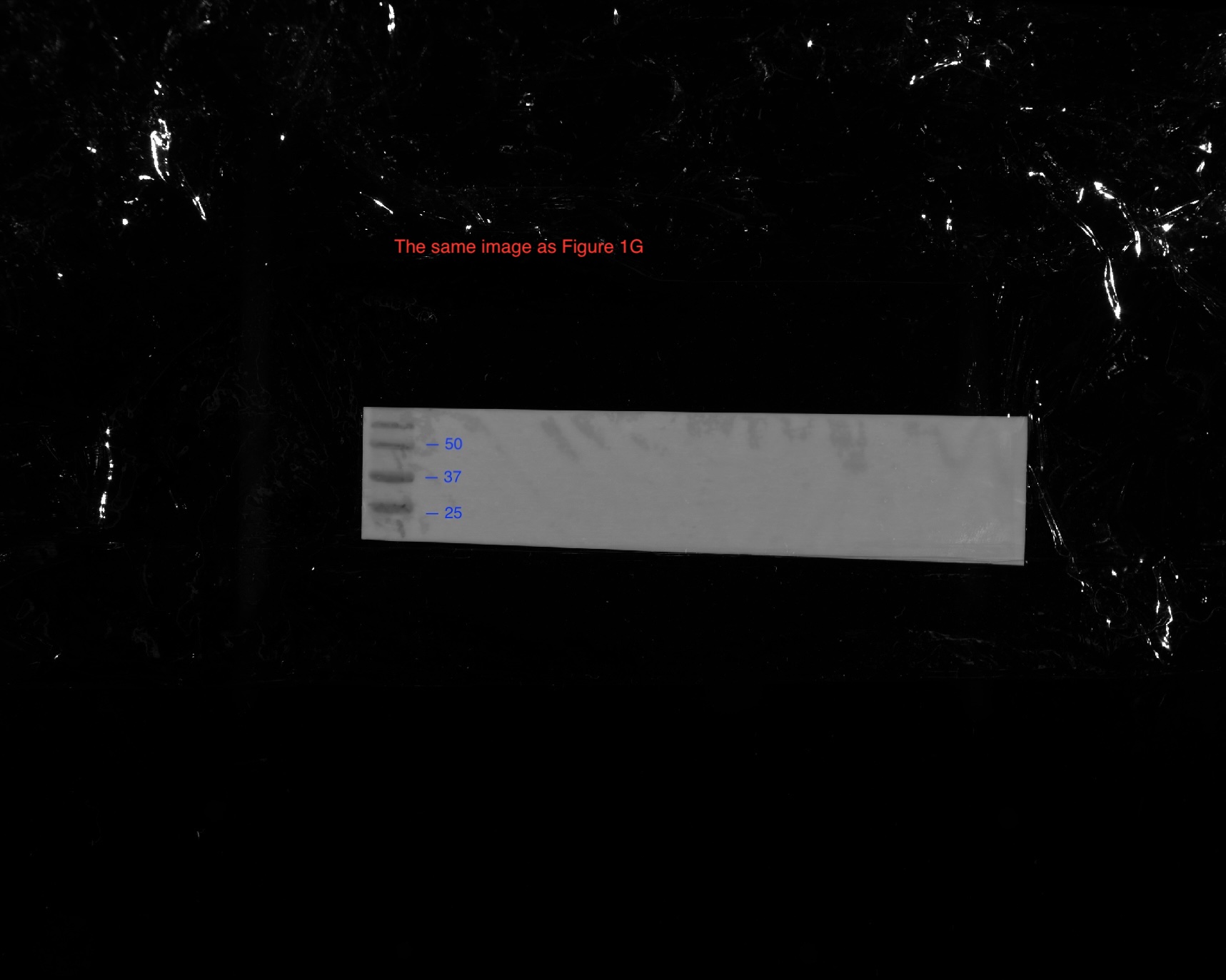

Supplement: Supplementary file 4 — Source data Fig. 1 [file 44319_2026_700_MOESM4_ESM.zip › Figure 1 -revised/1C - Western blot/NELFC(Ponceau S).jpg]

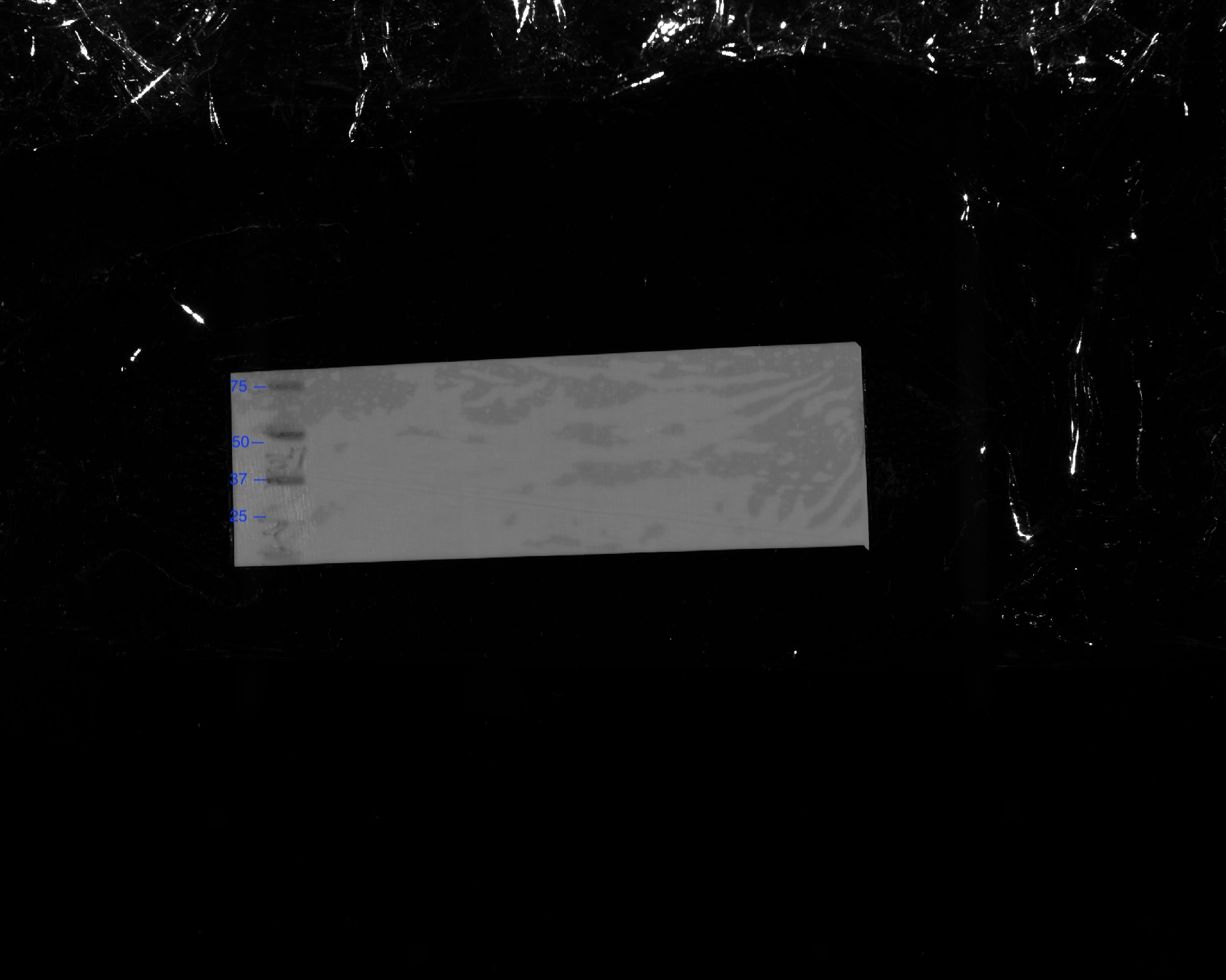

Supplement: Supplementary file 4 — Source data Fig. 1 [file 44319_2026_700_MOESM4_ESM.zip › Figure 1 -revised/1C - Western blot/NELFA_JUNB(Ponceau S).jpg]

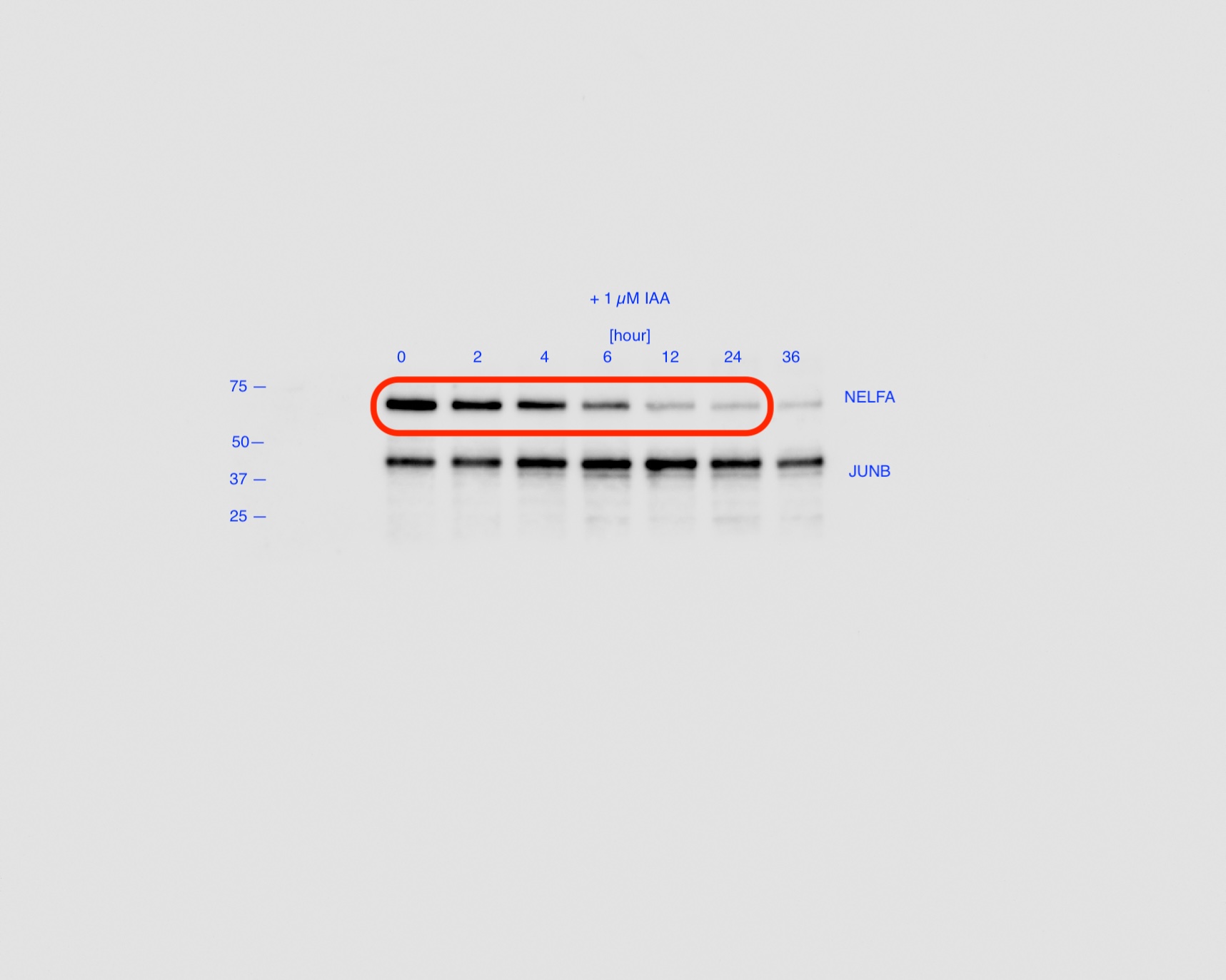

Supplement: Supplementary file 4 — Source data Fig. 1 [file 44319_2026_700_MOESM4_ESM.zip › Figure 1 -revised/1C - Western blot/NELFA_JUNB(Chemiluminescence).jpg]

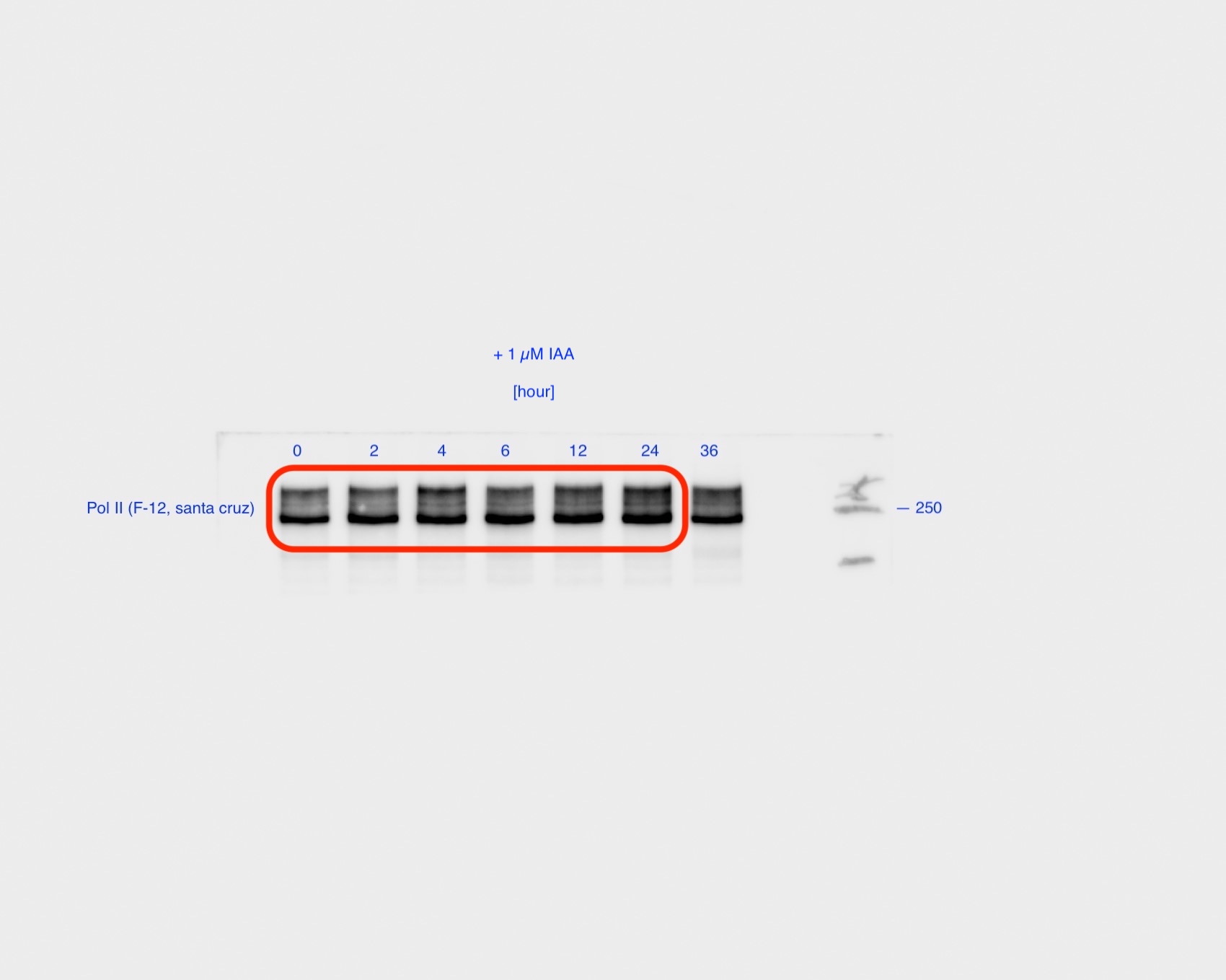

Supplement: Supplementary file 4 — Source data Fig. 1 [file 44319_2026_700_MOESM4_ESM.zip › Figure 1 -revised/1C - Western blot/Pol II(Chemiluminescence).jpg]

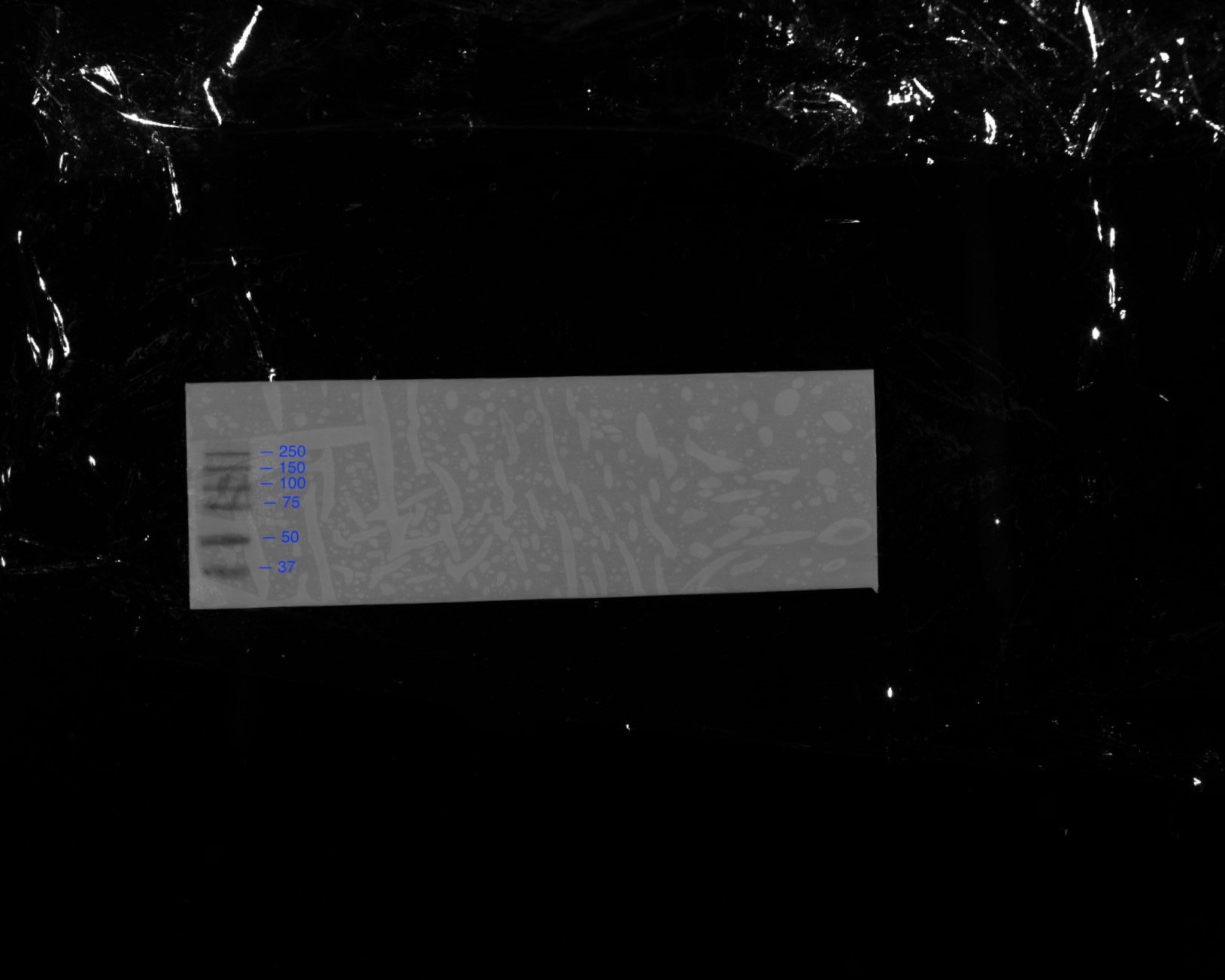

Supplement: Supplementary file 4 — Source data Fig. 1 [file 44319_2026_700_MOESM4_ESM.zip › Figure 1 -revised/1C - Western blot/NELFB NELFE(Ponceau S).jpg]

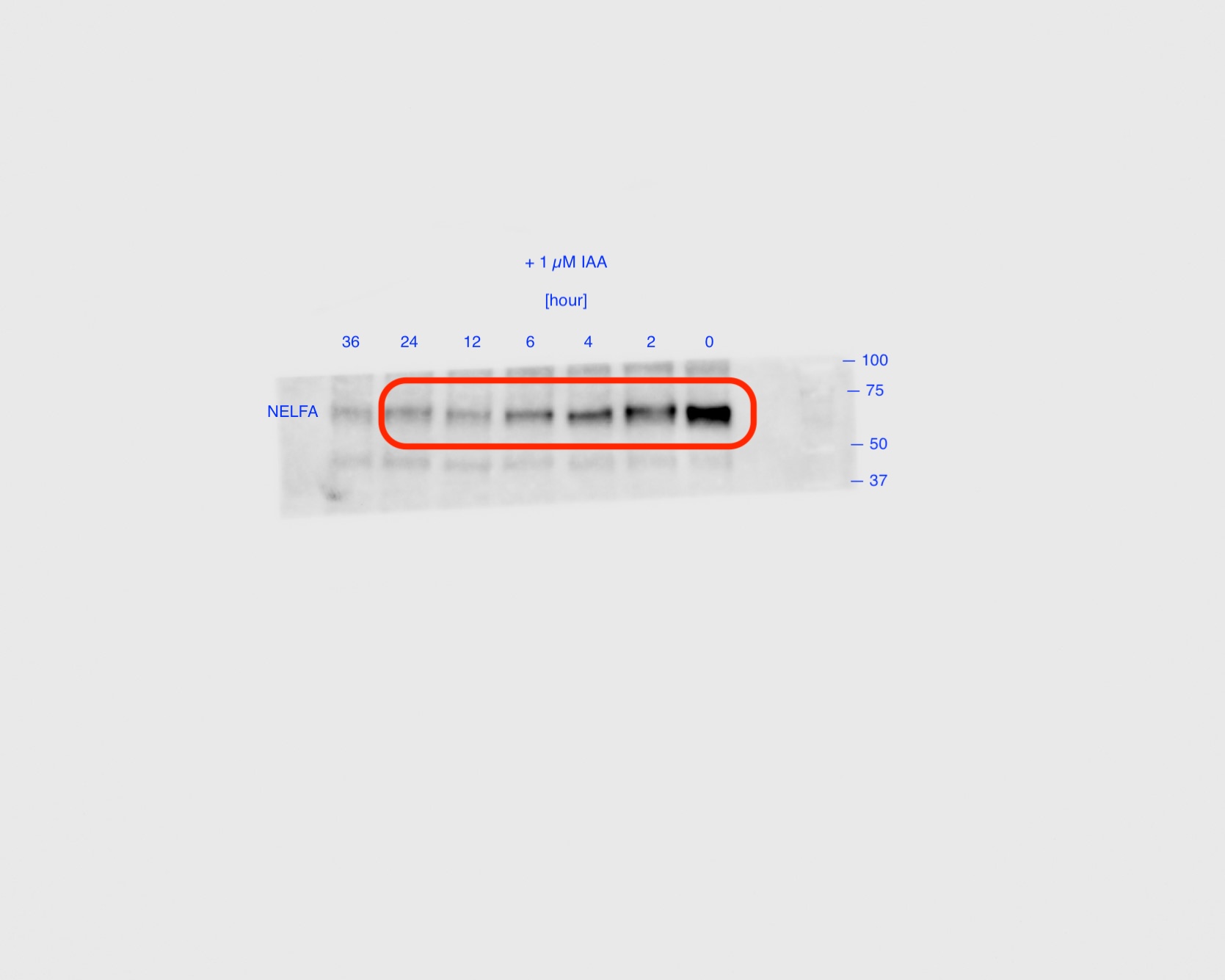

Supplement: Supplementary file 4 — Source data Fig. 1 [file 44319_2026_700_MOESM4_ESM.zip › Figure 1 -revised/1E - Western blot/NELFA(Chemiluminescence).jpg]

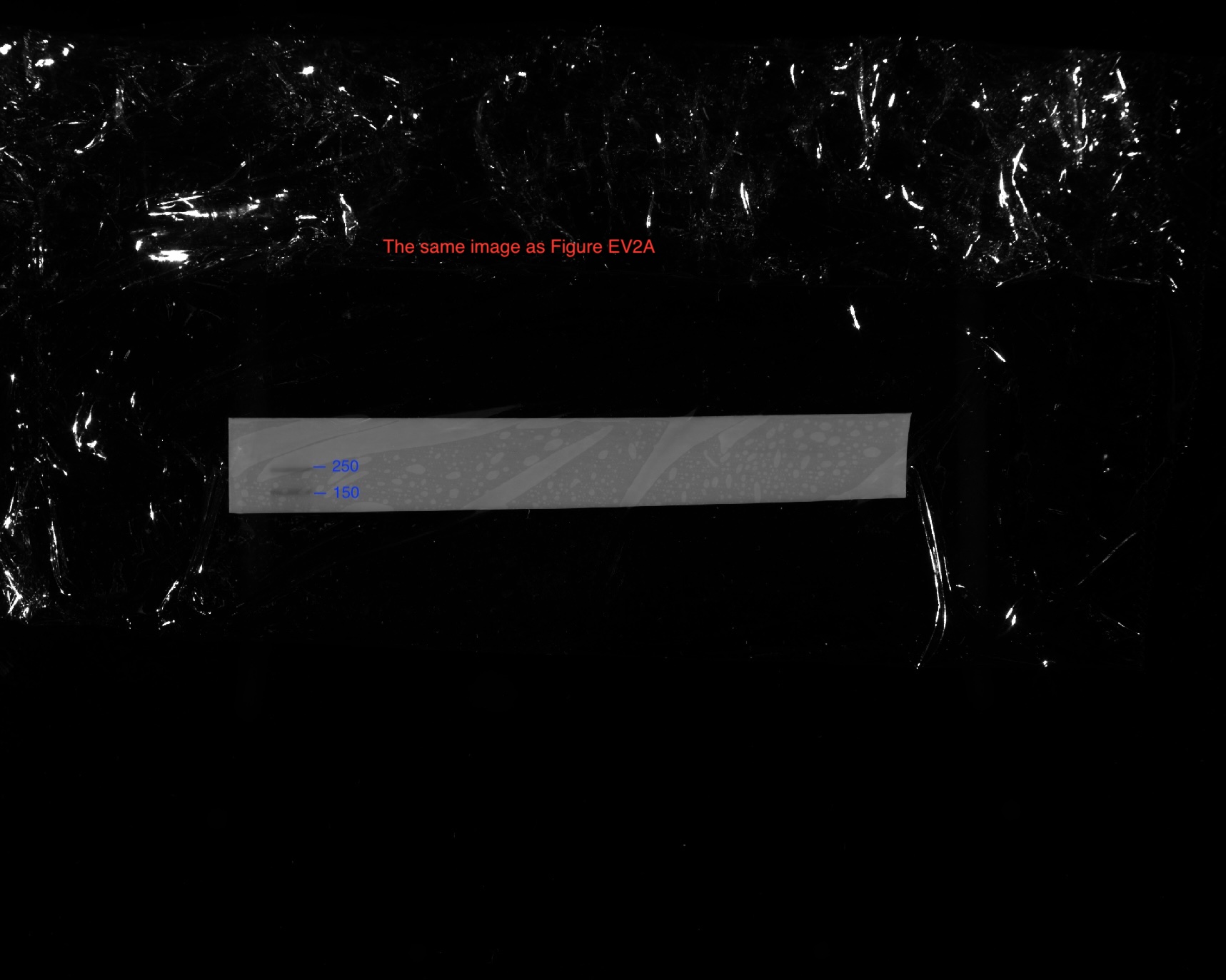

Supplement: Supplementary file 4 — Source data Fig. 1 [file 44319_2026_700_MOESM4_ESM.zip › Figure 1 -revised/1E - Western blot/Pol I_RPA196(Ponceau S).jpg]

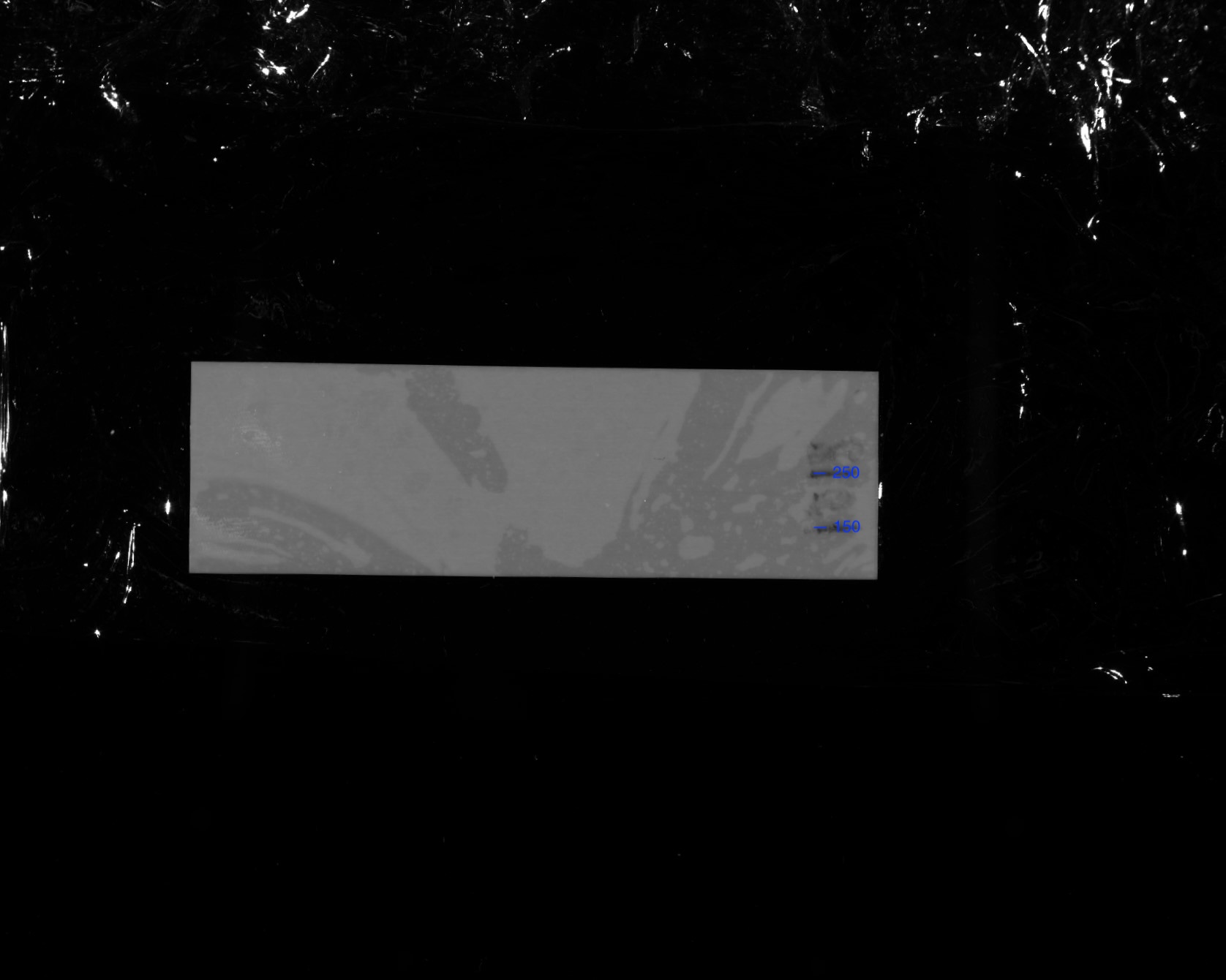

Supplement: Supplementary file 4 — Source data Fig. 1 [file 44319_2026_700_MOESM4_ESM.zip › Figure 1 -revised/1E - Western blot/Pol II(Ponceau S).jpg]

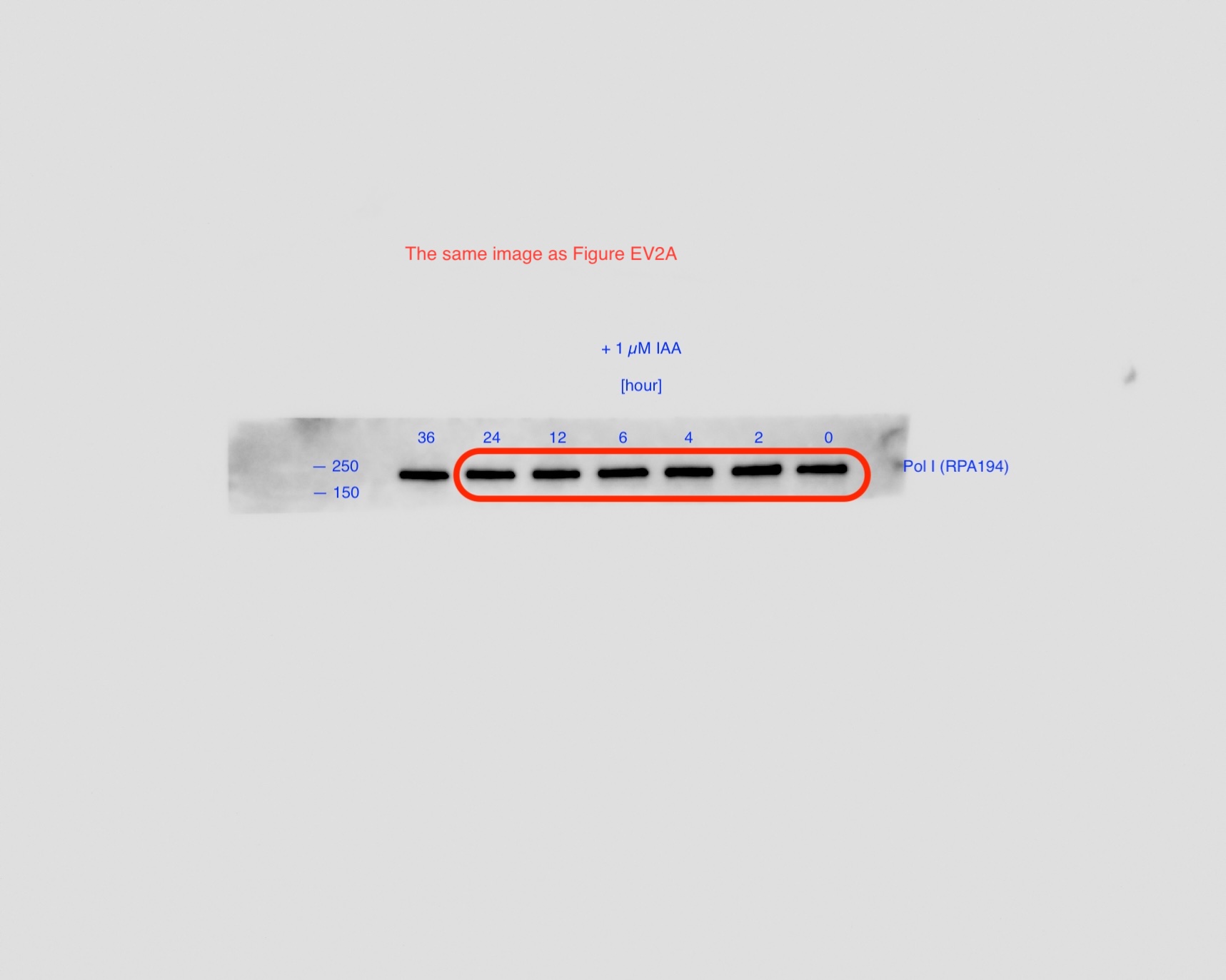

Supplement: Supplementary file 4 — Source data Fig. 1 [file 44319_2026_700_MOESM4_ESM.zip › Figure 1 -revised/1E - Western blot/Pol I_RPA196(Chemiluminescence).jpg]

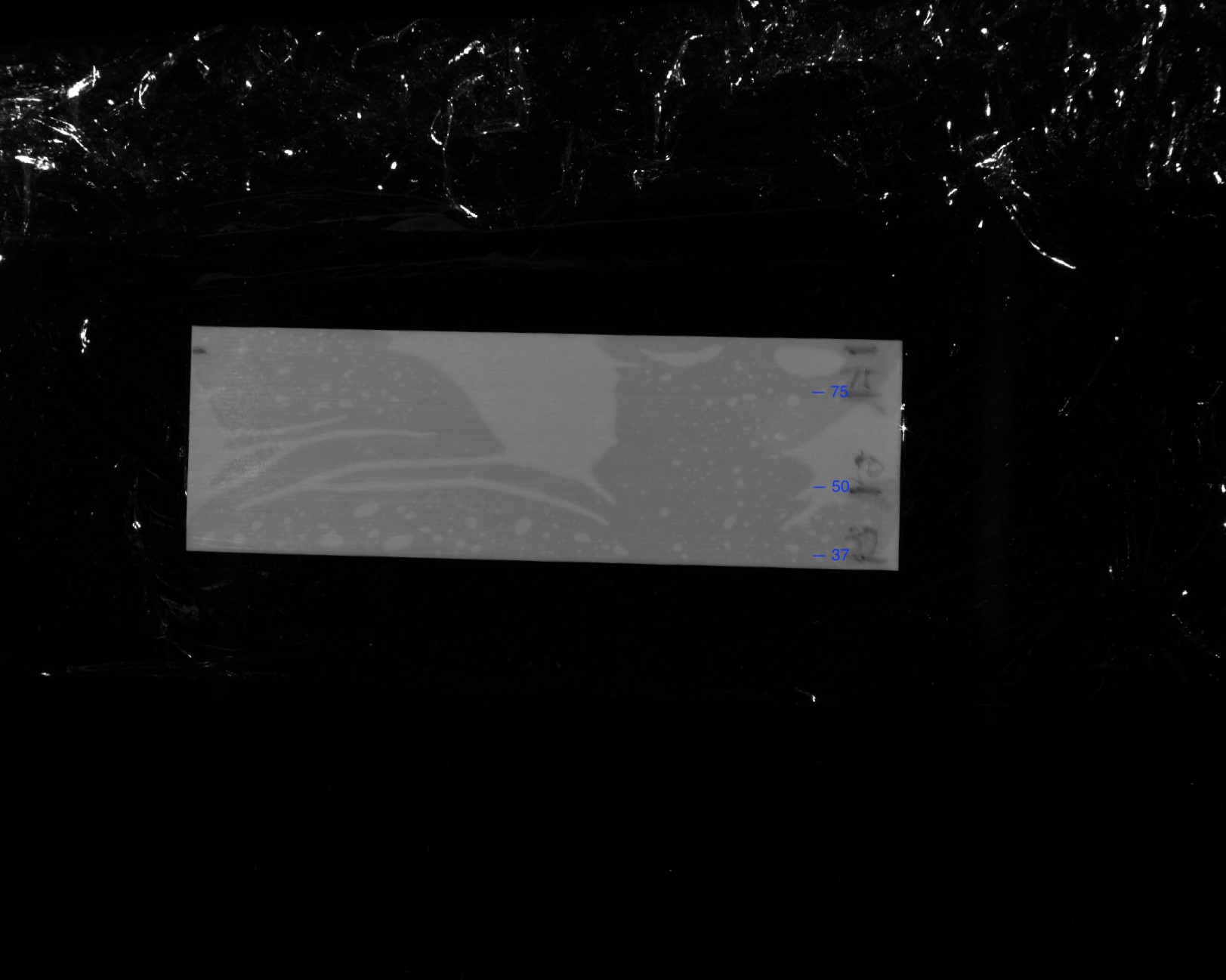

Supplement: Supplementary file 4 — Source data Fig. 1 [file 44319_2026_700_MOESM4_ESM.zip › Figure 1 -revised/1E - Western blot/NELFB_E(Ponceau S).jpg]

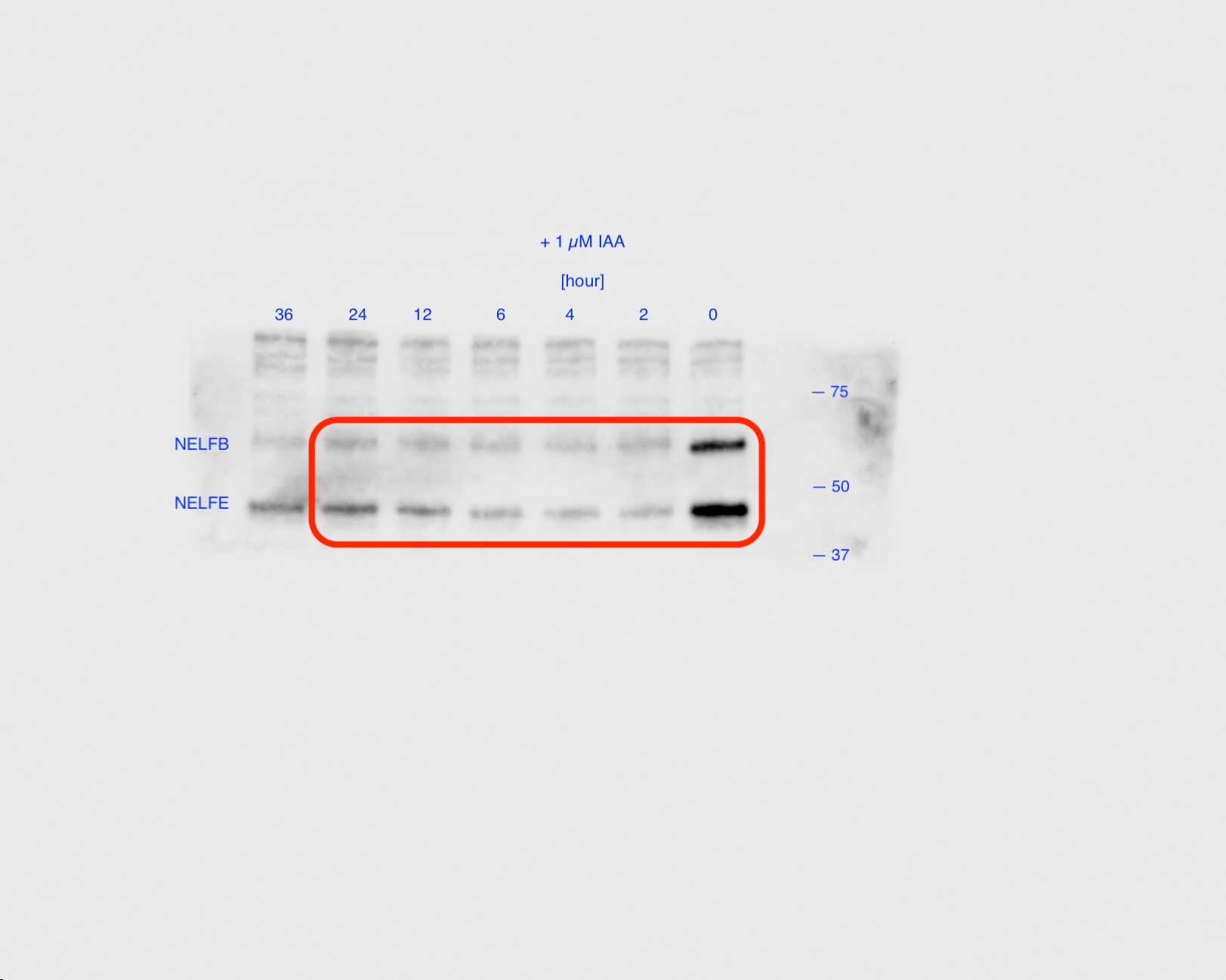

Supplement: Supplementary file 4 — Source data Fig. 1 [file 44319_2026_700_MOESM4_ESM.zip › Figure 1 -revised/1E - Western blot/NELFB_E(Chemiluminescence).jpg]

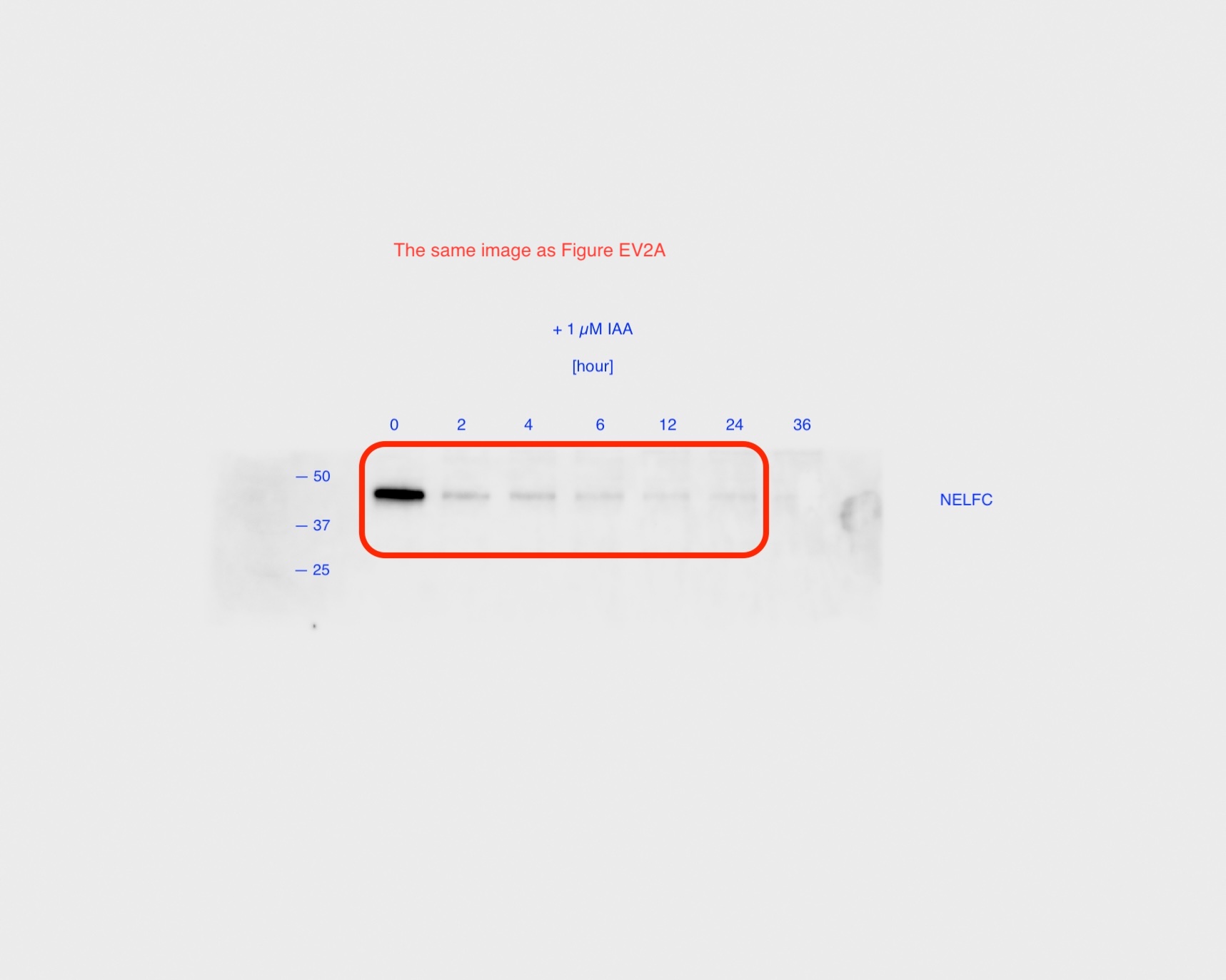

Supplement: Supplementary file 4 — Source data Fig. 1 [file 44319_2026_700_MOESM4_ESM.zip › Figure 1 -revised/1E - Western blot/NELFC(Chemiluminescence).jpg]

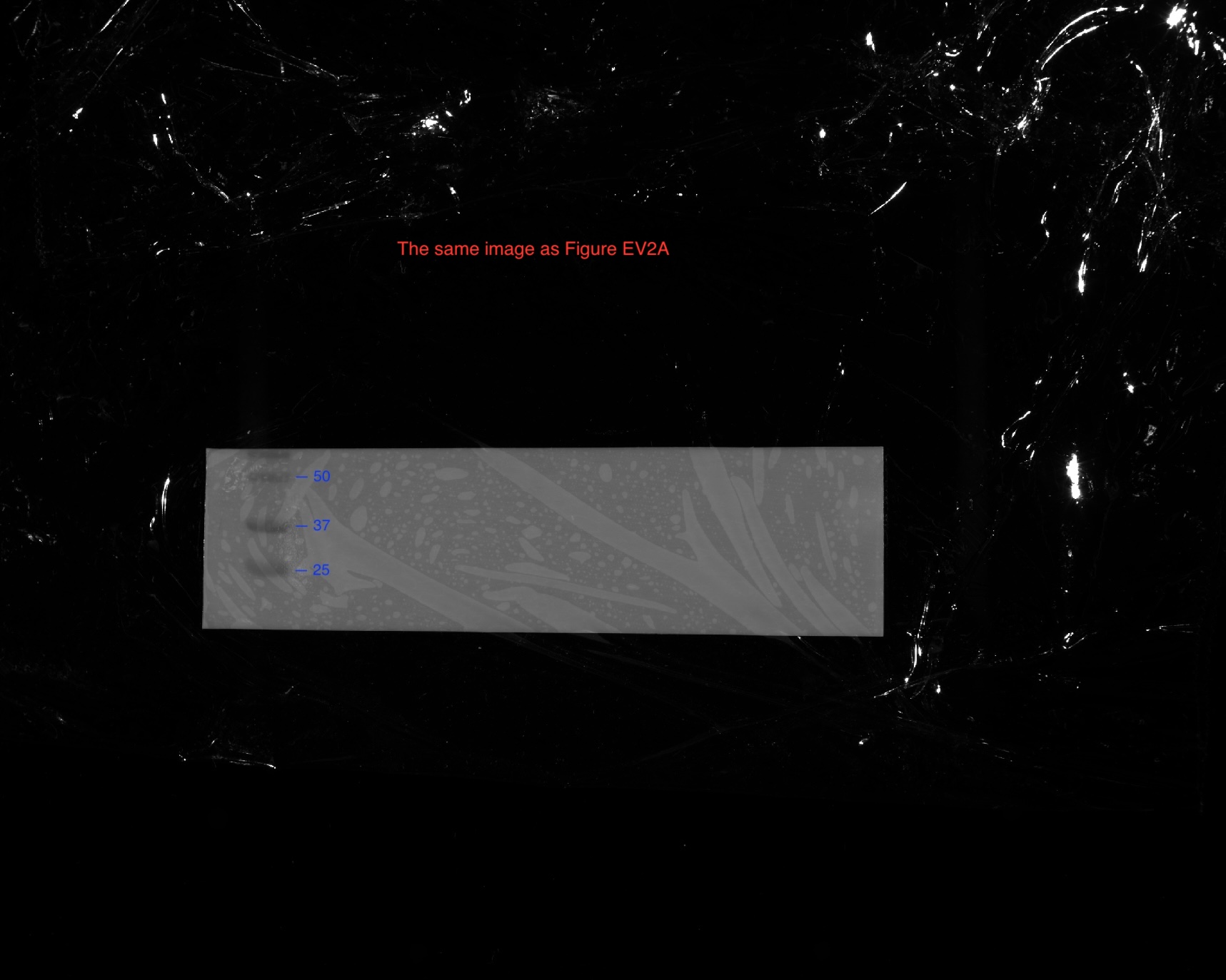

Supplement: Supplementary file 4 — Source data Fig. 1 [file 44319_2026_700_MOESM4_ESM.zip › Figure 1 -revised/1E - Western blot/NELFC(Ponceau S).jpg]

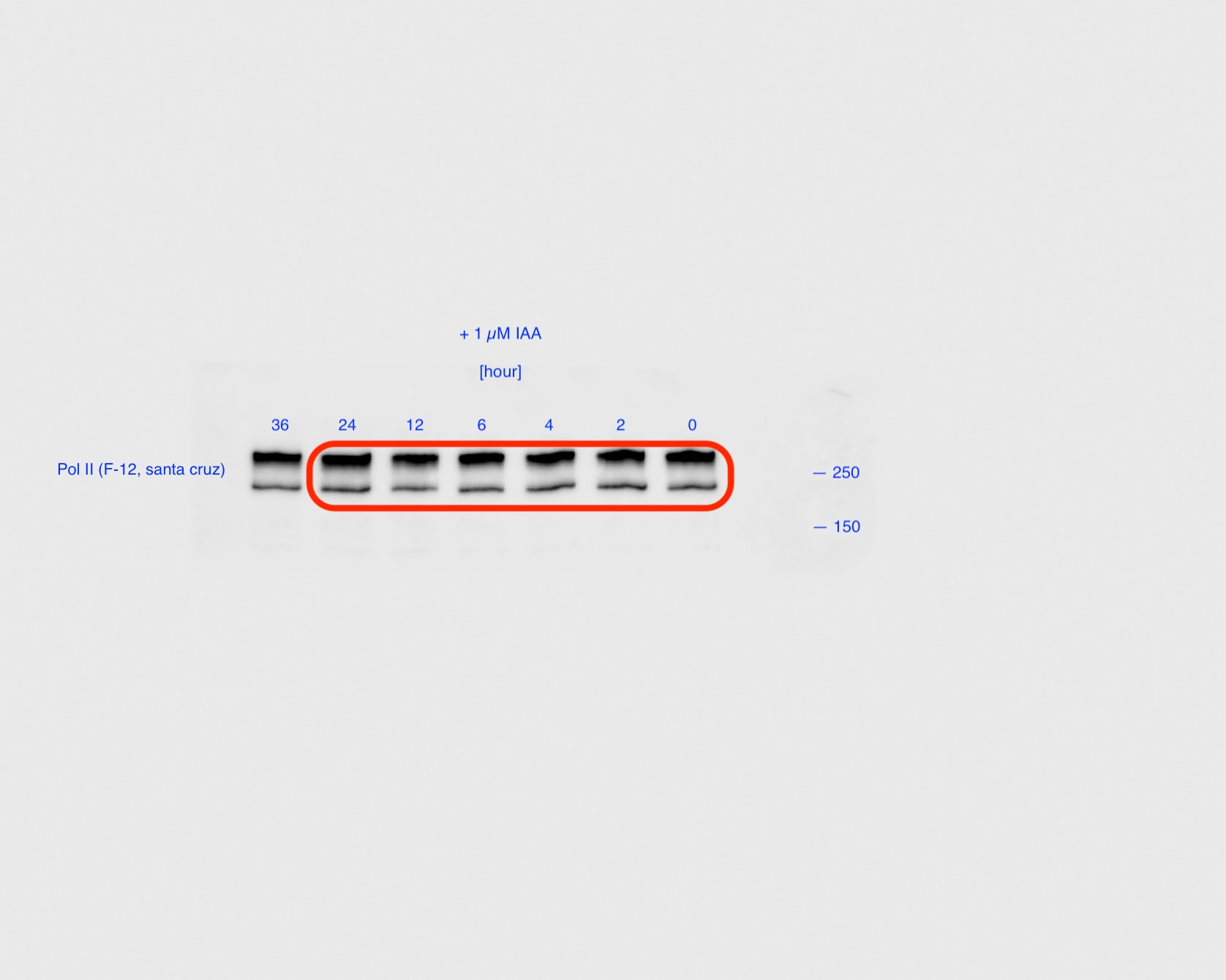

Supplement: Supplementary file 4 — Source data Fig. 1 [file 44319_2026_700_MOESM4_ESM.zip › Figure 1 -revised/1E - Western blot/Pol II(Chemiluminescence).jpg]

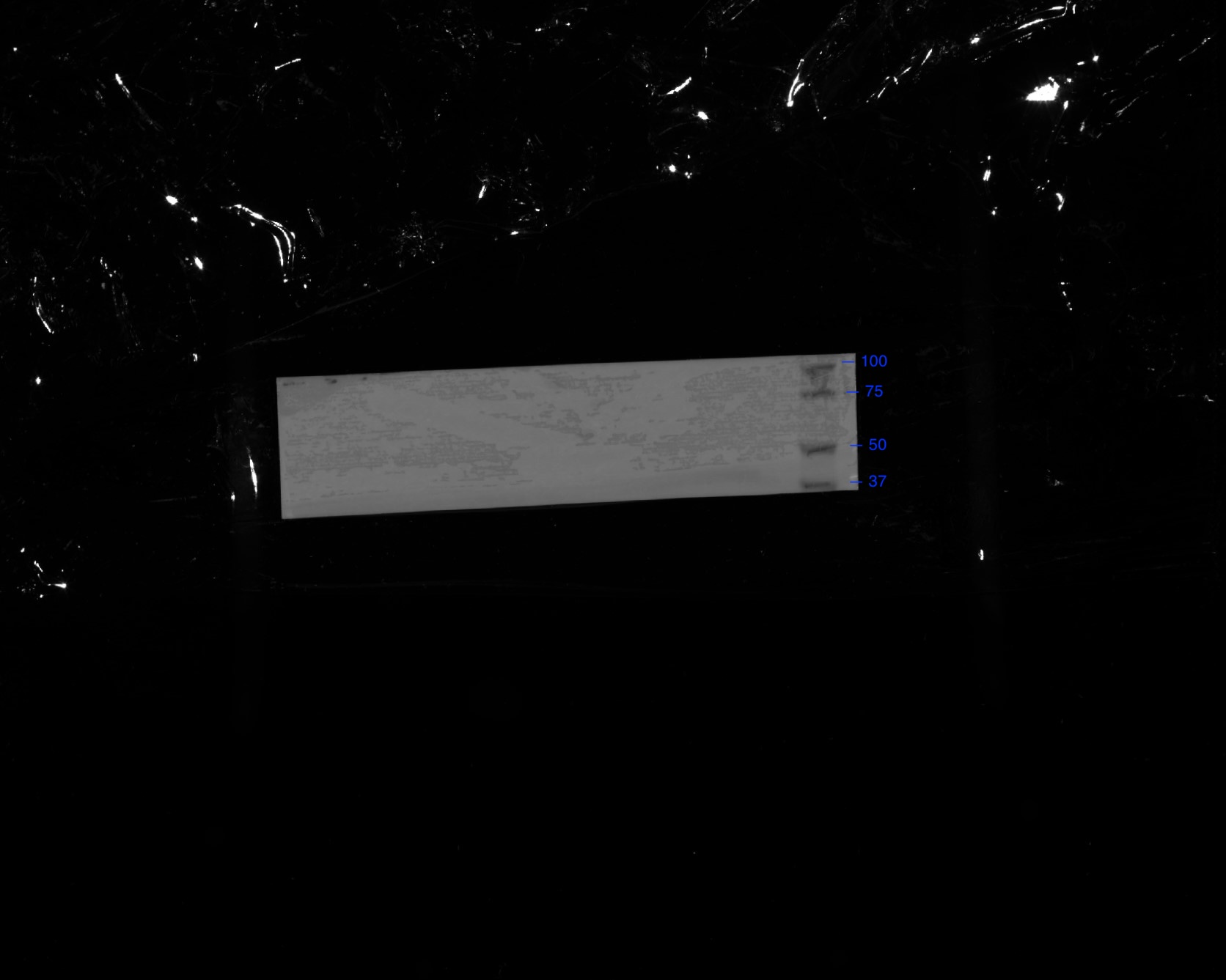

Supplement: Supplementary file 4 — Source data Fig. 1 [file 44319_2026_700_MOESM4_ESM.zip › Figure 1 -revised/1E - Western blot/NELFA(Ponceau S).jpg]

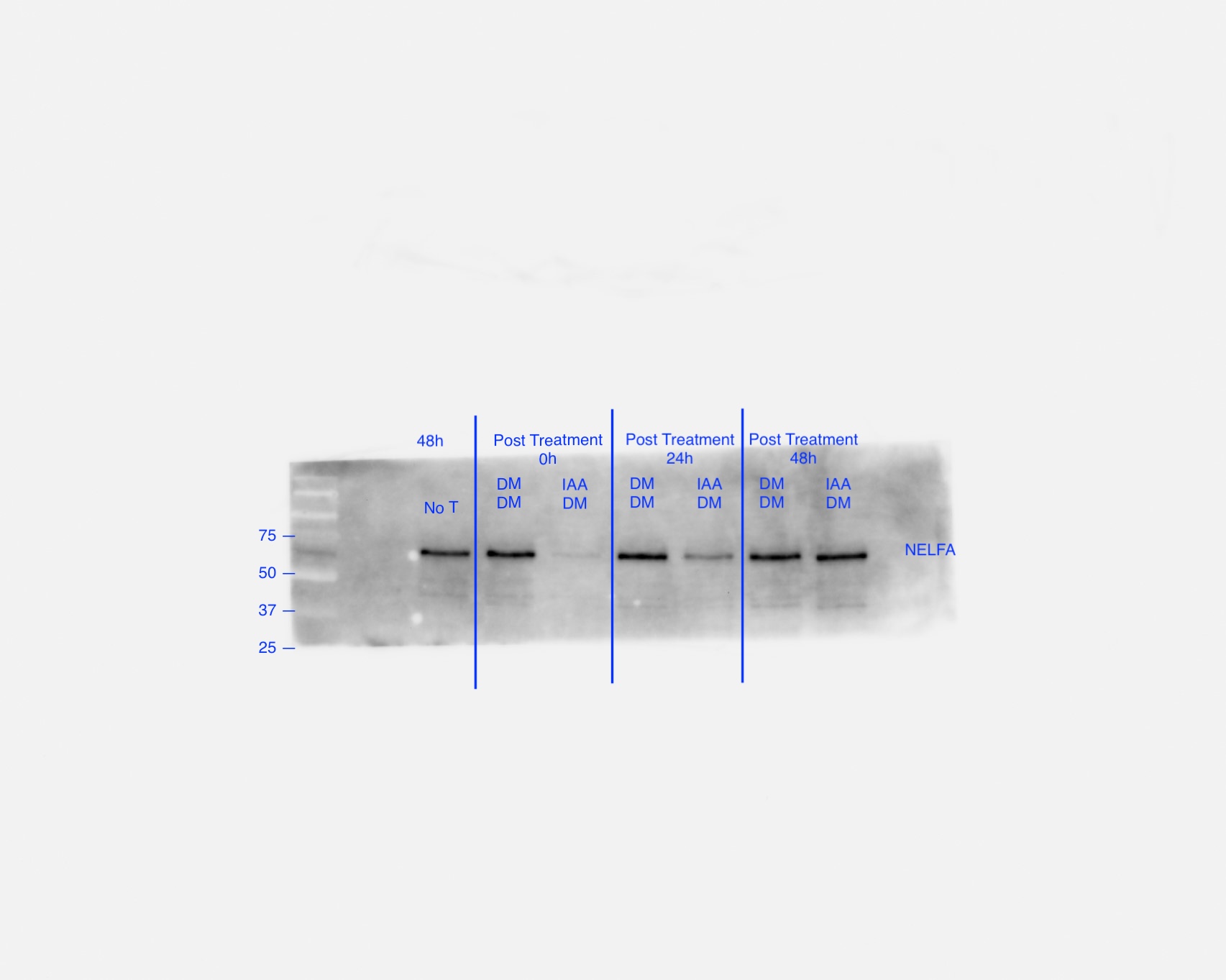

Supplement: Supplementary file 5 — Source data Fig. 2 [file 44319_2026_700_MOESM5_ESM.zip › Figure 2/2B - Western blot/NELFA(Chemiluminescence).jpg]

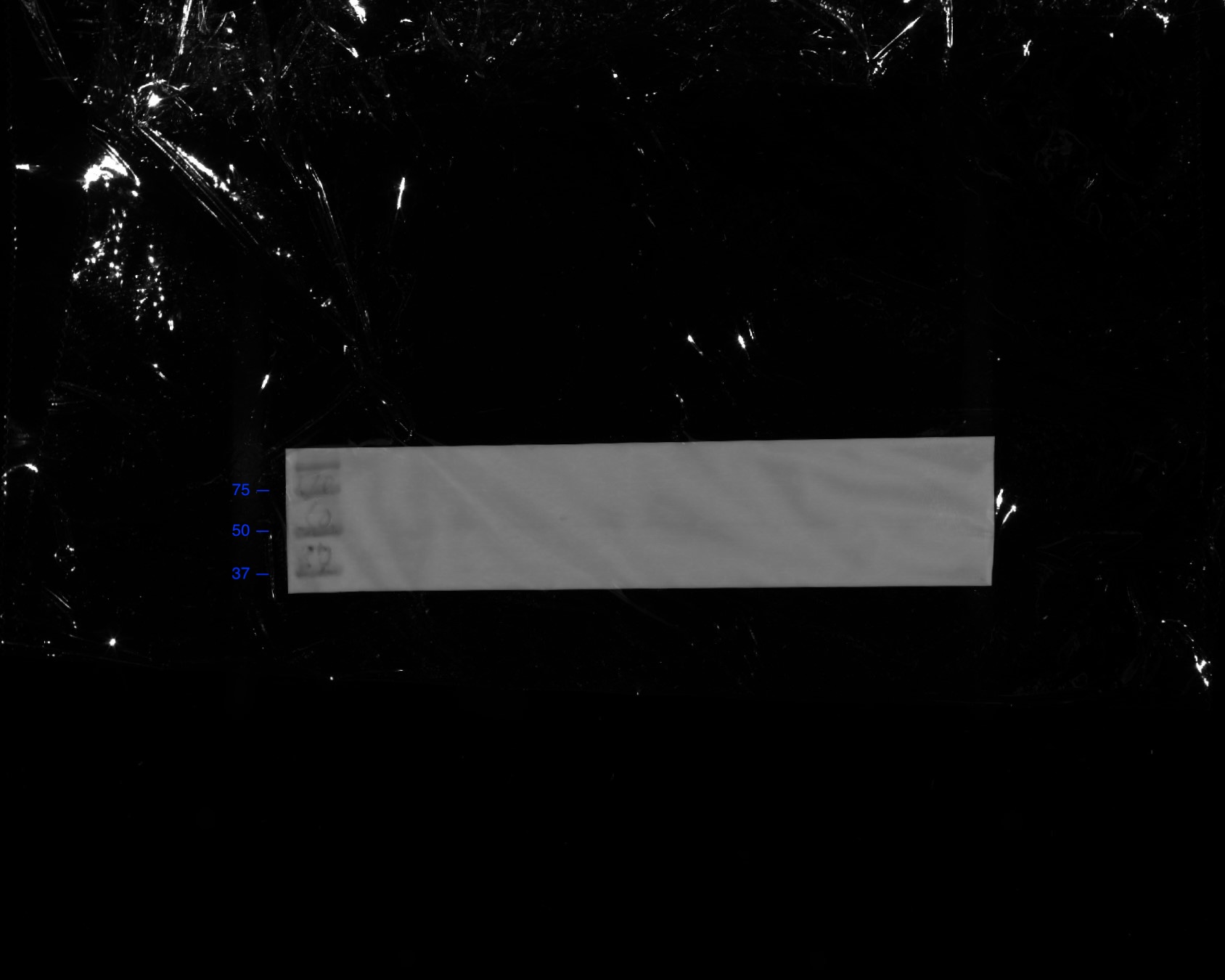

Supplement: Supplementary file 5 — Source data Fig. 2 [file 44319_2026_700_MOESM5_ESM.zip › Figure 2/2B - Western blot/NELFC TUB(Ponceau S).jpg]

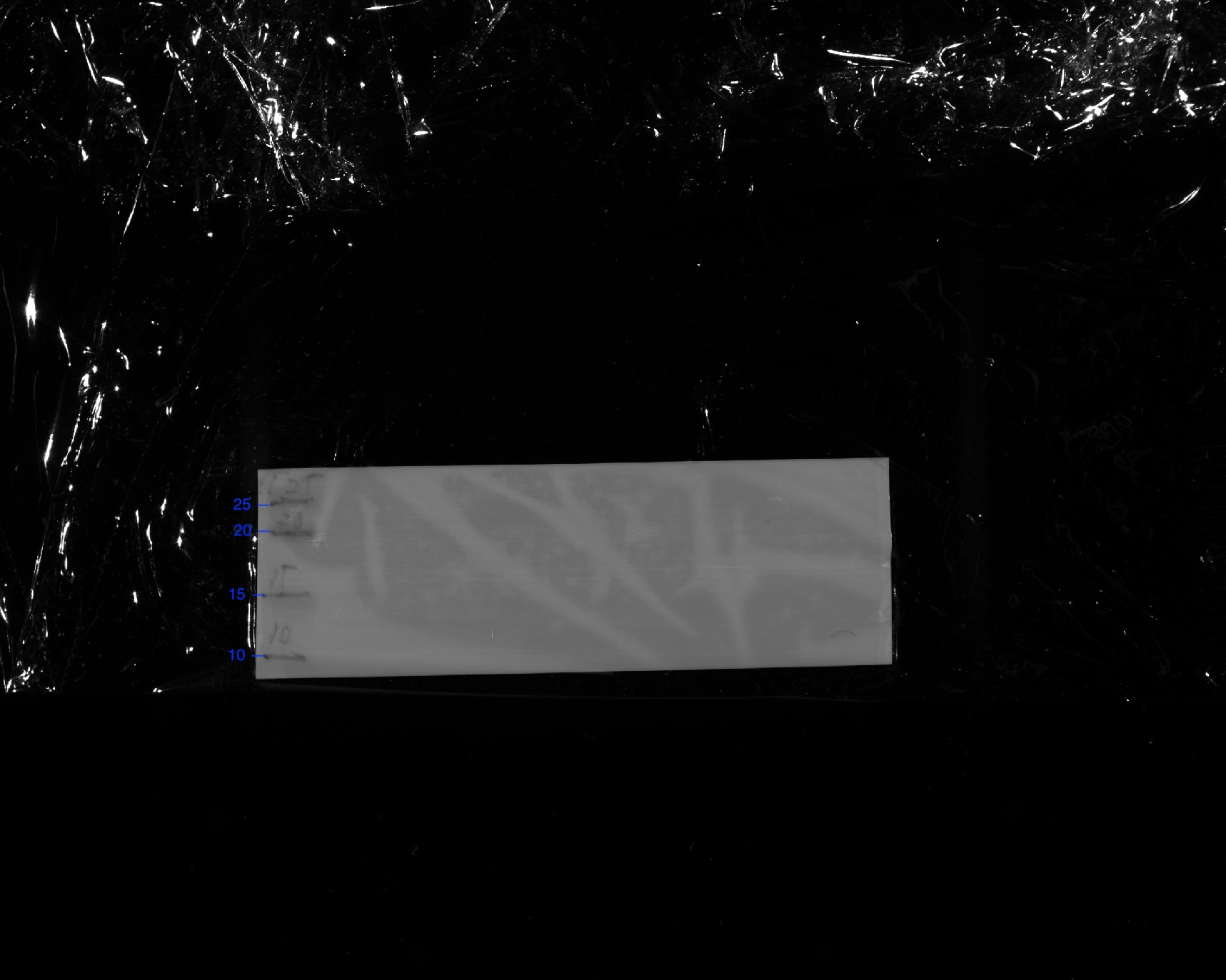

Supplement: Supplementary file 5 — Source data Fig. 2 [file 44319_2026_700_MOESM5_ESM.zip › Figure 2/2B - Western blot/P21(Ponceau S).jpg]

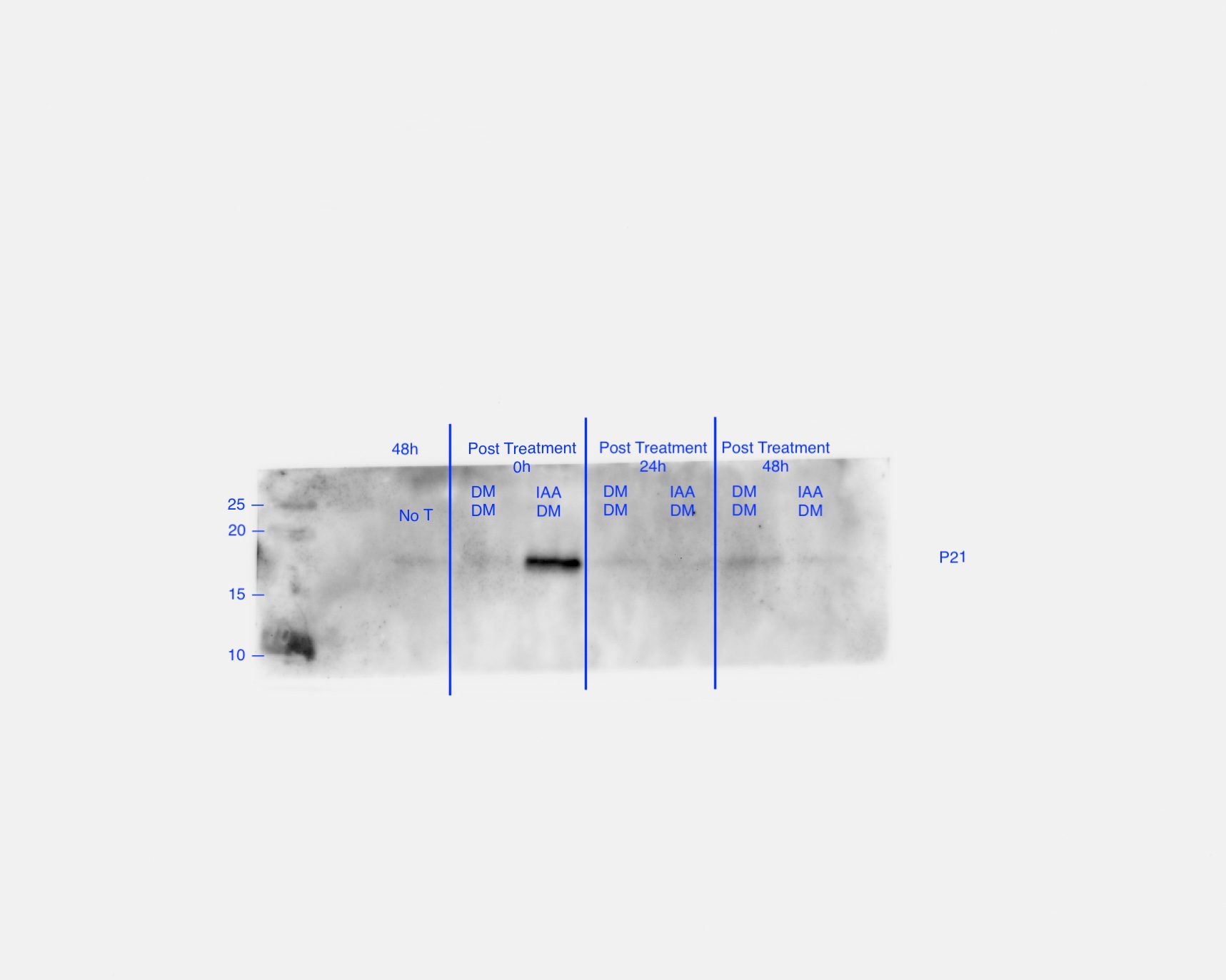

Supplement: Supplementary file 5 — Source data Fig. 2 [file 44319_2026_700_MOESM5_ESM.zip › Figure 2/2B - Western blot/P21(Chemiluminescence).jpg]

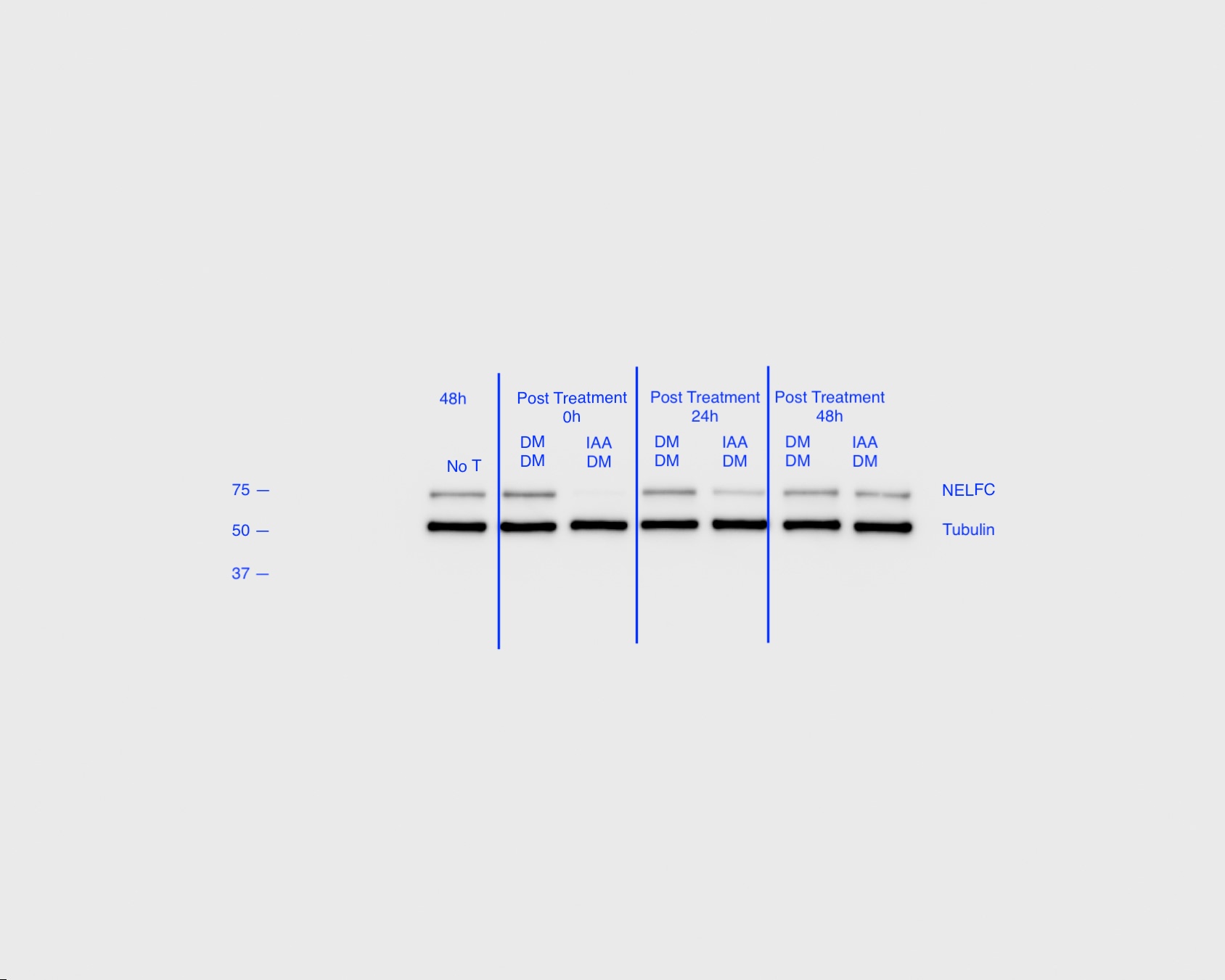

Supplement: Supplementary file 5 — Source data Fig. 2 [file 44319_2026_700_MOESM5_ESM.zip › Figure 2/2B - Western blot/NELFC TUB(Chemiluminescence).jpg]

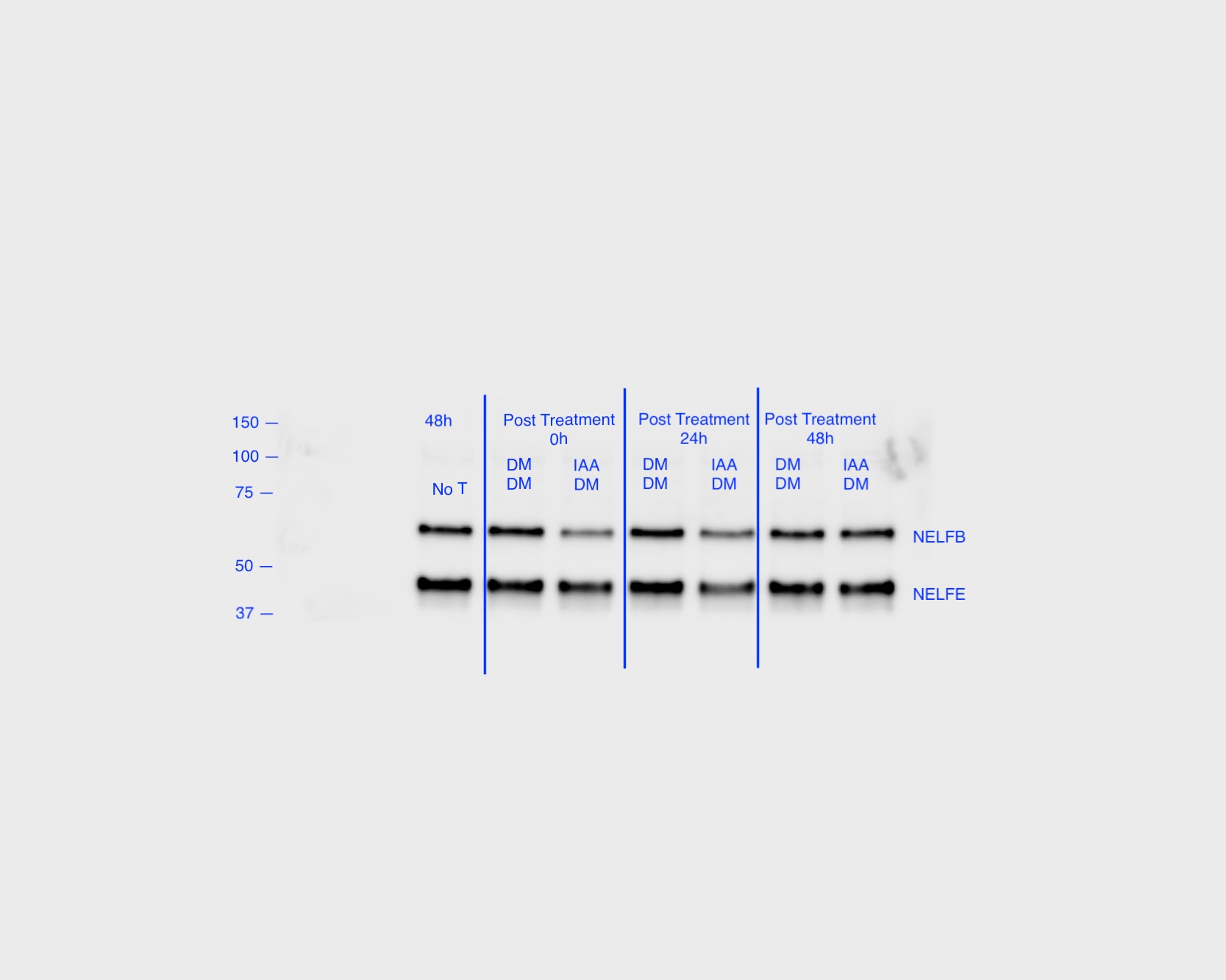

Supplement: Supplementary file 5 — Source data Fig. 2 [file 44319_2026_700_MOESM5_ESM.zip › Figure 2/2B - Western blot/NELFB NELFE(Chemiluminescence).jpg]

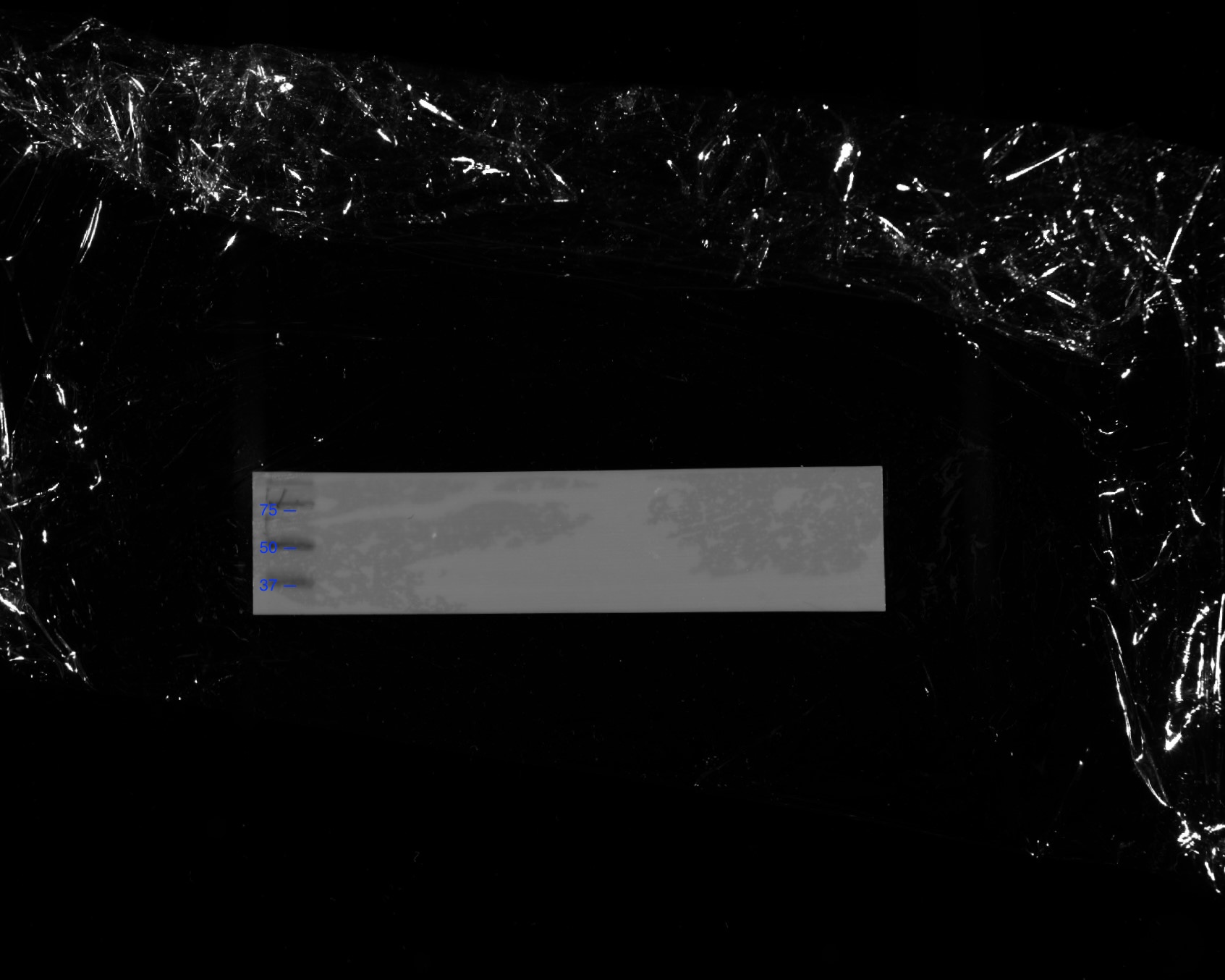

Supplement: Supplementary file 5 — Source data Fig. 2 [file 44319_2026_700_MOESM5_ESM.zip › Figure 2/2B - Western blot/P57(Ponceau S).jpg]

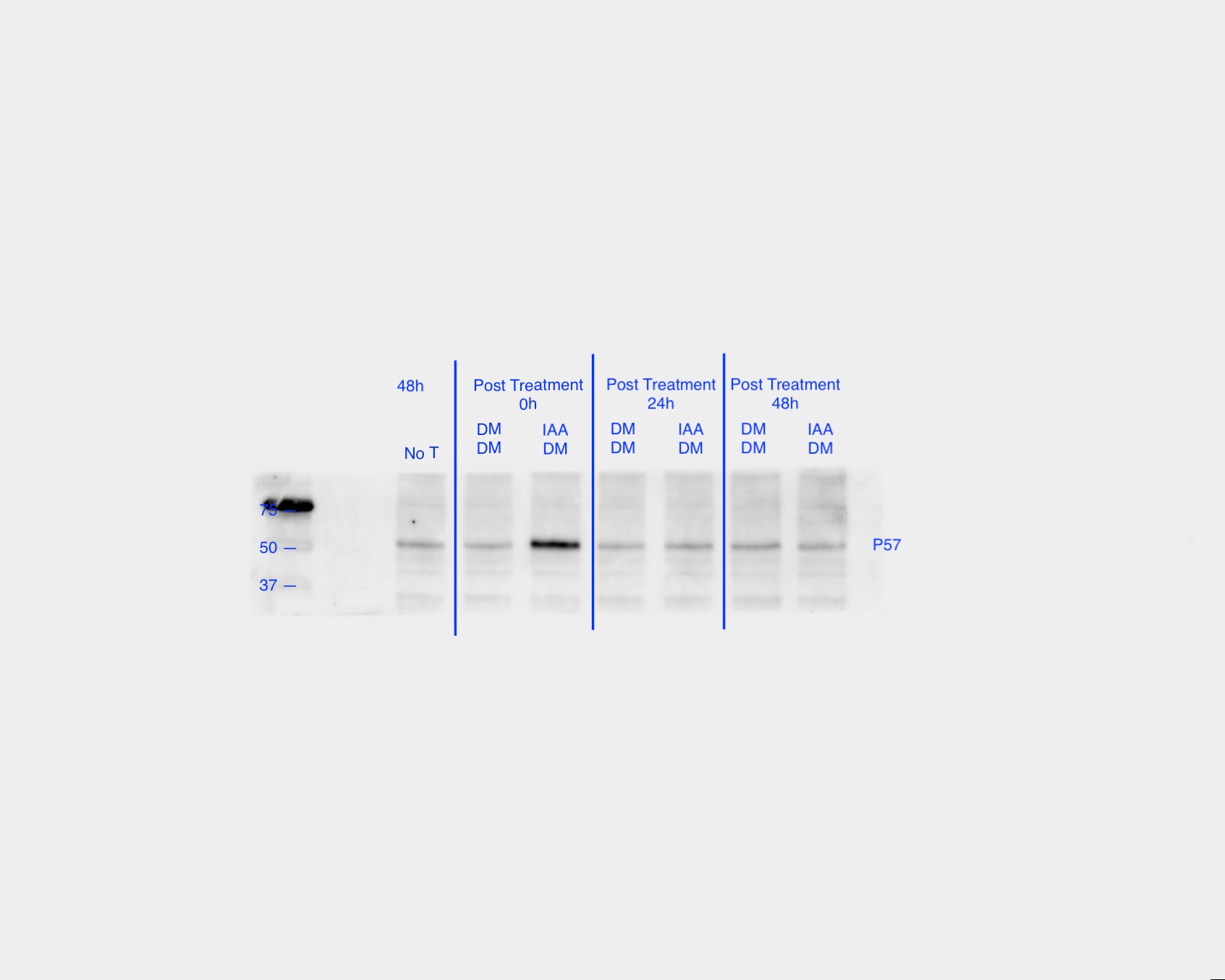

Supplement: Supplementary file 5 — Source data Fig. 2 [file 44319_2026_700_MOESM5_ESM.zip › Figure 2/2B - Western blot/P57(Chemiluminescence).jpg]

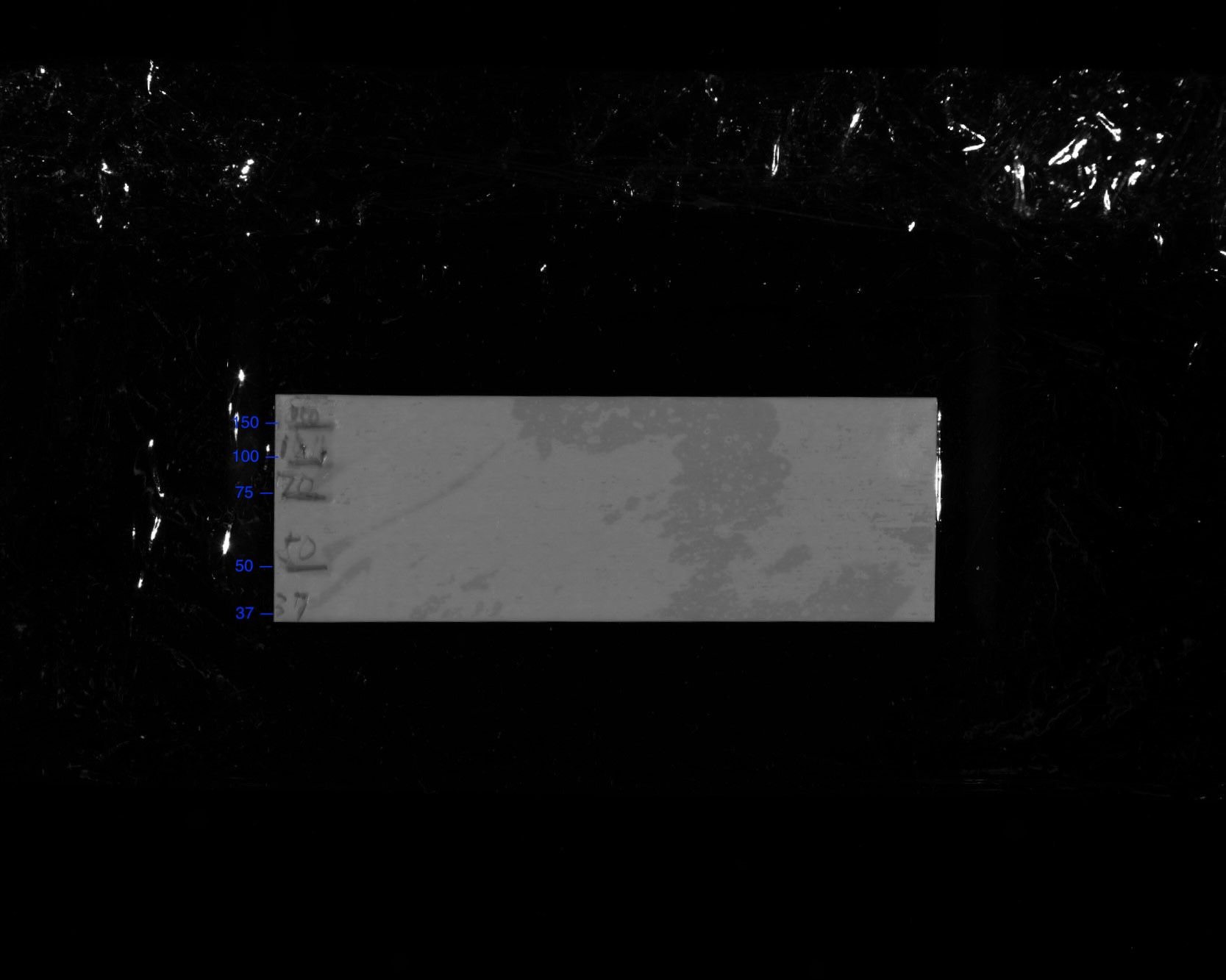

Supplement: Supplementary file 5 — Source data Fig. 2 [file 44319_2026_700_MOESM5_ESM.zip › Figure 2/2B - Western blot/NELFB NELFE(Ponceau S).jpg]

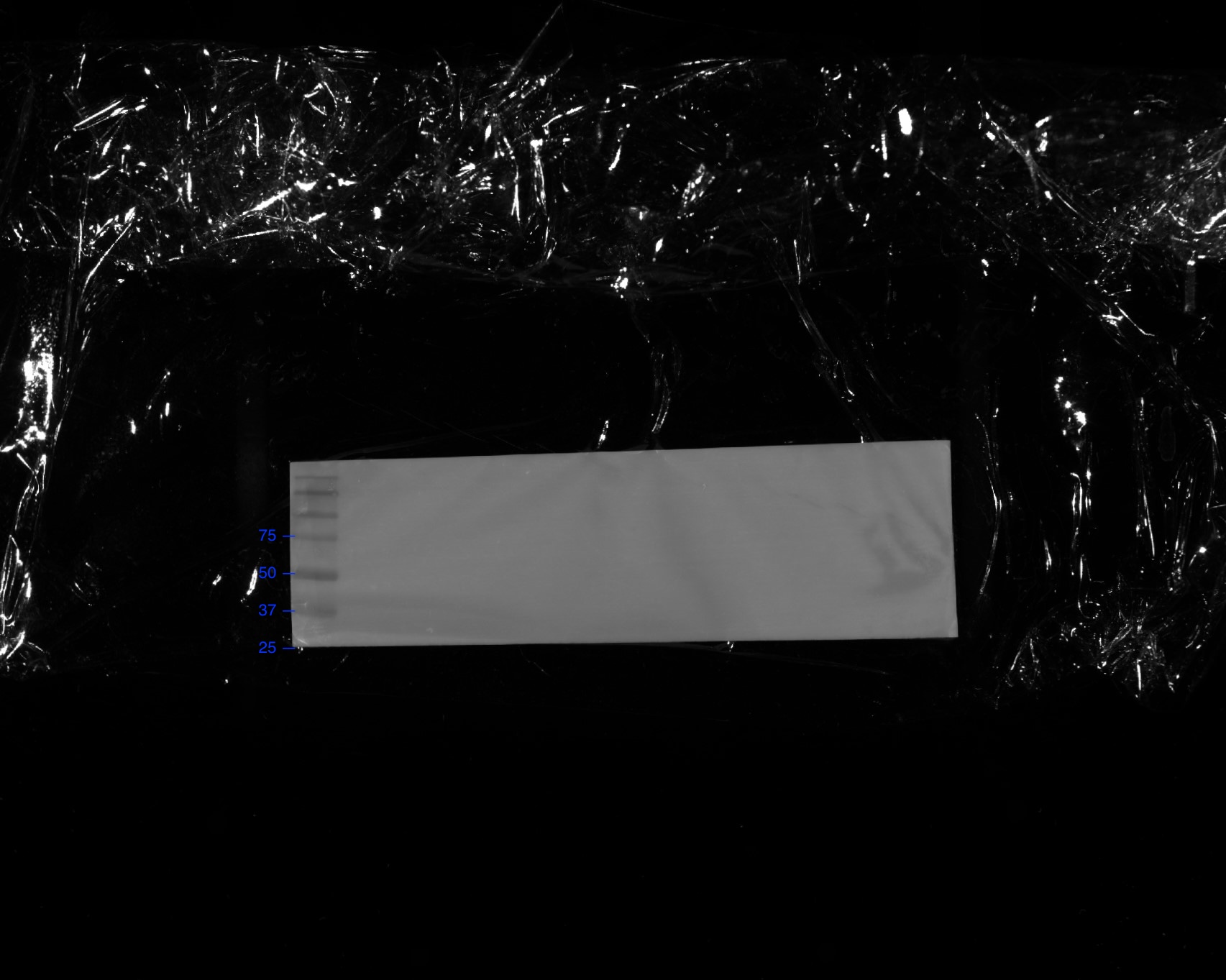

Supplement: Supplementary file 5 — Source data Fig. 2 [file 44319_2026_700_MOESM5_ESM.zip › Figure 2/2B - Western blot/NELFA(Ponceau S).jpg]
