# Supplementary material for: NELF prevents transcriptional readthrough into DNA replication zones in cancer cells
Source: EMBO Rep. 2026 Feb 20;27(7):1720–58. doi: 10.1038/s44319-026-00700-z (PMC13076867; doi:10.1038/s44319-026-00700-z)
Supplement: Supplementary file 6 — Expanded View Figures [file 44319_2026_700_MOESM6_ESM.pdf]

Expanded View Figures

Figure EV1. Expression of *NELFCD* transcripts is highly upregulated in colorectal cancer cells.

(**A**) Log_2 fold change of T vs N of the Pol II transcription-associated genes. Upregulated (>1) and down-regulated (<-1) genes are highlighted in red and blue, respectively. *NELFCD* gene is indicated with an arrow. (**B**) Volcano plot of the log2 fold change of Tumors (T) vs Normal tissues (N) on 835 cell cycle-related genes in COAD. *CDKN1A*, *CDKN1B*, and *CDKN1C* genes are indicated in red. The numbers of tumor (T) and normal (N) samples for COAD are provided in Table EV1. Box plots show the median (center line) and interquartile range (box, 25th–75th percentiles); whiskers indicate 1.5×IQR. (**C**) Log2 of the RNA expression levels of *NELFCD*, *SUPT4H1*, *CDKN1A*, and *CDKN1C* genes in N and T of the indicated tissues and tumors are compared. Box plots show the median (center line) and interquartile range (box, 25th–75th percentiles); whiskers indicate 1.5×IQR. Statistical test: Wilcoxon signed-rank test. *P* values are shown. The numbers of tumor (T) and normal (N) samples for each tissue types are provided in Table EV1. (**D**) Log_2 fold change of T vs N of *NELFCD* across indicated tissue types. (**E**) Comparative proteomic analysis of NELF subunits and SPT4 in human primary colon cancers and its adjacent tissues. Statistical test: negative binomial distribution-based Wald test. *P* values are shown. Box plot: minimal-to-maximal value, box center line: median, bounds of box: interquartile (25 and 75%). (**F**) Schematic model of transcription addicted by NELF in tumor.

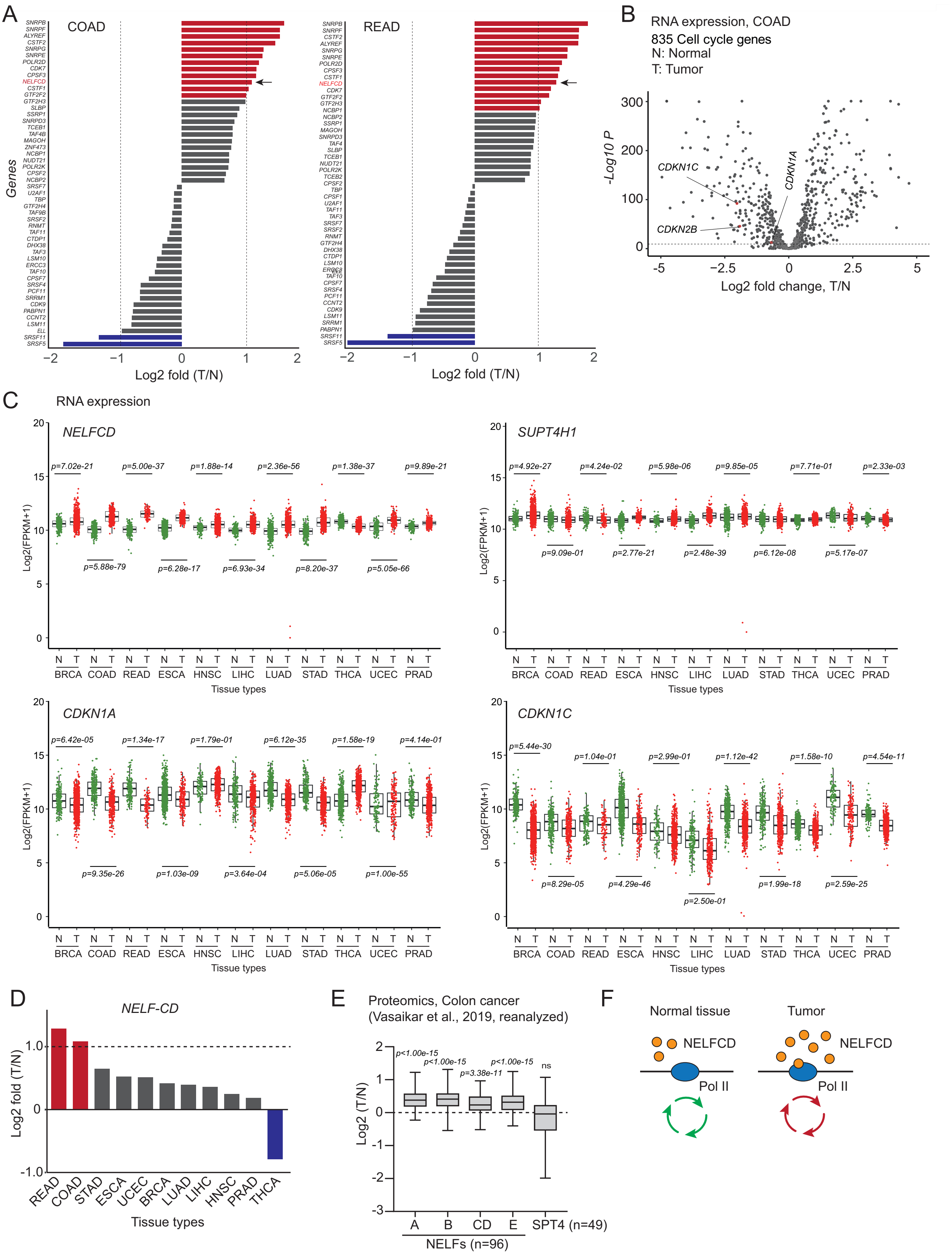
A
COAD
READ
Genes
NELFCD
Log2 fold (T/N)
B
RNA expression, COAD
835 Cell cycle genes
N: Normal
T: Tumor
-Log10 P
CDKN1C
CDKN2B
CDKN1A
Log2 fold change, T/N
C
RNA expression
NELFCD
SUPT4H1
CDKN1A
CDKN1C
Log2(FPKM+1)
N T
BRCA COAD READ ESCA HNSC LIHC LUAD STAD THCA UCEC PRAD
Tissue types
p=7.02e-21 p=5.00e-37 p=1.88e-14 p=2.36e-56 p=1.38e-37 p=9.89e-21
p=5.88e-79 p=6.28e-17 p=6.93e-34 p=8.20e-37 p=5.05e-66
p=4.92e-27 p=4.24e-02 p=5.98e-06 p=9.85e-05 p=7.71e-01 p=2.33e-03
p=9.09e-01 p=2.77e-21 p=2.48e-39 p=6.12e-08 p=5.17e-07
p=6.42e-05 p=1.34e-17 p=1.79e-01 p=6.12e-35 p=1.58e-19 p=4.14e-01
p=9.35e-26 p=1.03e-09 p=3.64e-04 p=5.06e-05 p=1.00e-55
p=5.44e-30 p=1.04e-01 p=2.99e-01 p=1.12e-42 p=1.58e-10 p=4.54e-11
p=8.29e-05 p=4.29e-46 p=2.50e-01 p=1.99e-18 p=2.59e-25
D
NELF-CD
Log2 fold (T/N)
READ COAD STAD ESCA UCEC BRCA LUAD LIHC HNSC PRAD THCA
Tissue types
E
Proteomics, Colon cancer
(Vasaikar et al., 2019, reanalyzed)
Log2 (T/N)
p<1.00e-15 p<1.00e-15 p=3.38e-11 p<1.00e-15 ns
A B CD E SPT4 (n=49)
NELFs (n=96)
F
Normal tissue
Tumor
NELFCD
Pol II

 © The Author(s)

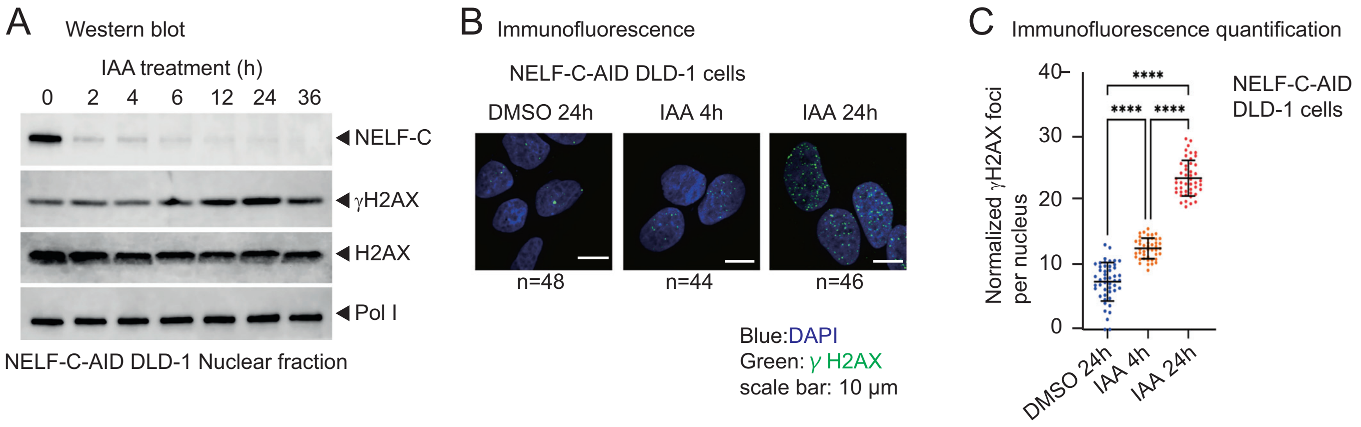

Figure EV2. Loss of NELF induces DNA damage.

(**A**) Western blot of NELF-C-AID DLD-1 cell nuclear fraction using the indicated antibodies. Treatment time (h) of IAA is also indicated. Western blot images of NELF-C and Pol I are reused in Fig. 1E. (**B**) Immunofluorescent image of parental and NELF-C-AID DLD-1 cells in 24 h DMSO and 4 h or 24 h IAA. Merged images with DAPI (blue) and γH2AX antibody (green). Scale bar size is 10 µm. Number of cells for quantification (for Fig. EV2C) is indicated. (**C**) Quantification of normalized γH2AX foci per nucleus in NELF-C-AID DLD-1 cells in 24 h DMSO and 4 h or 24 h IAA. Statistical test: Kruskal–Wallis test for NELC-AID cells. ns: not significant, ****$P < 0.0001$. Error bars represented the mean ± SD (biological replicates, $n = 2$). NELF-C-AID DLD-1 cells in 24 h DMSO ($n = 48$) and 4 h ($n = 44$) or 24 h IAA ($n = 46$).

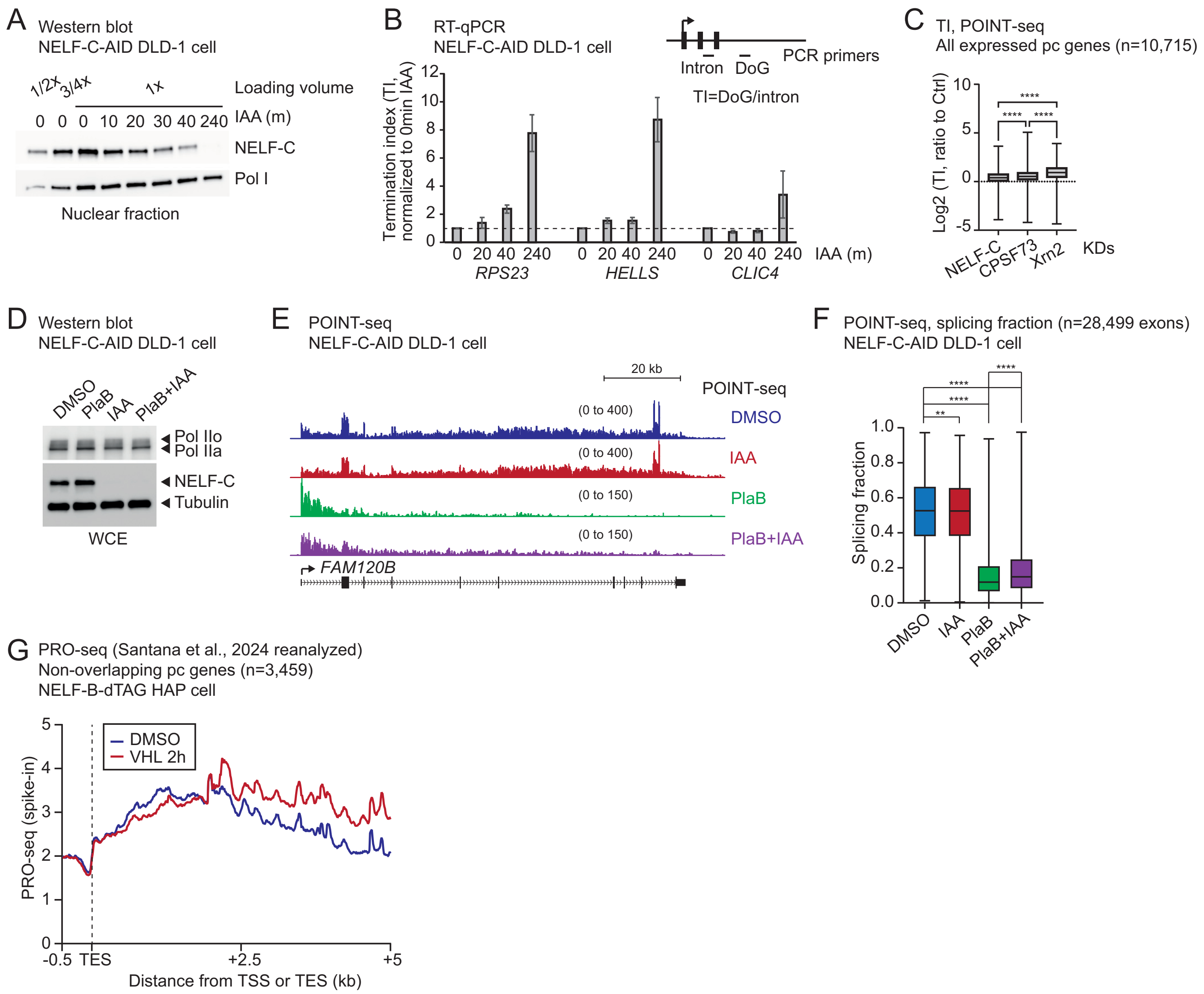

Figure EV3. Acute NELF depletion induces a Pol II transcription termination failure.

(**A**) Western blot of NELF-C-AID DLD-1 cell nuclear fraction using the indicated antibodies. Loading volume (x1/2, x3/4 or x1) and treatment time (min) of IAA are indicated. (**B**) RT-qPCR analysis for TI (DoG/intron) of *RPS23*, *HELLS*, and *CLIC4* genes in NELF-C-AID DLD-1 cell. Treatment time of IAA is indicated. Primer information is available in Table EV2. Error bars represented the mean ± SEM (biological replicates, $n = 3$). (**C**) Box plots of TI of all expressed genes in indicated AID-tagged cells. Wilcoxon rank-sum test. ***$P < 0.001$. Box plot: minimal-to-maximal value, box center line: median, bounds of box: interquartile (25 and 75%). (**D**) Western blot of NELF-C-AID DLD-1 WCE (4 h DMSO, PlaB, IAA, PlaB+IAA) using the indicated antibodies. (**E**) Example view of POINT-seq on *FAM120B* genes in NELF-C-AID DLD-1 cells (4 h DMSO, PlaB, IAA, PlaB+IAA). (**F**) Quantification of splicing fraction of POINT-seq in NELF-C-AID DLD-1 cells (4 h DMSO, IAA, PlaB, PlaB+IAA). Statistical test: Friedman test. **$P = 0.001$, ****$P < 0.0001$. Box plot: minimal-to-maximal value, box center line: median, bounds of box: interquartile (25 and 75%). (**G**) Metagene analysis of PRO-seq around PAS of non-overlapping pc genes in NELF-B-dTAG HAP cells (DMSO and a protein degradation inducer VHL for 2 h).

 © The Author(s)

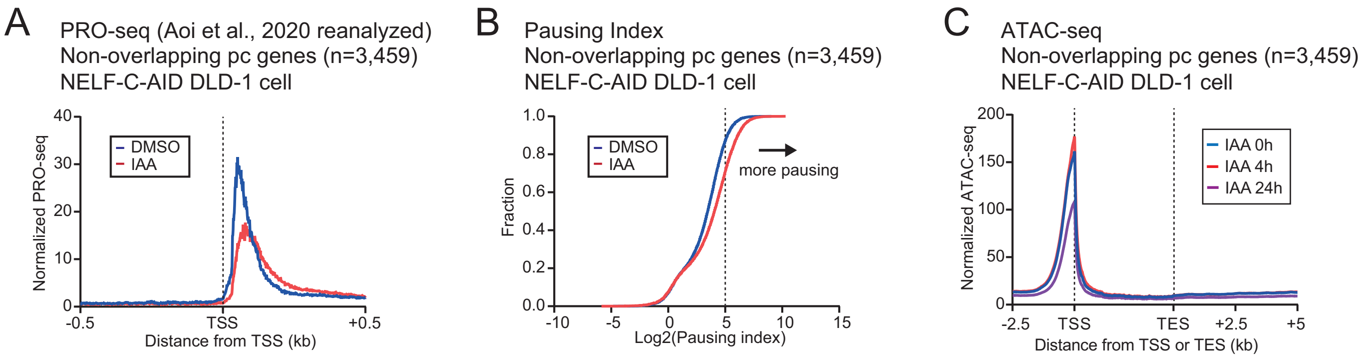

Figure EV4. Acute loss of NELF increases Pol II pausing index and transcriptional initiation.

(**A**) Metagene analysis of published PRO-seq on TSS −/+0.5 kb of non-overlapping pc genes. (**B**) Density plot of the pausing index of non-overlapping pc genes calculated from DLD-1 NELF-C AID PRO-seq cells treated with DMSO (blue) or IAA for 4 h (red). A shift of the density plot to the right indicates a higher pausing. (**C**) Metagene analysis of ATAC-seq for non-overlapping pc genes in NELF-C-AID DLD-1 cells (0, 4, and 24 h IAA).

© The Author(s)

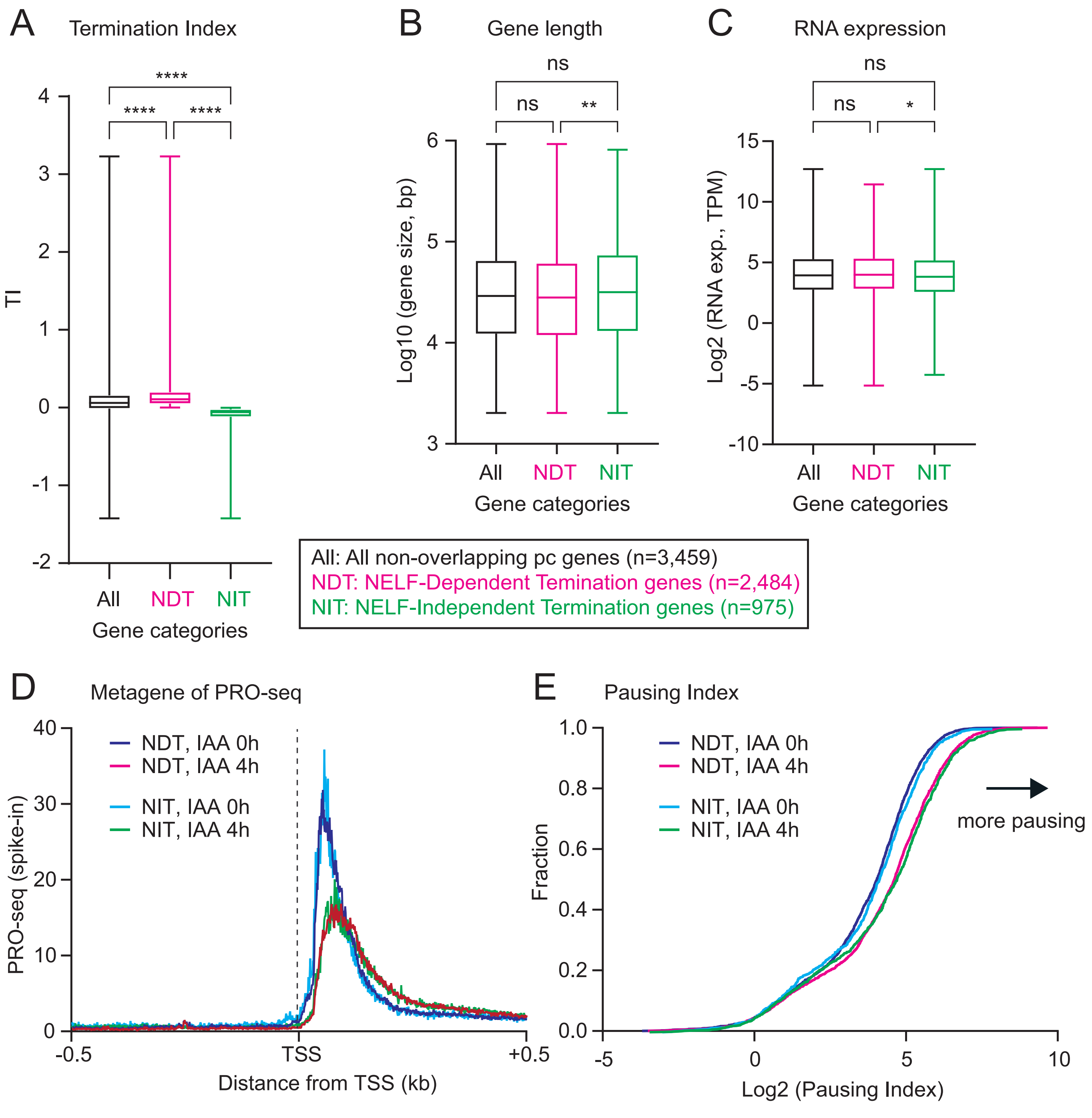

Figure EV5. Characters of highly and lowly NELF-dependent termination genes.

(**A**) Box plots of $\log_2$(TI) in POINT-seq in NELF-C-AID DLD-1 cells (4 h vs 0 h IAA). Genes were classified to NELF-dependent termination (NDT, $n = 2484$) and NELF-independent termination (NIT, $n = 975$) gene categories by TI. N values are indicated on the figure panel. Statistical test: Wilcoxon signed-rank test. ****$P < 0.0001$. Box plot: minimal-to-maximal value, box center line: median, bounds of box: interquartile (25 and 75%). (**B**) Box plots of $\log_{10}$ (gene size, bp) of the indicated categories. Statistical test: Kruskal–Wallis test. **$P = 0.0097$, ns: not significant. Box plot: minimal-to-maximal value, box center line: median, bounds of box: interquartile (25 and 75%). (**C**) Box plots of $\log_2$ (RNA expression level, TPM) of the indicated categories. Statistical test: Kruskal–Wallis test. *$P = 0.0176$, ns: not significant. Box plot: minimal-to-maximal value, box center line: median, bounds of box: interquartile (25 and 75%). (**D**) Metagene analysis of published PRO-seq on TSS $-/+0.5$ kb of the indicated categories. (**E**) Density plot of the pausing index of the indicated categories calculated from NELF-C AID DLD-1 cells.

 © The Author(s)

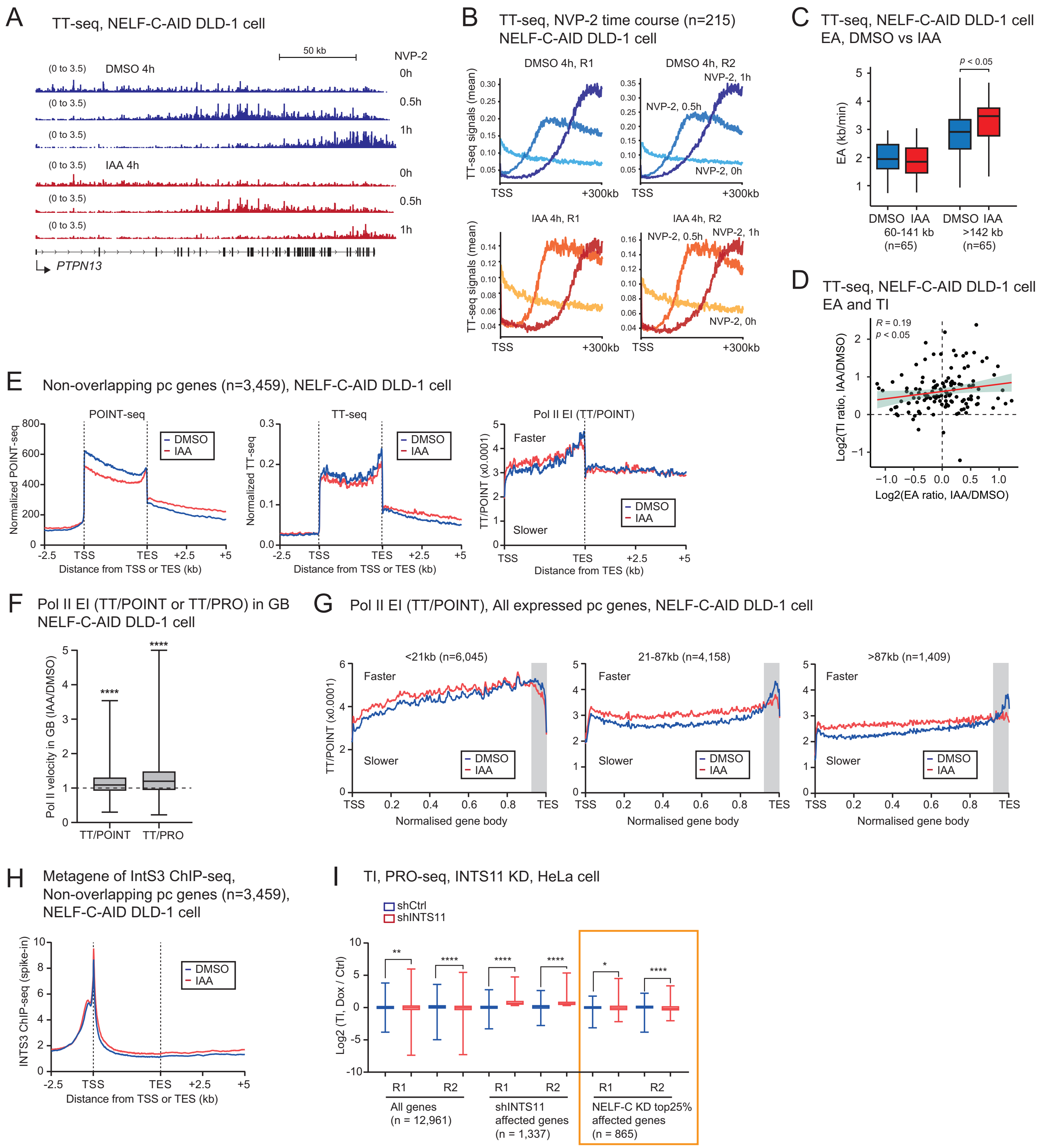
A
TT-seq, NELF-C-AID DLD-1 cell
50 kb
NVP-2
(0 to 3.5) DMSO 4h
0h
0.5h
1h
(0 to 3.5) IAA 4h
PTPN13
B
TT-seq, NVP-2 time course (n=215)
NELF-C-AID DLD-1 cell
DMSO 4h, R1
DMSO 4h, R2
IAA 4h, R1
IAA 4h, R2
NVP-2, 1h
NVP-2, 0.5h
NVP-2, 0h
TT-seq signals (mean)
TSS
+300kb
C
TT-seq, NELF-C-AID DLD-1 cell
EA, DMSO vs IAA
P < 0.05
EA (kb/min)
DMSO IAA
60-141 kb
(n=65)
>142 kb
(n=65)
D
TT-seq, NELF-C-AID DLD-1 cell
EA and TI
R = 0.19
p < 0.05
Log2(TI ratio, IAA/DMSO)
Log2(EA ratio, IAA/DMSO)
E
Non-overlapping pc genes (n=3,459), NELF-C-AID DLD-1 cell
POINT-seq
Normalized POINT-seq
TT-seq
Normalized TT-seq
Pol II EI (TT/POINT)
TT/POINT (x0.0001)
Faster
Slower
DMSO
IAA
Distance from TSS or TES (kb)
Distance from TES (kb)
F
Pol II EI (TT/POINT or TT/PRO) in GB
NELF-C-AID DLD-1 cell
Pol II velocity in GB (IAA/DMSO)
TT/POINT
TT/PRO
G
Pol II EI (TT/POINT), All expressed pc genes, NELF-C-AID DLD-1 cell
<21kb (n=6,045)
21-87kb (n=4,158)
>87kb (n=1,409)
Normalised gene body
H
Metagene of IntS3 ChIP-seq,
Non-overlapping pc genes (n=3,459),
NELF-C-AID DLD-1 cell
INTS3 ChIP-seq (spike-in)
Distance from TSS or TES (kb)
I
TI, PRO-seq, INTS11 KD, HeLa cell
shCtrl
shINTS11
Log2 (TI, Dox / Ctrl)
R1
R2
All genes
(n = 12,961)
shINTS11
affected genes
(n = 1,337)
NELF-C KD top25%
affected genes
(n = 865)

Figure EV6. Characters of highly and lowly NELF-dependent termination genes.

(**A**) Example view of TT-seq on *PTPN13* gene in the indicated cell line treated for 4 h with DMSO or IAA, followed by NVP-2 treatment for 0, 0.5, or 1 h. (**B**) Profiles of TT-seq mean signal in NELF-C-AID DLD-1 cells treated for 4 h with DMSO or IAA, followed by NVP-2 treatment for 0, 0.5, or 1 h. Data for 2 biological replicates with ≥300 kb genes are shown. (**C**) Box plots of elongation activity (EA) derived from TT-seq. Data for short and long genes are shown. $n = 65$. Statistical test: Wilcoxon rank-sum test. Box plot: minimal-to-maximal value, box center line: median, bounds of box: interquartile (25 and 75%). P value is 0.029. (**D**) Scatter plot showing the correlation between EA and TI. The regression line (red) and the 95% confidence interval (green) are also shown. $n = 129$. Statistical method: Spearman correlation. P value is 0.036. (**E**) Metagene of POINT-seq, TT-seq, Pol II EI (TT-seq/POINT-seq) for non-overlapping pc genes in NELF-C-AID DLD-1 cells treated for 4 h with DMSO or IAA. (**F**) Box plots of Pol II EI (TT-seq/POINT-seq and TT-seq/PRO-seq) for non-overlapping pc genes ($n = 3459$) in NELF-C-AID DLD-1 cells treated for 4 h with DMSO or IAA. Statistical test: Kruskal–Wallis test. ****$P < 0.0001$. Box plot: minimal-to-maximal value, box center line: median, bounds of box: interquartile (25 and 75%). (**G**) Pol II EI for indicated three gene-length classes of normalized non-overlapping pc genes in NELF-C-AID DLD-1 cells treated for 4 h with DMSO or IAA. (**H**) INTS3 ChIP-seq profile across normalized transcription units of non-overlapping pc genes in NELF-C-AID DLD-1 cells treated for 4 h with DMSO or IAA. (**I**) Box plots of the log2 of the Dox/Ctrl termination index, two biological replicates in all genes, genes with transcription readthrough which is based on previous study (Dasilva et al, 2021), and NELF-C KD affected genes (top 25%, highlighted in orange box). 0: no change in TI index upon shCtrl or shINTS11 induction by Dox. The number of genes in each category is indicated on the figure. Statistical test: Kruskal–Wallis test. *$P = 0.039$, **$P = 0.0019$, ****$P < 0.0001$. Box plot: minimal-to-maximal value, box center line: median, bounds of box: interquartile (25 and 75%).

© The Author(s)

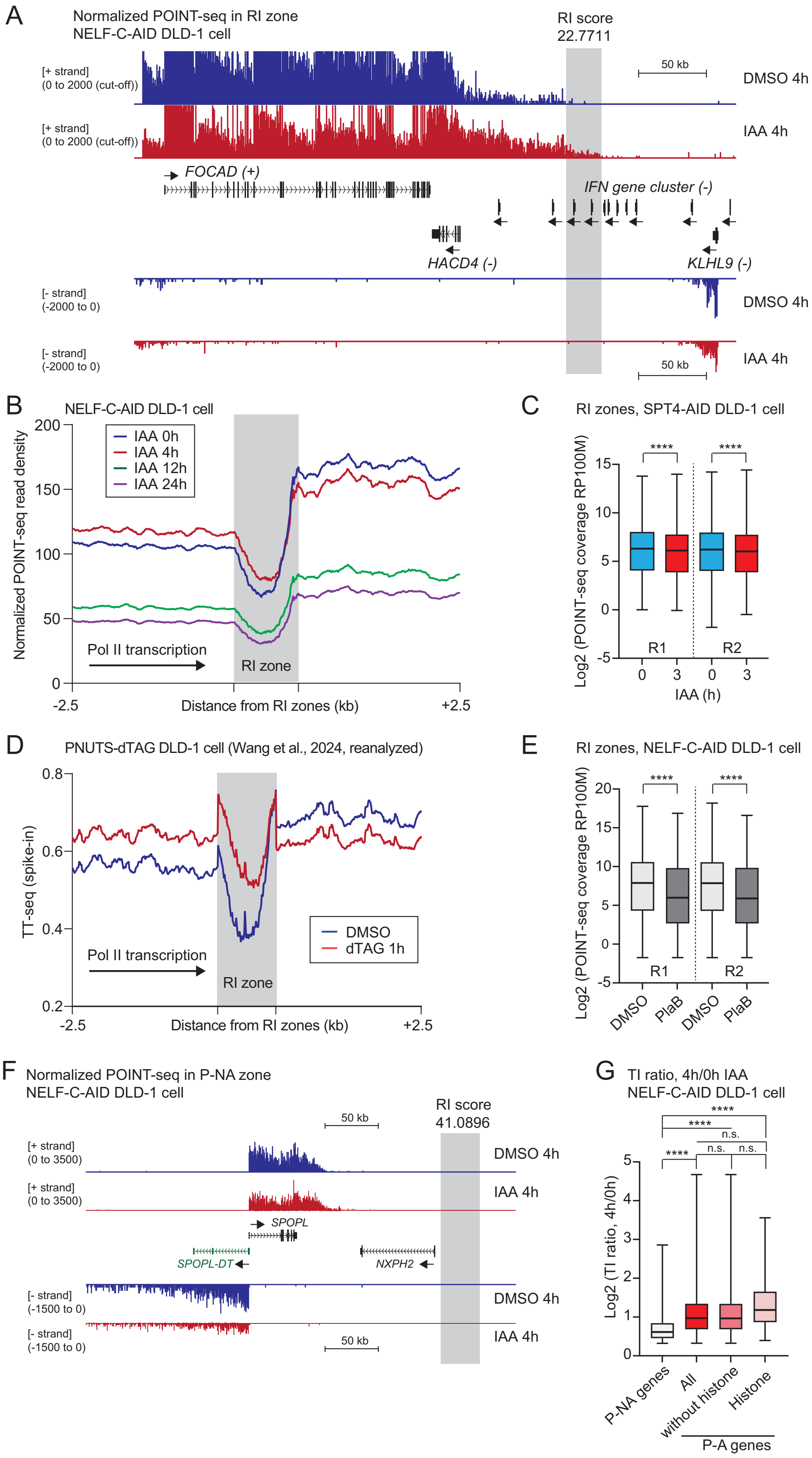
A
Normalized POINT-seq in RI zone
NELF-C-AID DLD-1 cell
RI score
22.7711
50 kb
[+ strand]
(0 to 2000 (cut-off))
DMSO 4h
IAA 4h
FOCAD (+)
IFN gene cluster (-)
HACD4 (-)
KLHL9 (-)
[- strand]
(-2000 to 0)
B
NELF-C-AID DLD-1 cell
IAA 0h
IAA 4h
IAA 12h
IAA 24h
Normalized POINT-seq read density
Pol II transcription
RI zone
Distance from RI zones (kb)
C
RI zones, SPT4-AID DLD-1 cell
Log2 (POINT-seq coverage RP100M)
R1
R2
IAA (h)
D
PNUTS-dTAG DLD-1 cell (Wang et al., 2024, reanalyzed)
TT-seq (spike-in)
DMSO
dTAG 1h
E
RI zones, NELF-C-AID DLD-1 cell
PlaB
F
Normalized POINT-seq in P-NA zone
RI score
41.0896
(0 to 3500)
(-1500 to 0)
SPOPL
SPOPL-DT
NXPH2
G
TI ratio, 4h/0h IAA
Log2 (TI ratio, 4h/0h)
P-NA genes
All
without histone
Histone
P-A genes
n.s.

Figure EV7. Acute depletion of NELF-C causes Pol II transcription invasion into DNA replication initiation zone.

(**A**) Example view of SIRV-normalized POINT-seq on *FOCAD* gene adjacent to RI zone in NELF-C-AID DLD-1 cells (4 h DMSO and IAA). Normalized POINT-seq signals are cut-off at 2000. RI zone is highlighted in gray. RI score: 22.7711. Both (+) and (−) strands are shown. (**B**) Metagene analysis of POINT-seq on RI zones -/+2.5 kb in NELF-C-AID DLD-1 cells (0, 4, 12, 24 h IAA). (**C**) Box plots of normalized POINT-seq signals in RI zones in SPT4-AID DLD-1 cells (0 h and 3 h IAA). Two replicates are shown. (R1: $n = 9997$ RI zones, R2: $n = 9597$ RI zones). Statistical test: Wilcoxon test. ****$P < 0.0001$. Box plot: minimal-to-maximal value, box center line: median, bounds of box: interquartile (25 and 75%). (**D**) Metagene analysis of TT-seq on RI zones −/+2.5 kb in PNUTS-dTAG DLD-1 cells (0 and 1 h dTAG). (**E**) Box plots of normalized POINT-seq signals in RI zones in NELF-C-AID DLD-1 cells (4 h DMSO and PlaB). Two replicates are shown. (R1: $n = 9997$ RI zones, R2: $n = 9597$ RI zones). Statistical test: Wilcoxon test. ****$P < 0.0001$. Box plot: minimal-to-maximal value, box center line: median, bounds of box: interquartile (25 and 75%). (**F**) Example view of SIRV-normalized POINT-seq on *SPOPL* gene adjacent to P-NA zone in NELF-C-AID DLD-1 cells (4 h DMSO and IAA). RI zone is highlighted in gray. RI score: 42.8401. Both (+) and (−) strands are shown. (**G**) Box plots of TIs in POINT-seq in NELF-C-AID DLD-1 cells (4 h vs 0 h IAA). Genes in P-NA ($n = 6651$) and P-A (all ($n = 5878$), all without RDH ($n = 5849$), and RDH ($n = 29$) genes) zones were analyzed. Statistical test: Kruskal–Wallis test. ns: not significant, ****$P < 0.0001$. Box plot: minimal-to-maximal value, box center line: median, bounds of box: interquartile (25 and 75%).

 © The Author(s)

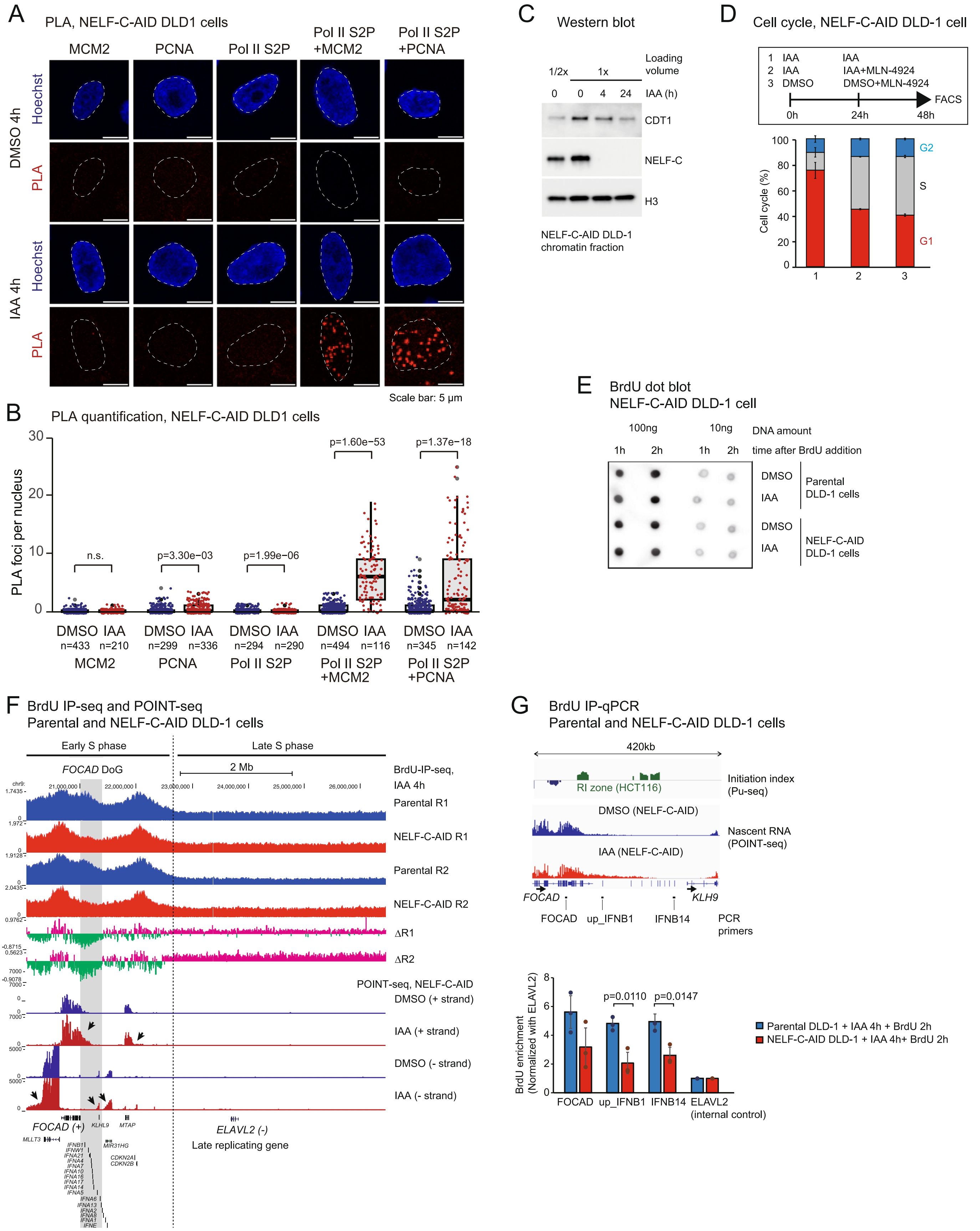

A PLA, NELF-C-AID DLD1 cells
MCM2 PCNA Pol II S2P Pol II S2P +MCM2 Pol II S2P +PCNA
DMSO 4h
IAA 4h
Hoechst
PLA
Scale bar: 5 μm
B PLA quantification, NELF-C-AID DLD1 cells
PLA foci per nucleus
n.s.
p=3.30e−03
p=1.99e−06
p=1.60e−53
p=1.37e−18
DMSO IAA
n=433 n=210 MCM2
n=299 n=336 PCNA
n=294 n=290 Pol II S2P
n=494 n=116 Pol II S2P +MCM2
n=345 n=142 Pol II S2P +PCNA
C Western blot
Loading volume
1/2x 1x
0 0 4 24 IAA (h)
CDT1
NELF-C
H3
NELF-C-AID DLD-1 chromatin fraction
D Cell cycle, NELF-C-AID DLD-1 cell
1 IAA IAA
2 IAA IAA+MLN-4924
3 DMSO DMSO+MLN-4924
FACS
0h 24h 48h
Cell cycle (%)
G2 S G1
E BrdU dot blot NELF-C-AID DLD-1 cell
100ng 10ng DNA amount
1h 2h 1h 2h time after BrdU addition
DMSO IAA Parental DLD-1 cells
DMSO IAA NELF-C-AID DLD-1 cells
F BrdU IP-seq and POINT-seq Parental and NELF-C-AID DLD-1 cells
Early S phase
Late S phase
FOCAD DoG
2 Mb
BrdU-IP-seq, IAA 4h
Parental R1
NELF-C-AID R1
Parental R2
NELF-C-AID R2
ΔR1
ΔR2
POINT-seq, NELF-C-AID
DMSO (+ strand)
IAA (+ strand)
DMSO (- strand)
IAA (- strand)
FOCAD (+)
ELAVL2 (-)
Late replicating gene
G BrdU IP-qPCR Parental and NELF-C-AID DLD-1 cells
420kb
RI zone (HCT116)
Initiation index (Pu-seq)
DMSO (NELF-C-AID)
IAA (NELF-C-AID)
Nascent RNA (POINT-seq)
FOCAD KLH9
FOCAD up_IFNB1 IFNB14 PCR primers
BrdU enrichment (Normalized with ELAVL2)
p=0.0110 p=0.0147
Parental DLD-1 + IAA 4h + BrdU 2h
NELF-C-AID DLD-1 + IAA 4h+ BrdU 2h
FOCAD up_IFNB1 IFNB14 ELAVL2 (internal control)

Figure EV8. Acute depletion of NELF-C protein causes a conflict between transcription and replication and suppresses DNA replication.

(**A**) Representative images of PLA with the indicated antibodies after 4 h DMSO and IAA in NELF-C-AID DLD-1 cells. Blue: Hoechst, Red: PLA. Scale bar size is 5 μm. (**B**) Box plots of the PLA foci per nucleus after 4 h DMSO and IAA in NELF-C-AID DLD-1 cells. The PLA was performed with indicated antibodies. Box plots show the median (center line) and interquartile range (box, 25th–75th percentiles); whiskers indicate 1.5×IQR. Dots represent individual nuclei. Statistical test: Brunner–Munzel test. Statistical test: Wilcoxon rank-sum test. *P* values are shown. not significant (n.s.). (**C**) Western blot of chromatin fraction of NELF-C-AID DLD-1 cells (0, 4, and 24 h IAA) using the indicated antibodies. (**D**) Cell cycle (%) of 24 h DMSO or MLN-4924 treated NELF-C-AID DLD-1 cells. The cells were pre-treated with IAA or DMSO for 24 h. Error bars represented the mean ± SEM (biological replicates, $n = 3$). (**E**) BrdU dot blot assay to evaluate global DNA synthesis. (**F**) BrdU-IP-seq of two biological replicates (R1 and R2) for FOCAD gene regions in parental and NELF-C-AID DLD-1 cells treated with IAA for 4 h. IP efficiencies (IP/input) are shown. BrdU plus (magenta) and minus (green) zones in Parental minus NELF-C-AID (Δ). Normalized POINT-seq profiles for (+) and (−) strands in NELF-C-AID DLD-1 cells treated with IAA for 0 h (DMSO) or 4 h (IAA) are shown. Pol II transcription termination defect are indicated by arrows. FOCAD DoG region is highlighted by gray. Early and Late S phase are separated by dashed line. (**G**) BrdU-IP-qPCR analysis to assess local DNA synthesis rates in region exhibiting termination defect. The top panel shows the locations of primer sets used for qPCR. The bottom panel displays the IP/input values for each locus, normalized to the *ELAVL2* locus, a late-replicating region that is not expected to replicate at the onset of the S phase (ELAVL2 locus = 1). Error bars represented the mean ± SEM (biological replicates, $n = 3$). *P* values are indicated.

 © The Author(s)

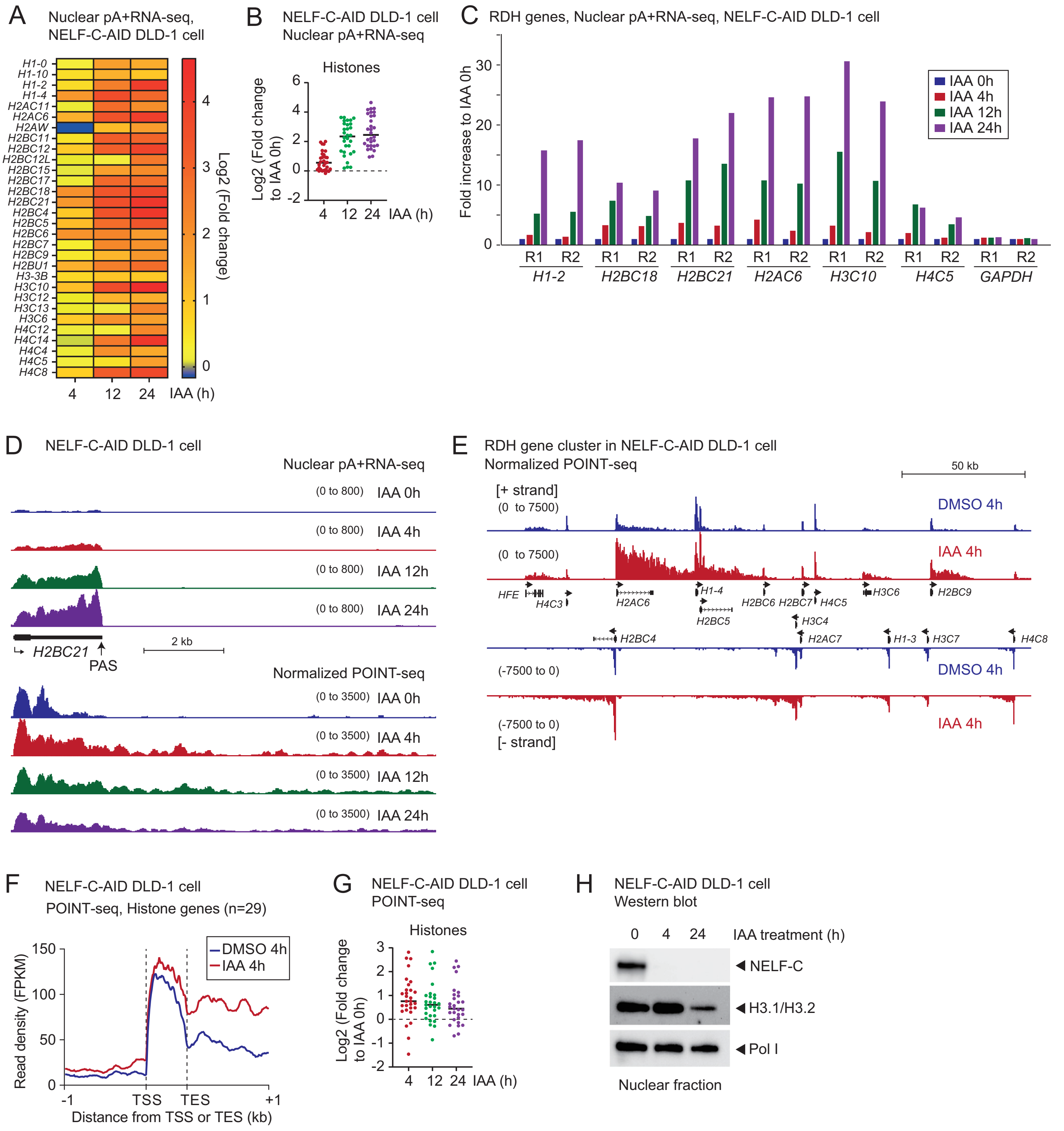

Figure EV9. Loss of NELF-C protein induces Pol II transcription readthrough and pA-tailed RNA (pA + RNA) production of replication-dependent histone (RDH) genes.

(**A**) Heatmap analysis of nuclear pA+ RNA-seq in NELF-C-AID DLD-1 cells (4, 12, and 24 h IAA). Fold change to 0 h IAA is displayed in the indicated color. (**B**) Log_2 fold change of nuclear pA+ RNA-seq signals of RDH genes ($n = 29$) in NELF-C-AID DLD-1 cells (4, 12, and 24 h IAA to 0 h). (**C**) Fold change of RNA expression level (two biological replicates) of the indicated RDH genes in NELF-C-AID DLD-1 cells (0, 4, 12, and 24 h IAA). *GAPDH* gene expression was not changed. (**D**) Example view of nuclear pA+ RNA-seq and SIRV-normalized POINT-seq on H2BC21 gene in NELF-C-AID DLD-1 cells (0, 4, 12, and 24 h IAA). PAS is indicated with an arrow. (**E**) View of POINT-seq of RDH gene cluster of NELF-C-AID DLD-1 cells (4 h vs 0 h IAA). Both (+) and (−) strands are shown. (**F**) Metagene analysis of POINT-seq on RDH genes ($n = 29$) of NELF-C-AID DLD-1 cells (4 h vs 0 h IAA). (**G**) Log_2 fold change of SIRV-normalized POINT-seq signals on RDH genes ($n = 29$) in NELF-C-AID DLD-1 cells (4, 12, and 24 h IAA). (**H**) Western blot of NELF-C-AID DLD-1 nuclear fraction (0, 4, and 24 h IAA) using the indicated antibodies. Pol I is analyzed as a loading control.

© The Author(s)
